# Biomimetic Non-Heme Iron-Catalyzed Epoxidation of Challenging Terminal Alkenes Using Aqueous H_2_O_2_ as an Environmentally Friendly Oxidant

**DOI:** 10.3390/molecules24173182

**Published:** 2019-09-01

**Authors:** Anja Fingerhut, Jorge Vargas-Caporali, Marco Antonio Leyva-Ramírez, Eusebio Juaristi, Svetlana B. Tsogoeva

**Affiliations:** 1 Department of Chemistry and Pharmacy, Institute of Organic Chemistry I and Interdisciplinary Center for Molecular Materials (ICMM), Friedrich-Alexander University Erlangen-Nürnberg, Nikolaus-Fiebiger-Straße 10, 91058 Erlangen, Germany; 2 Department of Chemistry, Centro de Investigación y de Estudios Avanzados, Av. Instituto Politécnico Nacional 2508, 07360 Ciudad de México, Mexico; 3 El Colegio Nacional, Donceles # 104, Centro Histórico, 06020 Ciudad de México, Mexico

**Keywords:** non-heme iron-catalysis, peptide-like ligands, enantioselective epoxidation, terminal olefins, hydrogen peroxide oxidant

## Abstract

Catalysis mediated by iron complexes is emerging as an eco-friendly and inexpensive option in comparison to traditional metal catalysis. The epoxidation of alkenes constitutes an attractive application of iron(III) catalysis, in which terminal olefins are challenging substrates. Herein, we describe our study on the design of biomimetic non-heme ligands for the in situ generation of iron(III) complexes and their evaluation as potential catalysts in epoxidation of terminal olefins. Since it is well-known that active sites of oxidases might involve imidazole fragment of histidine, various simple imidazole derivatives (seven compounds) were initially evaluated in order to find the best reaction conditions and to develop, subsequently, more elaborated amino acid-derived peptide-like chiral ligands (10 derivatives) for enantioselective epoxidations.

## 1. Introduction

Epoxides are subunits within many natural products or essential biologically active molecules. Epoxides also represent valuable building blocks, as they are widely used for the synthesis of fine chemicals and pharmaceuticals due to the broad range of modifications they can undergo [1,2,3,4,5,6,7]. In this regard, iron complexes offer potential advantages over other coordination complexes typically employed in epoxidation reactions because iron is an abundant and relatively non-toxic metal, so in principle, this element can provide environmentally friendly and economical catalysts for the synthesis of bulk epoxides [8]. Nature provides numerous efficient examples of iron-based catalysts, such as for example oxidative enzymes. The synthetic iron complexes mimicking the ability of these enzymes to promote oxidation processes are usually described as bioinspired catalysts and these can either be heme (porphyrin) systems or non-heme iron complexes. In particular, cytochrome P450 and other peroxidases serve as model enzymes for designing oxidation catalysts such as metalloporphyrins [9,10,11].

While previously studied heme iron catalytic systems were not found to be highly enantioselective [11], non-heme iron complexes have facilitated the access towards new promising enantioselective oxidizing catalysts. This goal can be achieved by fine tuning the basic structure of the non-heme ligands and with it, their electronic and steric properties. Thus, the combination of an iron center with a given chiral ligand should result in an efficient and low-cost catalyst for asymmetric synthesis [12]. 

**Figure 1:**
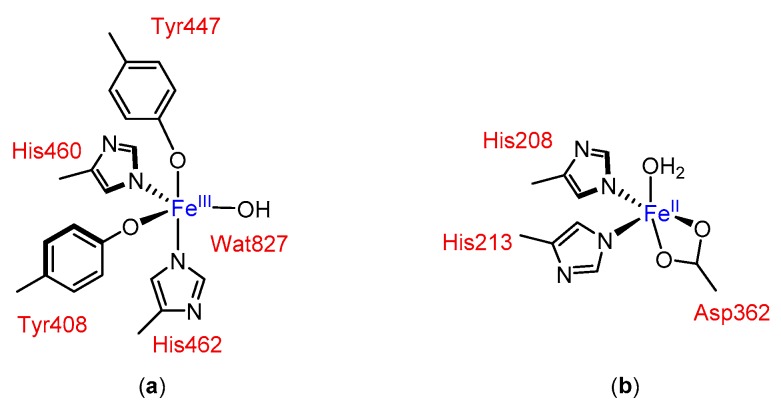
Examples of active sites in non-heme iron oxygenases: (**a**) in protocatechuate 3,4-dioxygenase [13] and (**b**) in naphthalene dioxygenase [14,15].

In principle, non-heme iron catalytic complexes for oxidation reactions can be modelled with the base on the active site of oxygenases, where imidazole and carboxylate enzymic residues (derived from e.g., His and Asp) ligate to the iron center and create a chiral environment (see Figure 1). 

In a general synthetic strategy, the simple combination of an iron salt with slightly modified naturally occurring chiral amines, carboxylic acids or amino acids can mimic the active site of an enzyme to achieve the goal of inducing an enantioselective oxidation.

**Figure 2:**
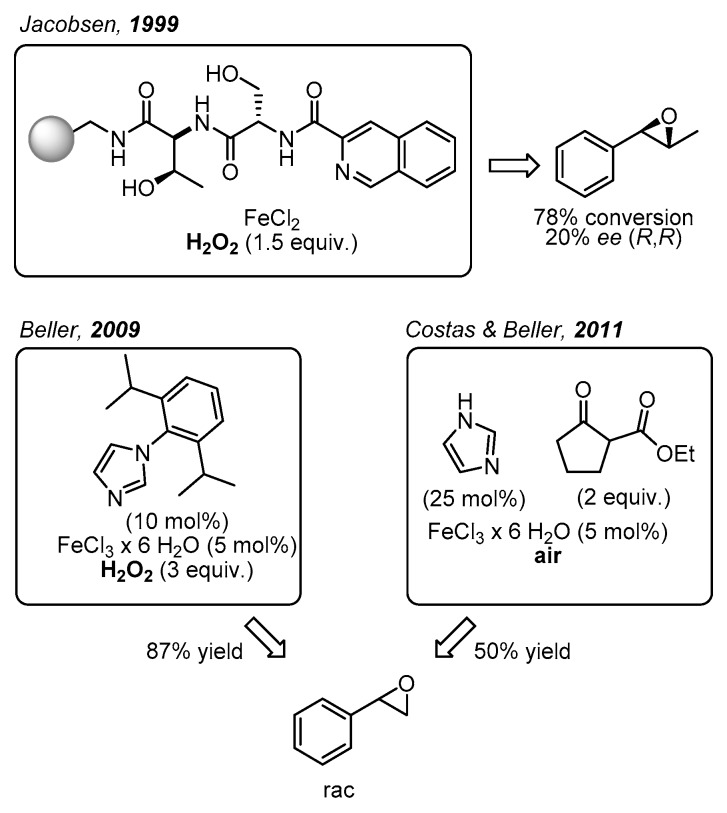
Biomimetic non-heme iron-catalyzed epoxidations reported by Jacobsen [18], Beller [17], Costas and Beller [16].

Initially, the task described above was carried out in a non-enantioselective approach [16,17]. Subsequently, in 1999 Jacobsen and co-workers came up with one of the main breakthroughs in the area of enantioselective epoxidations catalyzed by non-heme iron complexes, which were not only bioinspired but also biomimetic (Figure 2) [18]. Specifically, these workers demonstrated the catalytic potential of a library of polymer supported peptide-like ligands, which acted in combination with a variety of metal ions including iron salts. With *trans*-β-methylstyrene as model substrate, H_2_O_2_ as oxidant, and FeCl_2_ as iron source up to 78% conversion was recorded in up to 20% enantioselectivity. Even though the coordination structure of the most efficient catalyst was not provided, it was proposed that the peptide-based ligand bearing an N_2_O motif probably formed a biomimetic N_2_OFeCl_2_ complex. Further catalytic approaches towards epoxide formation with non-heme iron complexes and H_2_O_2_ or O_2_ as environmentally friendly oxidants have been reported [19,20]. For example, in 2007 Beller and co-workers developed a remarkable method for the asymmetric epoxidation of aromatic alkenes using H_2_O_2_ as oxidant [21,22]. The chiral iron catalysts were generated in situ from FeCl_3_·6H_2_O, chiral amino-sulfonamides, and pyridine-2,6-dicarboxylic acid (H_2_Pydic) as an additive [23]. Various alkenes were evaluated as substrates; however, only sterically hindered olefins were oxidized with high enantioselectivity [8]. In particular, terminal alkenes afforded products with 8–26% ee.

Furthermore, Beller and co-workers developed various non-enantioselective non-heme iron catalytic systems for olefin epoxidation with H_2_O_2_, where the catalyst was also formed in situ. In particular, small ligands with biomimetic coordination motifs were applied. Illustrative examples are benzylamine derivatives in combination with pyridin-2,6-dicarboxylic acid (H_2_Pydic) [23], or various imidazole derivatives (Figure 2) [17,24,25]. These catalytic systems were able to afford very good yields of racemic non-terminal and terminal epoxides. In this context, in 2011 Beller, Costas, and co-workers reported a biomimetic iron catalyst formed in situ for epoxidation reactions, using air as the oxidant (Figure 2) [16]. Interestingly, by applying a β-keto ester as co-substrate, the O_2_ activation was similar to the activation process observed in co-substrate-dependent non-heme oxygenases.

To this date, one of the most successful families of non-heme iron catalysts developed for asymmetric epoxidation reactions with H_2_O_2_ is the one presenting bioinspired octahedral complexes coordinated to a *bis*-amino-*bis*-pyridine ligand. These catalytic systems provide a *cis*-α-topology and are chiral at the metal center as consequence of the chiral aliphatic diamine backbone. Nevertheless, these catalytically active complexes are less closely modeled on enzymes and are not as readily available as the other previously mentioned examples. Nevertheless, the groups of Que [26,27], Costas [28,29,30], Sun [31,32], Bryliakov and Talsi [33] could obtain very promising results with respect to the yields and enantioselectivities. Regarding the enantioselective epoxidation of terminal olefins, the octahedral complexes afforded moderate yields and enantioselectivities.

In view of the abovementioned advantages, non-heme iron catalysis is a promising epoxidation method for eventual large-scale applications in industry, especially in combination with H_2_O_2_ as convenient oxidant. Indeed, this strategy is simple, inexpensive, and environmentally benign since H_2_O is the only released side-product [34,35]. As evidenced by the previously mentioned examples, terminal olefins were found to be challenging substrates for enantioselective non-heme iron catalytic systems. Nevertheless, terminal olefins represent an important class of substrates; for example, their chiral epoxides are widespread building blocks in the synthesis of β-blockers, HIV protease inhibitors, and polyol and polyene antibiotics, among others [2,3,4,5,6,36]. The routes by which terminal epoxides are obtained using non-heme iron complexes and H_2_O_2_ are still rare [31,37,38]. Therefore, the development of new and simple enantioselective biomimetic non-heme iron catalytic systems for the epoxidation of the challenging terminal alkenes using highly desirable oxidizing agents such as H_2_O_2_ constitutes an attractive and ambitious task.

Herein, we disclose a promising approach towards the preparation of valuable but challenging terminal oxiranes *via* enantioselective epoxidation with H_2_O_2_ by applying imidazole based peptide-like ligands in combination with a source of iron(III), representing a highly biomimetic catalyst.

## 2. Results and Discussion

2-Vinylnaphthalene was chosen as model substrate for the enantioselective epoxidation of terminal olefins. It turned out to be a challenging but important substrate owing to its bulkiness and relevance as precursor in drug synthesis [36,39,40]. To the best of our knowledge, among all non-heme iron catalysts employing H_2_O_2_ as the oxidant, only the complex described by Beller and co-workers which was generated in situ from pyrrolidine, H_2_Pydic, and FeCl_3_∙6H_2_O, succeeded in oxidizing 2-vinylnaphthalene with 40% yield, but afforded the desired product as a racemic mixture [41].

**Figure 3:**
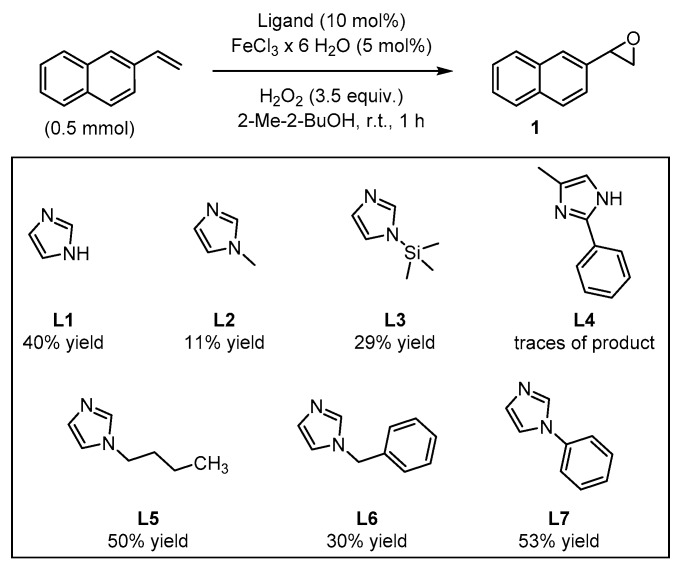
Screening of imidazole-based ligands: yields of the epoxide products were determined *via* ^1^H-NMR using pyrazine as internal standard.

In order to test their potential in the preparation of suitable catalysts, several imidazole derivatives with varying substitution patterns were screened as achiral ligands for iron(III) (Figure 3). The catalysts were formed in situ from 5 mol% FeCl_3_∙6H_2_O and 10 mol% of the coordinating imidazole derivative, generating the catalytically active iron species which enables oxygen transfer from H_2_O_2_ to the substrate, affording the target epoxide. This procedure for the in situ formation of the catalytic system is known to be time-saving, economic, and ecologically friendly, avoiding a laborious preliminary synthetic manipulation of precatalysts [42,43]. The addition of H_2_O_2_ was realized by means of a syringe pump over the entire reaction period of 1 h. Isolation of the terminal epoxides by column chromatography had to be avoided because SiO_2_ was found to catalyze the rearrangement of the product to give the corresponding aldehyde (see Supplementary Materials). Yield determination was carried out *via* ^1^H-NMR measurements using pyrazole as internal standard. The parent unsubstituted imidazole **L1** as reference ligand provided the target epoxide with 40% yield (Figure 3).

By comparison, *N*-methylimidazole **L2** and *N*-(trimethylsilyl)-imidazole **L3** afforded the epoxide with 11% and 29% yields, respectively. In the case of the 2,4-disubstituted imidazole derivative **L4**, only traces of the product could be observed. By contrast, *N*-butylimidazole **L5** afforded 50% yield of the desired product, even when analogous *N*-substituted imidazoles **L2** and **L3** showed modest catalytic activity. Furthermore, *N*-benzylimidazole **L6**, led to a comparable result relative to **L3** with 30% yield of epoxide. The best result was achieved with *N*-phenylimidazole **L7**, which afforded 53% yield of the epoxide product, surpassing the pyrrolidine/H_2_Pydic system of Beller et al. [41]. Further screening of reaction conditions, such as variation of the reaction temperature, substrate concentration, reaction time, or adding alternative acidic additives led in some cases to a decrease in the yield (see Supplementary Materials). Even the use of a co-catalyst was unsuccessful in the goal to improve the yield of the oxidation reaction. Interestingly, when the reaction was carried out under N_2_ atmosphere, only traces of the product were detected (see Supplementary Materials). This observation suggests the participation of the surrounding air in the formation of the catalytically active species. Initially, it could be suggested the formation of a self-assembled µ-oxo-iron(III) dimer as the catalytically active species, as it was previously discussed by Jacobsen and Stack [44,45]; however, later on Que and co-workers demonstrated that this class of dimer would not be efficient in promoting the oxidation and instead, the formation of this species would correspond to a dead-end intermediate [46]. In situ generation of the catalyst prior to substrate addition to further ensure self-assembly had no significant impact on yield (see Supplementary Materials).

**Figure 4:**
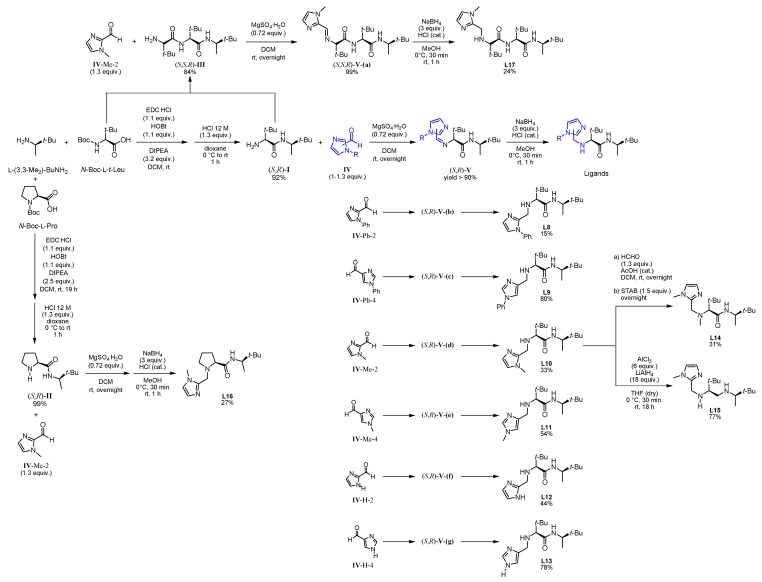
Family tree of chiral derivatives synthesized from (*R*)-3,3-dimethylbutan-2-amine.

Based on this preliminary screening of derivatives of simple imidazoles as constituents of potential ligands, the most promising one *N*-phenylimidazole **L7** was selected as reference point for the design of more elaborated chiral ligands and their application in enantioselective epoxidation of 2-vinylnaphthalene. In particular, inspired by Nature (see Figure 1 in the Introduction) we combined **L7** with a small chiral peptide-like moiety in order to obtain new ligand **L8** (Figure 4). It was anticipated that this ligand would enable a selective coordination on the iron metal, imitating a catalytically active enzyme pocket which in turn could effectively orient the substrate toward the activated oxygen, leading to the selective formation of one enantiomeric epoxide. Similarly, the straightforward synthesis of **L8**–**L13** and **L17** was carried out *via* EDC coupling using *N*-Boc-protected l-*tert*-leucine and (*R*)-3,3-dimethyl-2-butylamine as starting materials. Subsequently, the deprotected primary amine was subjected to a reductive amination with the required imidazole carboxaldehyde derivatives, forming the different imidazole-based peptide-like ligands. In the case of **L16**, *N*-Boc-protected l-proline was used for EDC coupling instead of l-*tert*-leucine. The methylation reaction of **L10** was carried out with formaldehyde and sodium triacetoxyborohydride to form ligand **L14**. In order to generate ligand **L15**, a reduction of ligand **L10** with AlCl_3_-LiAlH_4_ was carried out. Thus, we obtained a series of stable ligands providing a typical [*N*,*N*]-binding motif for the coordination to a metal center comparable to that in salalen ligands [47,48]. It is worth mentioning that ligand **L10** has already been employed as chiral promoter in the enantioselective silyl protection of vicinal diols [49,50,51] or cyclic and acyclic triols [52]. 

**Figure 5:**
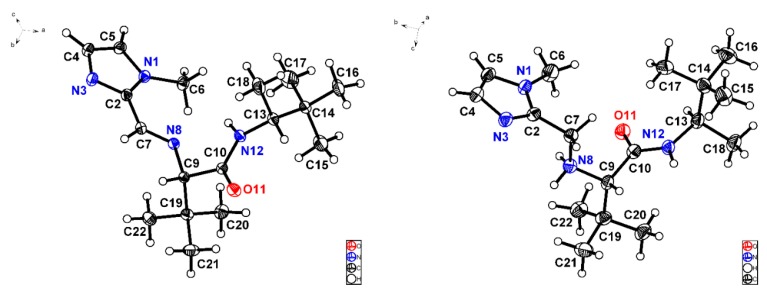
X-ray structures corresponding to imine (*S*,*R*)-**V**-(d) [C10-N12 1.336 (2) Å, C10-O11 1.227 (2) Å] (**left**) and ligand **L10** [C10-N12 1.335 (6) Å, C10-O11 1.238 (5) Å] (**right**) depicted with ellipsoids at 30% probability.

On the other hand, it was possible to obtain suitable monocrystals from imine (*S*,*R*)-**V**-(d) and its subsequent product of reduction, ligand **L10**, to collect X-ray diffraction data (Figure 5). It can be appreciated that iminium (N8) and amide (N12) nitrogen atoms in imine (*S*,*R*)-**V**-(d) are both oriented in the same direction (θ_N8-C9-C10-N12_ = −8.7°). By contrast, N12 in the amino amide **L10** is accommodated in opposite direction relative to N8 (θ_N8-C9-C10-N12_ = 127.3°). Another salient structural difference is given by C-N bond distances, for instance C7-N8 is equal to 1.265 Å in the imine (Figure 5, left) while in the amine C7-N8 is 1.456 Å. N8-C9 presents the same length, around 1.46 Å, in both compounds. Furthermore, amine protons could be located by Fourier difference analysis given the existence of hydrogen bonds. Likewise, nitrogen atoms N3, N8, and N12 in amino amide **L10** are found forming a sort of cavity, which could suggest its suitability to coordinate an iron cation.

**Figure 6:**
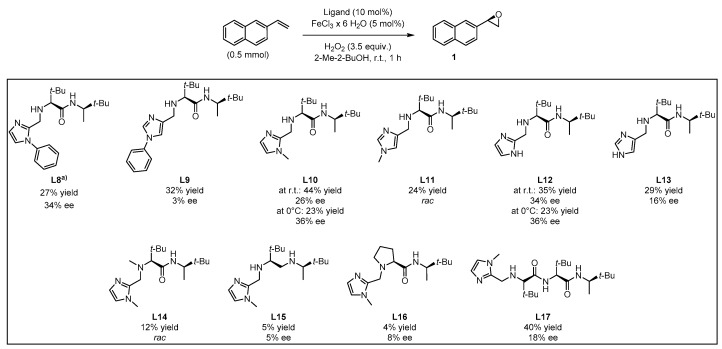
Screening of chiral peptide-like imidazole-based ligands. Yields determined *via* ^1^H-NMR with pyrazine as internal standard; ee values determined *via* chiral HPLC measurement. (a) 0.33 mmol of 2-vinylnaphthalene was used.

In this context, the newly designed ligand **L8** (1,2-substituted imidazole) contains two *tert*-butyl groups, which might induce a substantial effect on enantioselectivity when being compared with other residues. Indeed, the bulkiness of the *tert*-butyl group is particularly effective as stereodifferentiating structural element, as it was discussed for the salen-type ligand in Jacobsen’s catalyst [53]. Application of ligand **L8** to our model reaction led to the isolation of the target epoxide with 27% yield and 34% ee. Likewise, derivative **L9**, with a 1,4-substitution pattern, was considered in order to investigate the effect of the linkage position at the imidazole on the reaction outcome [17]. However, no significant improvement of yield was observed and actually, a decrease of enantiomeric excess was evident. *N*-methylated ligands **L10** (1,2-substituted imidazole) and **L11** (1,4-substituted imidazole), both sharing a common coordination motif, were also screened in order to examine the influence of the *N*-imidazole residue. While the methylated achiral ligand led to a decrease in yield (compare ligand **L2** and **L7**, Figure 3), ligand **L10** afforded an improved yield of 44%, with 26% ee. When the reaction was carried out at lower temperature (0 °C) the enantiomeric excess could be improved up to 36%, although the yield dropped to 23% (Figure 6). On the other hand, ligand **L11** (1,4-substituted imidazole) led to the racemic epoxide in 24% yield. A similar trend relative to that found with the phenylated (**L8** and **L9**) and methylated (**L10** and **L11**) ligands was observed with *N*-unsubstituted ligands **L12** and **L13**. With ligand **L12**, 35% yield and 34% ee were recorded. Again, the enantioselectivity was slightly improved to 36% when carrying out the reaction at 0 °C, although the yield decreased. As it could have been anticipated, ligand **L13** turned out to be less efficient than **L12**, with an observed decrease in yield and enantioselectivity. The corresponding screening indicated that ligand **L10** was most promising, both in terms of yield and enantioselectivity. To exclude the possibility that coordination sites of the iron center were blocked when using a 2:1 molar ratio of ligand to iron, which would suppress H_2_O_2_ activation, the amount of ligand **L10** was reduced to a 1:1 molar ratio (see Supplementary Materials). However, the yield was reduced, and the enantiomeric excess remained unchanged. For further experiments, a 1:1 molar ratio of ligand to iron source with 10 mol% ligand was deemed appropriate for efficient catalysis.

Ligands **L14**, **L15**, and **L16** were designed in order to examine the effect of structural modifications on ligand **L10**. This analysis provided insight on the factors that enable coordination to the iron center and thereby the activation of the H_2_O_2_ oxidant. Indeed, methylation of the secondary aliphatic amine, which is part of the proposed coordination motif, led to ligand **L14** tested as catalyst in the model oxidation of 2-vinylnaphthalene, affording the target epoxide in a poor 12% yield and in a racemic manner. It becomes then obvious that the secondary amine in **L10** plays a key role in enantioselective oxygen transfer to the substrate. Furthermore, examination of ligand **L15** lacking the carbonyl function, only afforded traces of the desired product with a negligible enantiomeric excess. This led to the conclusion that either the carbonyl oxygen or the nitrogen activated by the amide function participate in the formation of the catalytically active iron complex.

When the more rigid amino acid l-proline was incorporated instead of l-*tert*-leucine in compound **L16**, only traces of the product were observed in low enantiomeric excess. This corroborated the intuitive assumption that neither the tertiary nitrogen of the proline moiety nor the less flexible backbone enables coordination of the ligand to the iron center, in this way preventing H_2_O_2_ activation and, hence, oxygen transfer. To improve the beneficial influence of the l-*tert*-leucine derived moiety, a second residue was introduced thus obtaining ligand **L17** as an extended version of **L10**. The enantioselective epoxidation applying ligand **L17** led to 40% yield, which falls in the range exhibited by ligand **L10**. Nevertheless, the observed enantiomeric excess decreased from 26% to 18%.

For further studies of the catalytic system, the influence of (*S*)-(+)-mandelic acid as additive in the reaction employing ligand **L10** was investigated. Chiral carboxylic acids or carboxylic acids in general are known for their positive effect as co-catalyst in organo- and metal catalyzed reactions [54,55]. As coordinating ligand, the carboxylic acid facilitates the O-O cleavage and promotes the formation of the active iron oxo species [28,44,45]. However, in the present system there was no change in enantioselectivity when adding 5 mol% (*S*)-(+)-mandelic acid to the model reaction system (see Supplementary Materials). The model epoxidation reaction applying achiral ligand **L7** (Figure 3) showed a decrease of yield to 26% by adding 5 mol% of (*S*)-(+)-mandelic acid as well, and 2-vinylnaphtyl oxide (**1**) was formed as a racemic mixture (see Supplementary Materials). Under modified reaction conditions with reduced amount of H_2_O_2_ and reduced temperature, the addition of 5 mol% (*S*)-(+)-mandelic acid led to a slight increase of the enantiomeric excess (Table 1, Entry 1).

To investigate the effect of (*S*)-(+)-mandelic acid and the contribution to the complex formed in situ, the amount was raised to 15 mol% and the quantity of ligand **L10** was reduced to 5 mol% because of the available coordination positions. However, the effect of the modified reaction conditions was small, and the values of yield and enantiomeric excess were in the same range, as already described for the prior reaction system with ligand **L10** at 0 °C (compare Table 1, Entry 2 with Figure 6, ligand **L10**). Doubling the amount of catalyst to 20 mol% (Entry 3, Table 1) resulted in 43% yield and 38% enantiomeric excess, which was so far the best result concerning yield and enantioselectivity. Nevertheless, taking the reaction mass efficiency [56] into account, the aforementioned result was not as promising for further investigation as the already demonstrated approach at room temperature (Figure 6, ligand **L10**).

A further screening of terminal and non-terminal, electron rich and electron deficient olefins should elucidate the steric and electronic properties of the catalyst by drawing inferences from substrate preference (Table 2). All tested substrates were found to be oxidized with lower yield than 2-vinylnaphthalene. The reason for this can be on the one hand the good accessibility of the electron-rich terminal double bond to the iron center due to reduced electronic repulsion, and on the other hand, due to an enhanced stabilization from the bigger naphthyl fragment. Compared to 2-vinylnaphthyl oxide, the styrene oxide was obtained with lower yield (Table 2, Entry 2). Styrene derivatives with electron withdrawing groups like 4-nitrostyrene and 1,2-dichloro-4-vinylbenzene showed lower yield for their corresponding epoxides compared to unsubstituted styrene (Table 2, Entry 3 and Entry 4).

On the other hand, styrene and its aryl-substituted derivatives (EWGs) led to their corresponding epoxides with enantioselectivities that fall in the same range of those observed with 2-vinylnaphthalene oxide (Table 2, Entries 2–4). Unexpectedly, 4-methoxystyrene with its electron donating group, was oxidized with 5% yield and only 14% enantiomeric excess (Table 2, Entry 5). The result was surprising because of the expected tendency that electron rich terminal olefins were favored by the catalyst. This behavior excludes the simple explanation that the oxidizing iron species formed during the catalytic process is of electrophilic nature. The more sterically demanding double bond of α-methyl styrene led to 22% yield and decreased enantioselectivity with 16% ee (Table 2, Entry 6). Furthermore, a benzyl substituted terminal olefin gave just traces of product (Table 2, Entry 7), indicating less activity of the catalyst for non-conjugated double bonds, even when they are terminal. Thus, we propose that an aryl moiety directly adjacent to the double bond is highly required to enable the catalyzed epoxidation process.

On the other hand, when β-methylstyrene was used as substrate, the yield of the corresponding epoxide was 32%, so this result was in the range of that pertaining to the unsubstituted styrene, even though the enantiomeric excess of 50% was quite high compared to all the other electron-rich olefins which were tested (Table 2, Entry 8). Thus regarding the enantioselectivity, the epoxidation system presented herein exceeded Jacobsen’s polymer-supported peptide-based catalyst (Figure 2) [18]. Still no conclusion about the electronic nature of the oxidizing iron species could be drawn, taking the moderate yield of β-methylstyrene oxide into account. However, the methyl group at the β position may direct the substrate in a more defined way towards the H_2_O_2_ activating iron center within the chiral catalyst surrounding. In the case of *trans*-stilbene as representative of nonterminal symmetrically substituted olefins, the yield was 27% with 16% enantiomeric excess (Table 2, Entry 9). The lower ee in comparison to styrene oxide could be explained by the difficult access of the catalyst to the double bond and with it the suppressed transfer of chiral information during the oxygen transferring process. When the catalytic system was applied on *trans*-chalcone as an example of a more electron-deficient nonterminal olefin, only product traces were observed with an ee value of 27% (Table 2, Entry 10). However, the enantioselectivity was raised to 51% under the modified reaction conditions (described previously in Table 1) by using (*S*)-(+)-mandelic acid additive (Table 2, Entry 11). Even though the yield was quite low, the result was interesting due to the significant influence of the (*S*)-(+)-mandelic acid, but only for this substrate. Therefore, we reasoned that the carboxylic acid did not only favor the formation of the iron oxo species, contrary to what is described in literature [28,44,45]. Possibly, an additional interaction between the chiral acid additive and the carbonyl function of chalcone raises the enantiomeric excess of the chalcone oxide. Although the substrate screening revealed that 2-vinylnaphthalene was the most appropriate starting material (regarding yield) the two non-terminal substrates chalcone and β-methylstyrene showed the most promising results with respect to enantioselectivity.

It is worthy of mention that diverse attempts to obtain a suitable monocrystal from **L10**∙FeCl_3_ and **L10**∙FeCl_3_∙[(*S*)-mandelic acid] in different solvents or mixtures were not fruitful, obtaining in both cases a dark amber vitreous solid. In order to get insights into the catalyst formation process, some spectroscopic experiments were conducted. For instance, UV/Vis-spectroscopy measurements were carried out (see Supplementary Materials). The comparison of the absorption spectra corresponding to the individually measured compounds, ligand **L10** and FeCl_3_∙6H_2_O in 2-Me-2-BuOH, with the spectrum of the mixture (ligand **L10** combined with FeCl_3_∙6H_2_O in 2-Me-2-BuOH) shows a distinct shift of the characteristic FeCl_3_∙6H_2_O absorption maxima. This indicates that the metal interacts with ligand **L10** forming a complex. As a further UV/Vis spectrum suggests, an additional structural change of the previously generated iron complex was taking place when H_2_O_2_ was added. This was attributed to the formation of an oxygen transferring iron species. Likewise, it was possible to identify a species with a mass equivalent to 514.5 Da from a solution of the complex in methanol by means of LC–ESI-MS. Besides, infrared experiments (ATR) comparing pure **L10** versus the preformed complex **L10**∙FeCl_3_ showed a noticeable variation in the amide carbonyl band, shifting from 1651 cm^−1^ in **L10** to 1599 cm^−1^ in the complex, suggesting that the oxygen preferably coordinates instead of the amide nitrogen (see Supplementary Materials). In another IR experiment, it could also be appreciated that it was not the mandelic acid carbonyl group which was participating in the iron coordination but the hydroxyl oxygen since the intense -OH characteristic band (3440 cm^−1^) disappeared in the spectra of the final complex. Thus, the mandelic acid carbonyl group only slightly varied (from 1710 to 1735 cm^−1^, though its transmittance percentage seemed to be reduced). Similarly, the amide carbonyl band in the complex remained practically unchanged when adding mandelic acid (from 1599 to 1602 cm^−1^).

**Figure 7:**
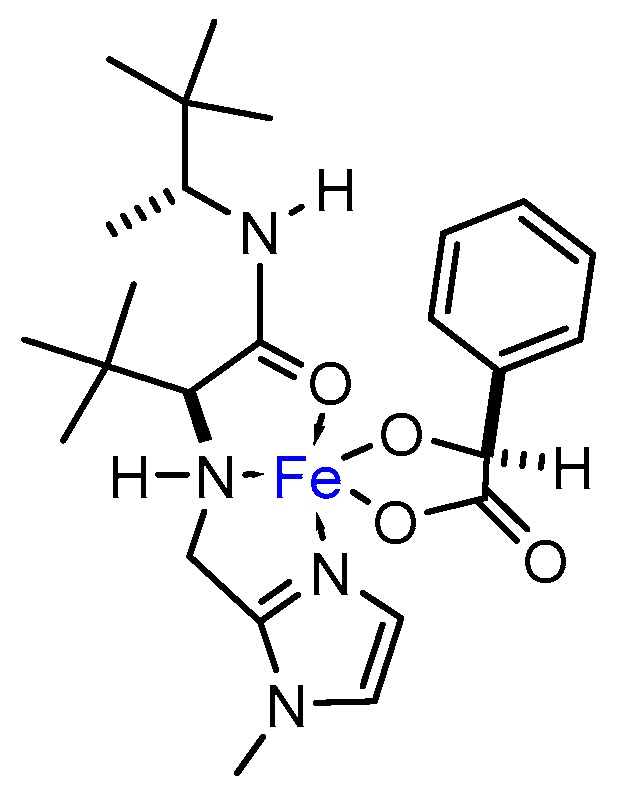
Possible coordination complex participating as catalyst in the model epoxidation reaction.

In addition, NMR experiments were run in methanol-*d*_3_ solvent. Even though the paramagnetism of the metal complex made the accumulation of the sample difficult, spectra of **L10** significantly changed upon addition of FeCl_3_∙6H_2_O [complex concentration = 0.027 M, **L10** and Fe(III) in equimolar amount]. In particular, a significant broadening of signals in ^1^H-NMR was observed. Furthermore, in presence of Fe(III) a remarkable shift for both *tert*-butyl fragments took place, resulting in a single broad proton signal at 0.2099 ppm. This contrasted with the clearly defined signals for each fragment of *tert*-butyl in the pure ligand (0.937 and 0.915 ppm). The *tert*-butyl signal broadened even more when adding an equimolar amount of (*S*)-mandelic acid, shifting down to −0.325 ppm. These observations may give evidence of the formation of a species in which the degrees of freedom of the *tert*-butyl fragments are restricted, i.e., a coordination complex. In accordance with the changes observed by ^1^H-NMR, the pair of clearly defined signals for each fragment of *tert*-butyl in ^13^C-NMR (25.56 and 26.05 ppm) led to an intense signal when adding an equimolar amount of FeCl_3_∙6H_2_O (28.6 ppm) which shifted again to 30.14 ppm in presence of (*S*)-mandelic acid. Another salient difference in chemical shifts was that corresponding to the methyl bonded to the stereogenic carbon, moving from 14.95 ppm in the pure ligand up to 17.87 ppm in the final complex. Less noticeable were the ∆δ corresponding to the carbonyl carbon: 173.74 ppm in **L10** to 171.91 ppm in the final complex including (*S*)-mandelic acid. On the other hand, noteworthy changes were also observed for the imidazol fragment, wherein 121.78 and 125.46 ppm shifted to 130.27 and 137.64 ppm, respectively, and C*_ipso_* in **L10** (146.32 ppm) moves down to 144.65 ppm in the final complex, suggesting that the imidazol fragment could be actively participating in coordination to iron metal. Figure 7 depicts a plausible structure for the intermediate iron complex.

## 3. Materials and Methods 

### 3.1. General Information

Chemicals purchased from commercial sources were used without further purification. All solvents were purified by distillation, dried according to standard procedures or were purchased in HPLC-quality. Preparative (flash) column chromatography was performed on Acros Silica gel 60 (0.035–0.070 mm, 60 Å) as stationary phase. All products were dried in high vacuum (10-3 bar). Thin layer chromatography (TLC) was performed on precoated aluminium silica gel SIL G/UV254 plates (Macherey-Nagel & Co., Düren, NRW, Germany). ^1^H-NMR (^13^C-NMR) spectra were recorded at room temperature on a Bruker Avance 300 or 400 or JEOL JNM GX 400 spectrometer. All chemical shifts are given in ppm scale and refer to the non-deuterized proportion of the solvent. NMR raw data was processed with the program MestReNova. Maldi Mass spectra were recorded with Shimadzu Biotech AXIMA Confidence. ESI Mass spectra were recorded with a Bruker Daltonik maXis 4G, a Bruker Daltonik micrOTOF II focus or an Agilent 6120 Quadrupole LCMS System. HPLC spectra were recorded at room temperature on an Agilent Technologies 1200 Series HPLC system. As a stationary phase, the following columns were utilized: IA, IB, IC, AS, OD. IR spectra were recorded on a Varian IR-660 apparatus. The Absorption is indicated in wave numbers (cm^−1^). Optical rotations were determined on a PerkinElmer polarimeter, model 341, *λ* = 546 nm.

### 3.2. General Catalytic Procedure

#### 3.2.1. Catalysis without Additive

To a solution of FeCl_3_ ·6 H_2_O (6.8 mg, 25.0 µmol, 5 mol%) in 9 mL 2-Me-2-BuOH, an imidazole based ligand (50.0 µmol, 10 mol%) was added at room temperature under air. After addition of the olefin (500 µmol), H_2_O_2_ (30 wt% in H_2_O, 170 µL, 1.50 mmol) mixed with 830 µL 2-Me-2-BuOH was added *via* syringe pump over a 1 h period. The reaction was quenched with 50 µL saturated aqueous Na_2_SO_3_ solution. After extraction with CH_2_Cl_2_, the organic phases were combined and evaporated to dryness. The crude mixture was filtered through a SiO_2_-plug (1 cm, eluted with CH_2_Cl_2_). In order to determine the yield *via* ^1^H-NMR, pyrazine was added in a defined amount as an internal standard. Afterwards, purification was carried out by means a preparative TLC plate (hexane/ethyl acetate) for the HPLC sample preparation in order to determine enantioselectivity.

#### 3.2.2. Catalysis with (*S*)-(+)-Mandelic Acid

A mixture of FeCl_3_·6 H_2_O (5–10 mol%), ligand **L1** (5–20 mol%), and (*S*)-(+)-mandelic acid (5–15 mol%) in 1.6 mL of 2-Me-2-BuOH was stirred under air at room temperature for 1 h. Afterwards, 2-vinylnaphtalene (25.6 mg, 166 µmol) was added and the reaction mixture was cooled to 0 °C. H_2_O_2_ (30 wt% in H_2_O, 24 µL, 249 µmol) was added *via* a syringe pump over 1 h. Then the reaction mixture was stirred for additional 2 h at 0 °C. The reaction was quenched with 30 µL of saturated aqueous Na_2_SO_3_ solution. After extraction with CH_2_Cl_2_, the organic phases were combined and evaporated to dryness under reduced pressure. The crude mixture was filtered through a SiO_2_-plug (1 cm, eluted with CH_2_Cl_2_). In order to determine yield *via* ^1^H-NMR, pyrazine was added in a defined amount as an internal standard. Afterwards, purification was carried out with preparative TLC plate (hexane/ethyl acetate) for the HPLC sample preparation in order to determine enantioselectivity.

#### 3.2.3. Substrate and Racemic Product References

Olefins like 1-nitro-4-vinylbenzene [57] or 1,2-dichloro-4-vinylbenzene [58] were prepared according to literature procedure.

The obtained spectroscopic data of 2-phenyloxirane (**2**) [59] and 2,3-diphenyloxirane (**9**) [42] was in accordance with commercially available 2-phenyloxirane and data from literature.

Oxidation to racemic phenyl(3-phenyloxiran-2-yl)methanone (**10**) [60] was prepared according to literature procedure.

For determination of yield and enantioselectivity of product resulting from 4-methoxystyrene, the volatile epoxide (**5**) was further converted to the corresponding β-aminoalcohol *via* aminolysis by adding 18 equiv. of isopropylamine (instead of addition of saturated aqueous Na_2_SO_3_ soln.) and stirring the reaction mixture for 18 h at 50 °C. Then the solvent was evaporated and the raw material was purified *via* column chromatography [36]. The racemic reference of 2-(isopropylamino)-1-(4-methoxyphenyl)ethan-1-ol was synthesized, as described in procedure 3.2.1 using 1-phenylimidazole as achiral ligand. The obtained spectroscopic data of 2-(isopropylamino)-1-(4-methoxy-phenyl)ethan-1-ol was in accordance with literature [61].

### 3.3. General Procedure of Olefin Epoxidation with m-CPBA

A mixture of olefin (490 µmol-7.69 mmol) and NaHCO_3_ (0.9-1.2 equiv.) was stirred in DCM at room temperature. After addition of *m*-CPBA (77% purity, 0.9–1.2 equiv.) the reaction mixture was stirred overnight. The obtained spectroscopic data for 2-(naphthalen-2-yl)oxirane (**1**) [62], 2-(4-nitrophenyl)oxirane (**3**) [63], 2-(3,4-dichlorophenyl)oxirane (**4**) [36], 2-Methyl-2-phenyloxirane (**6**) [62], 2-Benzyloxirane (**7**) [64] and 2-Methyl-3-phenyloxirane (**8**) [65] was in accordance with literature.

#### 3.3.1. Synthesis of Ligands

Synthesis of Boc-l-tert-Leucine [66], Boc-l-Proline [67], 1-phenyl-1*H*-imidazole-2-carbaldehyde [68], 3-methyl-4-formylimidazol, and 1-methyl-4-formylimidazol [69] were prepared according to reported procedures.

#### 3.3.2. Synthesis of l-Proline or l-*tert*-Leucine Based Amines

(*S*)-2-Amino-*N*-[(*R*)-3,3-dimethylbutan-2-yl]-3,3-dimethylbutan-amide, (*S*,*R*)-**I**. *N*-Boc-l-*tert*-Leucine (1.99 g, 9.30 mmol) and (*R*)-3,3-dimethyl-2-butylamine (1.23 mL, 9.30 mmol) were dissolved in 30 mL of DCM. To the solution EDC·HCl (1.96 g, 10.2 mmol), HOBt (1.57 g, 10.2 mmol), and DIPEA (3.84 g, 5.20 mL, 29.7 mmol) were added. The reaction mixture was stirred overnight at room temperature; then, 10 mL of 10% citric acid was added to quench the reaction. The organic layer was separated and washed with saturated aqueous NaHCO_3_ and brine, dried over anhydrous sodium sulfate, filtered, and concentrated under reduced pressure to afford a white solid. The residue was dissolved in 10 mL of dioxane, cooled to 0 °C. Then, 1 mL of 12 M hydrochloric acid was added dropwise. The mixture was stirred at room temperature over 1 h and the solution was concentrated under reduced pressure. The residue was taken up in 5 mL of water and 2 M NaOH solution was added until no further increase in the amount of precipitate was observed. The resulting solution was washed with DCM and brine and then dried over anhydrous sodium sulphate. Purification *via* column chromatography (SiO_2_, DCM/MeOH 98:2) afforded the title compound as a white solid (1.83 g, 8.50 mmol, 92%). The amine was used without further detailed characterization. **^1^H-NMR** (300 MHz, CDCl_3_, rt): δ [ppm] = 6.63 (d, *J* = 8.4 Hz, 1H), 3.81 (dq, *J*_1_ = 6.8 Hz, *J*_2_ = 9.7 Hz, 1H), 3.07 (s, 1H), 1.03 (d, *J* = 6.8 Hz, 3H), 0.99 (s, 9H), 0.88 (s, 9H). **^13^C-NMR** (75 MHz, CDCl_3_, rt): δ [ppm] = 172.7, 64.8, 52.4, 34.1, 34.0, 26.9, 26.3, 16.1. **HR-MS** (ESI) *m*/*z*: [M + H]^+^ calcd. for C_12_H_27_N_2_O: 215.211790; found: 215.212414.

(*S*)-*N*-[(*R*)-3,3-Dimethylbutan-2-yl]pyrrolidine-2-carboxamide [(*S*,*R*)-**II**]. *N*-Boc-l-proline (620 mg, 2.88 mmol) and (*R*)-3,3-dimethyl-2-butylamine (0.382 mL, 2.88 mmol) were dissolved in 12 mL DCM. EDC (608 mg, 3.17 mmol), HOBt (428 mg, 3.17 mmol), and DIPEA (931 mg, 1.25 mL, 7.20 mmol) were added to the reaction mixture. The mixture was stirred over 19 h at room temperature. Afterwards, 4 mL of 10% citric acid was added to the mixture. The organic layer was removed and washed with saturated solution of sodium bicarbonate, brine, dried over anhydrous sodium sulfate, filtrated and concentrated under reduced pressure to afford a yellow oil. This oil was cooled to 0 °C and later, HCl/dioxane (2.1 mL of a 4 M solution) was added over the course of 1 h. The mixture was warmed to room temperature over 1 h, and the solution was concentrated *in vacuo*. The unpurified product was dissolved in water and the solution was basified (pH = 12) by adding a solution of NaOH 3 N. The resulting solution was extracted with DCM, washed with brine and dried over anhydrous sodium sulfate. After removal of the solvent *in vacuo*, raw material of (*S*,*R*)-**II** was obtained as a white solid and used without further purification or characterization.

(*S*)-2-Amino-*N*-{(*S*)-1-[((*R*)-3,3-dimethylbutan-2-yl)amino]-3,3-dimethyl-1-oxobutan-2-yl}-3,3-dimethylbutanamide [(*S*,*S*,*R*)-**III**]. Boc-L-*tert*-Leucine [(*S*)-*N*-Boc-*t*Leu, 1.99 g, 9.30 mmol] and (*S*)-2-Amino-*N*-((*R*)-3,3-dimethylbutan-2-yl)-3,3-dimethylbutan-amide [(*S*,*R*)-**I**, 2.15 g, 9.30 mmol] were dissolved in DCM (30 mL). EDC·HCl (1.96 g, 10.2 mmol), HOBt (1.57 g, 10.2 mmol), and DIPEA (3.84 g, 5.20 mL, 29.7 mmol) were added to the solution. The reaction mixture was stirred overnight at room temperature; later, 10 mL of 10% citric acid was added to quench the reaction. The organic layer was separated and washed with saturated aqueous NaHCO_3_ and 10 mL of brine, dried over Na_2_SO_4_, filtered, and concentrated under reduced pressure to afford a white solid. The residue was dissolved in dioxane (10 mL), cooled to 0 °C, and 1 mL of HCl (12 M) was added dropwise. The mixture was stirred at room temperature over 1 h and the solution was concentrated under reduced pressure. The residue was taken up in 5 mL of water, and 2 M NaOH solution was added until no further increase in the amount of precipitate was observed. The resulting mixture was extracted with DCM, the organic layer was separated, washed with brine and dried over anhydrous Na_2_SO_4_. The product was evaporated to dryness in vacuo to afford the title compound as a white powder (2.56 g, 84% yield). The raw material was used without further purification or detailed characterization.

#### 3.3.3. Synthesis of Imidazole Based Aldehyde

1-Phenyl-1*H*-imidazole-4-carbaldehyde, **IV**-Ph-4. CuI (93.0 mg, 488 µmol), 1,10-phenanthroline (88.0 mg, 488 µmol), tripotassium phosphate (1.56 g, mmol, 7.35 mmol), and 4-imidazolecarboxaldehyde (330 mg, 3.43 mmol) were placed under inert conditions in a flask with 5 mL of dry DMF. After stirring the mixture at room temperature for 10 min, bromobenzene (386 mg, 258 µL, 2.46 mmol) was added. The reaction mixture was heated to 120 °C over 65 h. After cooling to room temperature, the solution was diluted with ethyl acetate (20 mL) and filtered through silica gel. The organic phase was washed with brine and then dried over MgSO_4_. Following to concentration in vacuo, the raw product was recrystallized from DCM and pentane, which afforded the pure product as a white solid (112 mg, 650 µmol; 26% yield). The obtained spectroscopic data were in accordance with literature [66]. ^1^H-NMR (300 MHz, CDCl_3_, rt): δ [ppm] = 9.94 (s, 1H), 7.92 (dd, J_1_ = 14.0 Hz, J_2_ = 1.3 Hz, 2H), 7.69–7.29 (m, 5H).

#### 3.3.4. Synthesis of Imidazole Based Peptide Like Ligand

General Procedure (GP1): Reductive Amination

The l-Proline or L-*tert*-Leucine-based amine was dissolved in DCM (2 M), followed by the addition of the imidazole based aldehyde (1–1.3 equiv.) and MgSO_4_·H_2_O (1 g/5 mL solvent). The mixture was stirred overnight at room temperature. Afterwards, solids were removed by filtration and the resulting solution was concentrated in vacuo. The residue was dissolved in MeOH (2 M) and the solution was cooled to 0 °C. NaBH_4_ (3 equiv.) and a catalytic amount of conc. HCl were added to this solution. The mixture was stirred for 30 min at 0 °C, and 1 h at room temperature; after this period, an equal volume of saturated NaHCO_3_ solution was added to quench the reaction. After washing with DCM and brine, the organic phase was separated, dried over anhydrous Na_2_SO_4_ and concentrated in vacuo to afford the raw product. Purification of the resulting raw material was carried out *via* column chromatography using DCM and MeOH, eluted compound might be subsequently recrystallized from DCM and pentane.

Ligand **L8**. The synthesis was carried out according to GP1. The target compound was obtained from (*S*)-2-Amino-*N*-[(*R*)-3,3-dimethylbutan-2-yl]-3,3-dimethylbutanamide [(*S*,*R*)-**I**, 47.6 mg, 222 µmol] and 1-phenyl-1*H*-imidazole-2-carbaldehyde (**V**-Ph-2, 49.7 mg, 289 µmol) after purification *via* column chromatography (SiO_2_, DCM/MeOH 98:2) as a white solid (12.1 mg, 33.0 µmol; 15%). **^1^H-NMR** (300 MHz, CD_2_Cl_2_, rt): δ [ppm] = 7.51–7.36 (m, 5H), 7.08 (d, *J* = 1.3 Hz, 1H), 7.05 (d, *J* = 1.3 Hz, 1 H), 6.50 (br, *J* = 9.1 Hz, 1H), 3.80–3.74 (m, 1H), 3.72 (d, *J* = 14.0 Hz, 1H), 3.53 (d, *J* = 13.9 Hz, 1H), 2.72 (s, 1H), 0.95 (s, 9H), 0.86 (s, 9H), 0.83 (d, *J* = 6.8 Hz, 3H). **^13^C-NMR** (100 MHz, CD_2_Cl_2_, rt): δ [ppm] = 171.7, 146.4, 137.8, 129.9, 128.7, 128.2, 125.9, 121.4, 72.5, 52.8, 44.7, 34.2, 34.0, 27.4, 26.5, 16.2. **HR-MS** (ESI) *m*/*z*: [M + H]^+^ calcd. for C_22_H_35_N_4_O: 371.28054; found: 371.28081; *m*/*z*: [M + Na]^+^ calcd. for C_22_H_34_N_4_NaO: 393.26248; found: 393.26263. **[α]^D^_20_** = −64° (*c* = 0.15, DCM). **IR** (ATR, solid): *ṽ* [cm^−1^] = 3447, 3193, 3067, 2954, 2905, 2871, 1306, 1644, 1555, 1500, 1470, 1306, 1255, 1141, 1126, 1115, 1082, 962, 915, 818, 764, 734, 693, 569, 540.

Ligand **L9**. The synthesis was carried out according to GP1. The target compound was obtained from (*S*)-2-Amino-*N*-[(*R*)-3,3-dimethylbutan-2-yl]-3,3-dimethylbutanamide [(*S*,*R*)-**I**, 108 mg, 504 µmol] and 1-phenyl-1*H*-imidazole-4-carbaldehyde (**V**-Ph-4, 112 mg, 650 µmol) after purification *via* column chromatography (SiO_2_, DCM/MeOH 98:2) as a white solid (150 mg, 405 µmol; 80%). **^1^H-NMR** (300 MHz, CDCl_3_, rt): δ [ppm] = 7.77 (d, *J* = 1.4 Hz, 1H), 7.47–7.32 (m, 5H), 7.11 (s, 1H), 7.02 (d, *J* = 9.8 Hz, 1H), 3.85 (dq, *J*_1_ = 6.8 Hz, *J*_2_ = 9.8 Hz, 1H), 3.73 (d, *J* = 14.2 Hz, 1H), 3.51 (d, *J* = 13.9 Hz, 1 H), 2.82 (s, 1H), 2.08 (s, 1H), 1.05 (d, *J* = 6.8 Hz, 3H), 0.98 (s, 9H), 0.89 (s, 9H). **^13^C-NMR** (100 MHz, CDCl_3_, rt): δ [ppm] = 172.4, 141.9, 137.6, 135.7, 130.3, 127.8, 121.6, 115.9, 72.3, 52.9, 46.2, 34.4, 34.0, 27.8, 26.8, 16.6. **HR-MS** (ESI) *m*/*z*: [M + H]^+^ calcd. for C_22_H_35_N_4_O: 371.28054; found: 371.28086. **[α]^D^_20_** = −42° (*c* = 0.15, DCM). **IR** (ATR, solid): *ṽ* [cm^−1^] = 3326, 3054, 2958, 2870, 1648, 1600, 1505, 1475, 1365, 1306, 1251, 1130, 1067, 993, 967, 818, 757, 690, 522.

Ligand **L10**. The synthesis was carried out according to GP1. The target compound was obtained from (*S*)-2-Amino-*N*-((*R*)-3,3-dimethylbutan-2-yl)-3,3-dimethylbutanamide [(*S*,*R*)-**I**, 973 mg, 4.54 mmol] and 1-methyl-2-imidazolcarbaldehyde (500 mg, 4.54 mmol) after purification *via* column chromatography (SiO_2_, DCM/MeOH 98:2) and recrystallization (DCM/pentane) as a white solid (461 mg, 1.50 mmol; 33%). The obtained spectroscopic data was in accordance with literature [70]. **^1^H-NMR** (300 MHz, CDCl_3_, rt): δ [ppm] = 6.90 (d, *J* = 1.2 Hz, 1H), 6.78 (d, *J* = 1.2 Hz, 1H), 6.49 (br d, *J* = 9.6 Hz, 1H), 3.88 (dq, *J*_1_ = 9.8 Hz, *J*_2_ = 6.8 Hz, 1H), 3.77 (d, *J* = 13.9 Hz, 1H), 3.59 (s, 3H), 3.58 (d, *J* = 13.9 Hz, 1H), 2.65 (s, 1H), 2.14 (s, 1H), 1.03 (d, *J* = 6.8 Hz, 3H), 0.93 (s, 9H), 0.89 (s, 9H). **^13^C-NMR** (100 MHz, CDCl_3_, rt): δ [ppm] = 172.0, 146.1, 127.1, 121.1, 72.0, 52.7, 44.3, 33.9, 33.8, 32.6, 27.1, 26.4, 16.4.

Ligand **L11**. The synthesis was carried out according to GP1. The target compound was obtained from (*S*)-2-Amino-*N*-[(*R*)-3,3-dimethylbutan-2-yl]-3,3-dimethylbutanamide [(*S*,*R*)-**I**, 86.3 mg, 403 µmol) and 1-methyl-1*H*-imidazole-4-carbaldehyde (**V**-Me-4, 44.4 mg, 403 µmol) after purification *via* column chromatography (SiO_2_, DCM/MeOH 98:2) as a white solid (67.7 mg, 219 µmol; 54%). **^1^H-NMR** (300 MHz, CDCl_3_, rt): δ [ppm] = 7.30 (s, 1H), 7.00 (br d, *J* = 9.6 Hz, 1H), 6.67 (s, 1H), 3.79 (dq, *J*_1_ = 6.8 Hz, *J*_2_ = 9.8 Hz, 1H), 3.60 (d, *J* = 13.8 Hz, 1H), 3.58 (s, 3H), 3.36 (d, *J* = 14.1 Hz, 1H), 2.73 (s, 1H), 2.34 (s, 1H), 1.00 (d, *J* = 6.8 Hz, 3H), 0.91 (s, 9H), 0.84 (s, 9H). **^13^C-NMR** (100 MHz, CDCl_3_, rt): δ [ppm] = 171.9, 140.5, 137.6, 117.5, 71.7, 52.4, 45.7, 33.9, 33.4, 33.2, 27.4, 26.4, 16.1. **HR-MS** (ESI) *m*/*z*: [M + H]^+^ calcd. for C_17_H_33_N_4_O: 309.26489; found: 309.26512. **[α]^D^_21_** = −17° (*c* = 0.15, DCM). **IR** (ATR, solid): *ṽ* [cm^−1^] = 3327, 2956, 2870, 1618, 1508, 1462, 1365, 1307, 1232, 1160, 1131, 991, 816, 738, 618, 482.

Ligand **L12**. The synthesis was carried out according to GP1. However, in the first step, MeOH was employed instead of DCM. The target compound was obtained from (*S*)-2-Amino-*N*-[(*R*)-3,3-dimethylbutan-2-yl]-3,3-dimethylbutanamide [(*S*,*R*)-**I**, 108 mg, 504 µmol] and 2-imidazolecarboxaldehyde (**V**-H-2, 48.0 mg, 504 µmol). Purification of the resulting material by recrystallization (DCM/pentane) afforded the desired compound as a white solid (65.0 mg, 221 µmol; 44%). **^1^H-NMR** (300 MHz, CDCl_3_, rt): δ [ppm] = 6.91 (s, 2H), 6.70 (d, J = 9.3 Hz, 1H), 3.88 (d, *J* = 14.8 Hz, 1H), 3.84–3.76 (m, 1H), 3.56 (d, *J* = 14.8 Hz, 1H), 2.66 (s, 1H), 0.99 (d, *J* = 6.8 Hz, 3H), 0.93 (s, 9H), 0.85 (s, 9H). **^13^C-NMR** (100 MHz, CDCl_3_, rt): δ [ppm] = 172.4, 146.8, 121.7, 71.8, 52.8, 45.4, 34.0, 33.7, 27.1, 16.2. **HR-MS** (ESI) *m*/*z*: [M + H]^+^ calcd. for C_16_H_31_N_4_O: 295.249238; found: 295.249907. **[α]^D^_22_** = −79° (c = 0.15, DCM). **IR** (ATR, solid): *ṽ* [cm^−1^] = 3352, 3163, 3053, 2955, 2870, 1637, 1526, 1464, 1365, 1248, 1228, 1108, 988, 800, 739, 679, 627, 479.

Ligand **L13**. The synthesis was carried out according to GP1. The target compound was obtained from (*S*)-2-Amino-*N*-[(*R*)-3,3-dimethylbutan-2-yl]-3,3-dimethylbutanamide [(*S*,*R*)-**I**, 93.4 mg, 436 µmol] and 4-imidazolecarboxaldehyde (**V**-H-4, 54.5 mg, 567 µmol) without any further purification as a white solid (99.8 mg, 339 µmol; 78%). **^1^H-NMR** (400 MHz, CDCl_3_, rt): δ [ppm] = 7.55 (s, 1H), 6.98 (br, *J* = 9.0 Hz, 1H), 6.76 (s, 1H), 3.82 (dq, *J*_1_ = 6.8 Hz, *J*_2_ = 13.5 Hz, 1H), 3.75 (d, *J* = 14.0 Hz, 1H), 3.42 (d, *J* = 14.1 Hz, 1H), 2.71 (s, 1H), 1.04 (d, J = 6.8 Hz, 3H), 0.91 (s, 9H), 0.88 (s, 9H). **^13^C-NMR** (100 MHz, CDCl_3_, rt): δ [ppm] = 172.6, 136.1, 135.3, 116.4, 70.9, 52.8, 44.3, 34.0, 33.5, 27.3, 26.3, 16.2. **MS** (MALDI-TOF): *m*/*z* = 295 [M + H]^+^, 317 [M + Na]^+^. **HR-MS** (ESI) *m*/*z*: [M + H]^+^ calcd. for C_16_H_31_N_4_O: 295.24924; found: 295.24933. **[α]^D^_21_** = −21° (*c* = 0.15, DCM). **IR** (ATR, solid): *ṽ* [cm^−1^] = 3314, 3170, 3114, 2958, 2869, 1642, 1516, 1463, 1396, 1365, 1233, 1210, 1130, 987, 933, 818, 731, 661, 623, 495.

Ligand **L14**. Formaldehyde (37 wt% in H_2_O, 53.0 µL, 650 µmol, 1.3 equiv.) was added to **L13** (154 mg, 500 µmol) in 3 mL DCM. After addition of catalytic drops of glacial acetic acid, the reaction mixture was stirred overnight at room temperature. Then sodium triacetoxyborohydride (159 mg, 750 µmol) was added to the reaction mixture which was stirred again overnight. Afterwards the reaction was quenched with 10 mL saturated ammonium chloride solution, and stirred for 1.5 h. The organic layer was separated and washed with water, dried over Na_2_SO_4_, filtered and concentrated. The target compound was obtained as a white solid (50.0 mg, 155 µmol; 31% yield). **^1^H-NMR** (300 MHz, CDCl_3_, rt): *δ* [ppm] = 6.89 (s, 1H), 6.82 (s, 1H), 6.75 (d, *J* = 8.3 Hz, 1H), 3.89 (dq, *J* = 9.5, 6.8 Hz, 1H), 3.78–3.65 (m, 5H), 2.52 (s, 1H), 2.48 (s, 3H), 1.04 (d, *J* = 6.8 Hz, 3H), 0.96 (s, 9H), 0.80 (s, 9H). **^13^C-NMR** (100 MHz, CDCl_3_, rt): *δ* [ppm] = 168.7, 145.8, 126.6, 121.4, 70.7, 53.3, 52.6, 41.6, 35.0, 33.5, 32.7, 27.3, 26.7, 16.4. **MS** (MALDI-TOF): *m*/*z* = 323 [M + H]^+^. **HR-MS** (ESI) *m*/*z*: [M + H]^+^ calcd. for C_18_H_35_N_4_O: 323.280538; found: 323.281289. **[α]^D^_20_** = −168° (*c* = 0.15, DCM). **IR** (ATR, solid): *ṽ* [cm^−1^] = 3433, 3286, 2954, 2867, 2805, 1649, 1532, 1502, 1451, 1364, 1262, 1178, 1128, 1113, 1013, 965, 811, 743, 708, 667, 626, 472.

Ligand **L15.** AlCl_3_ (130 mg, 0.973 mmol) was suspended in 15 mL of dry THF under inert atmosphere cooled to 0 °C. Then 1 M LiAlH_4_ (2.9 mL, 2.920 mmol) was added and the reaction was stirred for 0.5 h at 0 °C. Afterwards, **L13** (50 mg, 0.162 mmol) dissolved in 3 mL was added of dry THF and the reaction mixture was stirred 18 h at room temperature. The reaction mixture was poured onto ice. After the emulsion reached room temperature, the mixture was filtered through a celite path, followed by a washing step of celite with warm ethyl acetate. The resulting filtrate was condensed, and the mixture was then extracted with ethyl acetate. The separated organic phase was treated with Na_2_SO_4_ and afterwards, concentrated under reduced pressure. The crude product was purified *via* column chromatography (SiO_2_, mobile phase gradient from CH_2_Cl_2_/(2.5% NH_4_OH in MeOH) 98:2 to CH_2_Cl_2_/(2.5% NH_4_OH in MeOH) 9:1. The pure product was obtained as a white solid (37 mg, 77% yield). **^1^H-NMR** (300 MHz, CDCl_3_, rt): δ [ppm] = 6.81 (d, *J* = 1.4 Hz, 1H), 6.76 (d, *J* = 1.4 Hz, 1H), 4.17–3.88 (m, 2H), 3.49 (s, 3H), 3.17 (dd, *J* = 11.2, 3.0 Hz, 1H), 2.99–2.71 (m, 2H), 2.64 (d, *J* = 6.8 Hz, 1H), 1.30 (d, *J* = 6.8 Hz, 3H), 1.07 (s, 9H), 0.93 (s, 9H). **^13^C-NMR** (100 MHz, CDCl_3_, rt): δ [ppm] = 147.45, 125.90, 121.05, 77.20, 66.10, 49.66, 45.28, 35.95, 34.23, 32.34, 26.58, 26.54, 12.68.

Ligand **L16**. The synthesis was carried out according to GP1. The target compound was obtained from unpurified raw material of (*S*)-*N*-[(*R*)-3,3-dimethylbutan-2-yl]pyrrolidine-2-carboxamide [(*S*,*R*)-**II**, 2.88 mmol] and 1-methyl-2-imidazolecarboxaldehyde (**V**-Me-2, 317 mg, 2.88 mmol) after recrystallization (DCM/pentane) as a white solid (229 mg, 790 µmol; 27%). **^1^H-NMR** (300 MHz, CDCl_3_, rt): *δ* [ppm] = 7.30 (d, *J* = 9.8 Hz, 1H), 6.92 (d, *J* = 1.2 Hz, 1H), 6.79 (d, *J* = 1.2 Hz, 1H), 3.93–3.69 (m, 3H), 3.63 (s, 3H), 3.23 (dd, *J*_1_ = 4.3 Hz, *J*_2_ = 10.1 Hz, 1H), 3.02 (dd, *J*_1_ = 4.4 Hz, *J*_2_ = 11.2 Hz, 1H), 2.64–2.55 (m, 1H), 2.22–1.67 (m, 4H), 0.93 (d, *J* = 6.8 Hz, 3H), 0.85 (s, 9H). **^13^C-NMR** (100 MHz, CDCl_3_, rt): *δ* [ppm] = 173.3, 145.0, 127.6, 121.1, 67.5, 54.4, 51.8, 50.6, 34.3, 32.8, 31.0, 26.1, 24.3, 16.2. **MS** (MALDI-TOF): *m*/*z* = 293 [M + H]^+^, 315 [M + Na]^+^, 607 [2M + Na]^+^. **HR-MS** (ESI) *m*/*z*: [M + H]^+^ calcd. for C_16_H_29_N_4_O: 293.233588; found: 293.234407. **[α]^D^_23_** = −49° (*c* = 0.15, DCM). **IR** (ATR, solid): *ṽ* [cm^−1^] = 3354, 3100, 2965, 2870, 2803, 1662, 1511, 1455, 1367, 1285, 1133, 1035, 976, 934, 766, 653, 469.

Ligand **L17**. The synthesis was carried out according to GP1. The target compound was obtained from unpurified raw material (*S*)-2-amino-*N*-{(*S*)-1-[((*R*)-3,3-dimethylbutan-2-yl)amino]-3,3-dimethyl-1-oxobutan-2-yl}-3,3-dimethylbutanamide [(*S*,*S*,*R*)-**IV**, 2.51 g, 7.70 mmol] and 1-methyl-2-imidazolecarboxaldehyde (**V**-Me-2, 1.10 g, 10.0 mmol, 1.3 equiv.) after purification *via* column chromatography (SiO_2_, DCM/2.5% NH_4_OH in MeOH gradient from 98:2 to 9:1) as a white solid (795 mg, 1.89 mmol; 24% yield). **^1^H-NMR** (400 MHz, CDCl_3_, rt): δ [ppm] = 7.55 (d, *J* = 9.8 Hz, 1H), 6.93 (d, *J* = 1.3 Hz, 1H), 6.80 (d, *J* = 1.3 Hz, 1H), 6.21 (d, *J* = 9.8 Hz, 1H), 4.32 (d, *J* = 9.8 Hz, 1H), 3.91–3.73 (m, 2H), 3.69 (s, 3H), 3.58 (d, *J* = 13.3 Hz, 1H), 2.79 (s, 1H), 1.05 (d, *J* = 6.9 Hz, 3H), 1.01 (s, 8H), 0.99 (s, 9H), 0.83 (s, 9H). **^13^C-NMR** (100 MHz, 3 rt): δ [ppm] = 172.49, 170.07, 146.10, 126.78, 121.29, 73.05, 60.72, 52.70, 44.51, 34.33, 34.09, 33.96, 33.01, 27.33, 26.82, 26.16, 16.11. **HR-MS** (ESI) *m*/*z*: [M + H]^+^ calcd. for C_23_H_44_N_5_O_2_: 422.348952; found: 422.349351. **[α]^D^_20_** = −19° (*c* = 0.15, DCM). **IR** (ATR, solid): *ṽ* [cm^−1^] = 3356, 3333, 3299, 2949, 2902, 2867, 1642, 1554, 1499, 1364, 1277, 1209, 1118, 918, 831, 758, 720, 705, 666, 628, 559, 483.

### 3.4. X-ray Crystallographic Studies

Single crystal X-ray diffraction data were collected using a Bruker D8 VENTURE system operated using the suite APEX 3 and the data collection temperature was controlled at 130 and 140 K using an Oxford Cryostream system. The crystal structures were solved using ShelxT version 2017 [71], and refined using ShelxL version 2018/1 [72].

Crystal refinement data for (*S*)-*N*-[(*R*)-3,3-dimethylbutan-2-yl]-3,3-dimethyl-2-[(*Z*)-(1-methyl-1*H*-imidazol-2-yl) methyleneamino]butanamide, (*S*,*R*)-V-(d). Colourless crystals, C_17_H_30_N_4_O, *M* = 306.45, orthorhombic, space group *P*2_1_2_1_2_1_, a = 6.9534(3), b = 14.0923(5), c = 18.9394(7) Å, α = 90, β = 90, γ = 90°, V = 1855.86 Å^3^, Z = 4, T = 140 K, refinement of 216 parameters on 3632 independent reflections out of 12,787 measured reflections (R_int_ = 0.0347) led to R_1_ = 0.0376 (I > 2*σ*(*I*)), wR_2_ = 0.0963 (all data).

Crystal refinement data for (*S*)-*N*-[(*R*)-3,3-dimethylbutan-2-yl]-3,3-dimethyl-2-[(1-methyl-1*H*-imidazol-2-yl)methyl-amino]butanamide, L10. Colourless crystals, C_17_H_33_N_4_O, *M* = 308.46, monoclinic, space group *P*2_1_, a = 8.0138(5), b = 13.0776(8), c = 9.4290(6) Å, α = 90, β = 108.996(4), γ = 90°, V = 934.357 Å^3^, Z = 2, T = 140 K, refinement of 212 parameters on 2823 independent reflections out of 12,635 measured reflections (R_int_ = 0.0854) led to R_1_ = 0.0528 (I > 2*σ*(*I*)), wR_2_ = 0.1363 (all data). 

Crystallographic data for compounds (*S*,*R*)-**V**-(d) and **L10** have been deposited with the Cambridge Crystallographic Data Centre with deposition numbers CCDC 1858309-1858310, respectively.

## 4. Conclusions

Herein, we report new enantioselective non-heme iron-catalyzed epoxidations using H_2_O_2_, by applying for the first time chiral imidazole based peptide-like ligands. We synthesized a set of easily accessible ligands for in situ generation of chiral iron(III) catalysts and studied their ability towards oxidation of terminal olefins, taking the imidazole substitution pattern, coordinating motif, and further important functionalities into account. In this context we developed one of the first biomimetic non-heme iron catalysts for epoxidation of challenging terminal alkenes.

## Figures and Tables

**Table 1:** Screening of chiral peptide-like imidazole-based ligands using (*S*)-(+)-mandelic acid as additive.

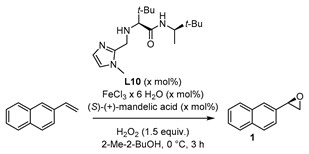					
Entry	Ligand L10 (mol%)	FeCl_3_∙ 6H_2_O (mol%)	(*S*)-(+)-mandelic acid (mol%)	Yield ^1^ (%)	ee ^2^ (%)
1	10	5	5	27	42
2	5	5	15	20	37
3	20	10	10	43	38

^1^ Yields determined *via* ^1^H-NMR with pyrazine as internal standard. ^2^ ee values determined *via* chiral HPLC measurement.

**Table 2:** Screening of substrates.

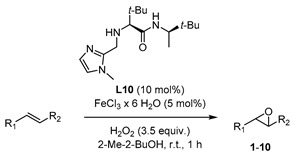			
Entry	Product	Yield ^1,2^	ee ^3^
1	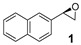	44	26 (*R*)
2	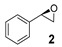	30	27 (*R*)
3	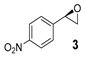	14	29 (*S*)
4	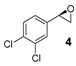	25	25 (*R*) ^4^
5	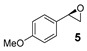	5 ^4^	14 (*R*) ^4^
6	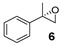	22	16 (*S*)
7	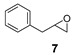	<5 ^5^	n.d. ^6^
8	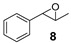	32	50
9	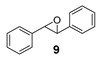	27	16 (*R*)
10	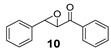	<5	27 (2*R,*3*S*)
11 ^7^	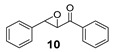	7	51 (2*R,*3*S*)

^1^ Yields determined *via* ^1^H-NMR with pyrazine as internal standard. ^2^ Results already described in literature for non-heme iron epoxidation employing H_2_O_2_ were contrasted in a comparative Table (see Supplementary Information). ^3^ ee values determined *via* chiral HPLC measurement. ^4^ ee value determined after derivatization: aminolysis with isopropylamine towards corresponding β-aminoalcohol. ^5^ detected *via* ^1^H-NMR and ESI-MS. ^6^ n.d.: not determined. ^7^ in situ catalyst generation with **L10** (5 mol%), FeCl_3_∙6H_2_O (5 mol%), (*S*)-(+)-mandelic acid (15 mol%), 1.6 mL 2-Me-2-BuOH, followed by the addition of alkene (0.166 mmol) and 2 eq. H_2_O_2_ *via* syringe pump at r. t. (3 h reaction).

## Supplementary Materials

The supplementary materials are available online.
