# Supplementary material for: Biomimetic Non-Heme Iron-Catalyzed Epoxidation of Challenging Terminal Alkenes Using Aqueous H2O2 as an Environmentally Friendly Oxidant
Source: Molecules. 2019 Sep 1;24(17):3182. doi: 10.3390/molecules24173182 (PMC6749192; doi:10.3390/molecules24173182)
Supplement: Supplementary file 1 [file molecules-24-03182-s001.pdf]

Supplementary Information

Biomimetic non-heme iron-catalyzed epoxidation of challenging terminal alkenes using aqueous H_2O_2 as an environmentally friendly oxidant

Anja Fingerhut [a], Jorge Vargas-Caporali [b], Marco Antonio Leiva-Ramírez [b], Eusebio Juaristi [b,c,] and Svetlana B. Tsogoeva [a,]**

[a] Department of Chemistry and Pharmacy, Institute of Organic Chemistry I and Interdisciplinary Center for Molecular Materials (ICMM), Friedrich-Alexander University Erlangen-Nürnberg, Nikolaus-Fiebiger-Straße 10, 91058 Erlangen, Germany. [b] Department of Chemistry, Centro de Investigación y de Estudios Avanzados, Av. Instituto Politécnico Nacional 2508, 07360 Ciudad de México, Mexico. [c] El Colegio Nacional, Donceles # 104, Centro Histórico, 06020 Ciudad de México, Mexico.

**E-Mail: juaristi@relaq.mx and svetlana.tsogoeva@fau.de*

Table of Contents

1 DETERMINATION OF YIELD VIA ^{1}H NMR USING PYRAZINE AS INTERNAL STANDARD 2

2 SCREENING OF REACTION CONDITIONS FOR NON-ENANTIOSELECTIVE EPOXIDATION 3

3 SCREENING OF REACTION CONDITIONS FOR ENANTIOSELECTIVE EPOXIDATION 4

4 REARRANGEMENT OF EPOXIDE 5

5 COMPARATIVE TABLE..6

6 ^{1} H NMR AND ^{13}C NMR SPECTRA OF NEW LIGANDS 8

7 HPLC CHROMATOGRAMS.. 17

8 INVESTIGATION OF IRON(III) COMPLEX VIA UV-VIS SPECTROSCOPY..................................... 25

9 INFRARED EXPERIMENTS OF IRON (III) COMPLEX.. 26

10 NMR EXPERIMENTS OF IRON(III) COMPLEX.. 27

11 MS-ESI EXPERIMENT OF IRON(III) COMPLEX...29

12 X-RAY CRYSTALLOGRAPHIC DATA... 30

1 Determination of yield via ^{1}H NMR using pyrazine as internal standard

Exemplary: Table 2, Entry 1:

^{1}H-NMR of reaction mixture after SiO_2-plug containing pyrazine as internal standard (m = 4.32 mg, n = 53.9 µmol)

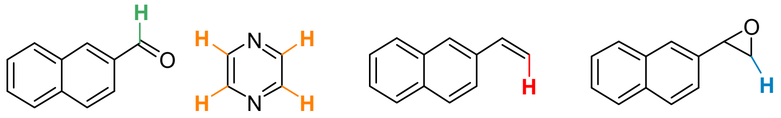

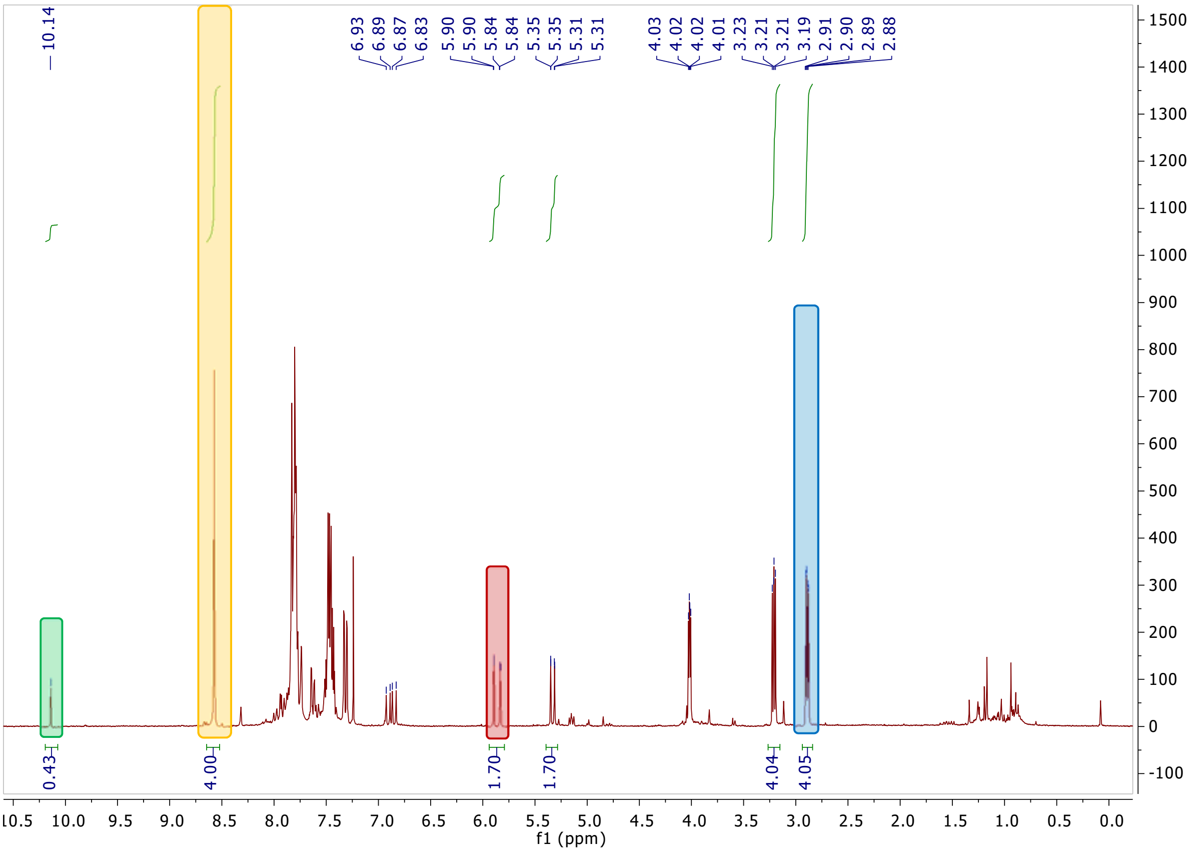

1H (pyrazine) ≙ 53.9 µmol

1H (epoxide) ≙ 53.9 µmol x 4.05 = 218 µmol (n (epoxide))

→ yield = n (epoxide) / n (alkene) = 218 µmol / 500 µmol = 0.44 → 44%

2 Screening of reaction conditions for non-enantioselective epoxidation

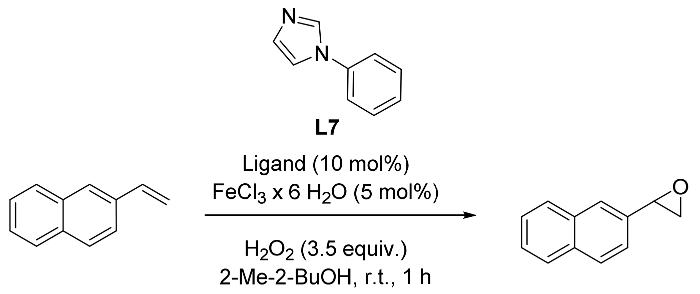

Table 1. Screening of reaction conditions.

Entry	Modification of reaction conditions	yield [%][a)]
1	-	53
2	1 h *in situ* catalyst generation (under inert conditions)	traces
3	1 h *in situ* catalyst generation (not under inert conditions)	54
4	1 h *in situ* catalyst generation and 5 mol% CH_3CO_2H	44
5	2 h reaction time and 2 h H_2O_2 addition.	45
6	15°C	22
7	0.05 M substrate	42
8	0.2 M substrate	25
9	addition of H_2O_2 over 3 h period	52
10	10 mol% catalyst loading	55
11	0°C	13
12	50°C	27
13	1 h H_2O_2 addition plus 15 h stirring	50
14	1 h H_2O_2 addition + 1 h stirring	53
15	+ 5 mol% H_2Pydic	19
16	+ 5 mol% mandelic acid	26
17	irradiation with cold light	52

a) Determined via pyrazine as internal standard.

3 Screening of reaction conditions for enantioselective epoxidation

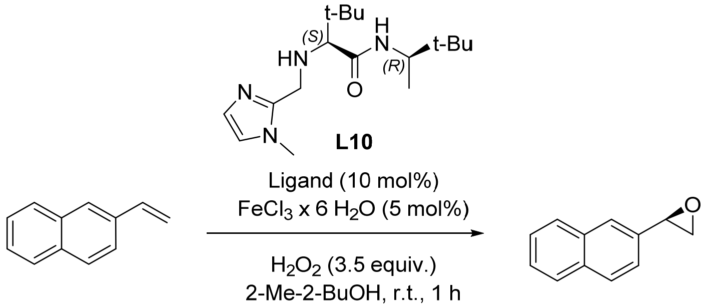

Table 2. Screening of reaction conditions.

Entry	Modification of reaction conditions	yield [%][a)]	ee-value (*R*) [%]
1	-	44	26
2	1 h *in situ* catalyst generation with air-bubbling	42	24
3	at 0°C	23	36
4	+ 5 mol% mandelic acid	32	27
5	**L10** (5 mol%)	31	26

a) Determined via pyrazine as internal standard.

4 Rearrangement of Epoxide

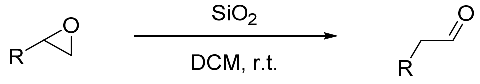

Table 3. First rearrangement attempts from epoxide towards corresponding aldehyde using SiO_2.

Entry	Substrate	Time	Temperature	Yield [%] [c]
1 [a), b)]	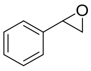	5 h	reflux	17
2	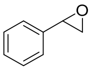	18 h	reflux	34
3 [a)]	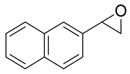	18 h	r.t.	29
4 [a)]	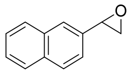	2.5 h	reflux	32

a) 3.33 g/mmol SiO_2; b) $CDCl_3$; c) Determined via pyrazine as internal standard.

5 Comparative Table

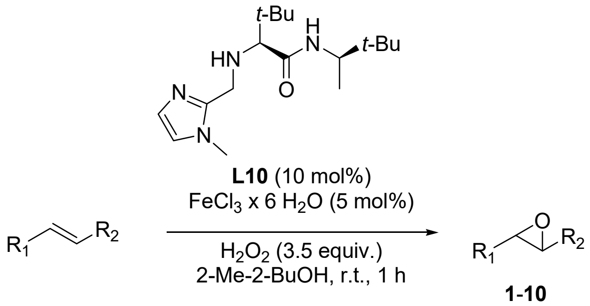

1-10

Table 4. Results obtained in this work contrasted with the results described in literature for non-heme iron-catalyzed epoxidation of the corresponding olefins.

		**Results of this work**		**Results described in literature**		
**Entry**	**Product**	**Yield a),b)**	**ee c)**	**Yield d)**	**ee d)**	**Ref.**
1	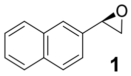 **1**	44	26 (*R*)	40	–	[1]
2	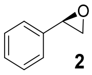 **2**	30	27 (*R*)	84<br>30<br>15	8<br>–<br>–	[2]<br>[3]<br>[4]
3	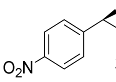 **3**	14	29 (S)	66<br>90	–<br>–	[5]<br>[6]
4	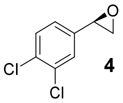 **4**	25	25 (*R*) e)	–	–	–
5	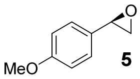 **5**	5 e)	14 (*R*) e)	82<br>70	–<br>–	[7]<br>[8]
6	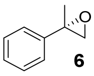 **6**	22	16 (S)	67	45	[9]
7	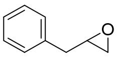 **7**	<5 f)	n.d. g)	60	–	[10]
8	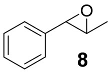 **8**	32	50	32<br>49	19<br>–	[9]<br>[11]
9	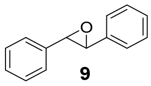 **9**	27	16 (*R*)	44<br>72	–<br>–	[3]<br>[8]
10	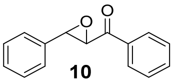 **10**	<5	27 (2*R*,3*S*)	38	90	[9]
11 h)	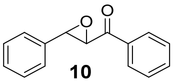 **10**	7	51 (2*R*,3*S*)	–	–	–

a) Yields determined via ^{1}H NMR with pyrazine as internal standard. b) Results described in this work. c) ee values determined via chiral HPLC measurement. d) Results described in literature for non-heme iron-catalyzed epoxidation employing H_2O_2 and miscellaneous ligands and/or additives (see references for further details). e) ee value determined after derivatization: aminolysis with isopropylamine towards corresponding β-aminoalcohol. f) Detected via ^{1}H-NMR and ESI-MS. g) n. d.: not determined. h) *in situ* catalyst generation with **L10** (5 mol%), $FeCl_3 \cdot 6H_2O$ (5 mol%), (*S*)-(+)-mandelic acid (15 mol%), 1.6 mL 2-Me-2-BuOH, followed by the addition of alkene (0.166 mmol) and 2 eq. H_2O_2 *via* syringe pump at r. t. (3 h reaction time).

References (see Table 4)

1. Bitterlich, B.; Anilkumar, G.; Gelalcha, F. G.; Spilker, B.; Grotevendt, A.; Jackstell, R.; Tse, M. K.; Beller, M. Development of a General and Efficient Iron-Catalyzed Epoxidation with Hydrogen Peroxide as Oxidant. *Chem. Asian J*. **2007**, *2*, 521-529.
2. Gelalcha, F. G.; Anilkumar, G.; Tse, M. K.; Bruckner, A.; Beller, M. Biomimetic Iron-Catalyzed Asymmetric Epoxidation of Aromatic Alkenes by Using Hydrogen Peroxide. *Chem. Eur. J*. **2008**, *14*, 7687-7698.
3. Park, H.; Ahn, H. M.; Jeong, H. Y.; Kim, C.; Lee, D. Non-heme iron catalysts for olefin epoxidation: conformationally rigid aryl–aryl junction to support amine/imine multidentate ligands. *Chem. Eur. J.* **2018**, *24*, 8632-8638.
4. Papastergiou, M.; Stathi, P.; Milaeva, E. R., Deligiannakis, Y.; Louloudi, M. Comparative study of the catalytic thermodynamic barriers for two homologous Mn- and Fe-non-heme oxidation catalysts. *J. Catal.* **2016**, *341*, 104-115.
5. Singh, K. K.; Tiwari, M. K.; Dhar, B. B.; Vanka, K.; Sen Gupta, S. Mechanism of oxygen atom transfer from Fe^{V}(O) to olefins at room temperature. *Inorg. Chem.* **2015**, *54*, 6112-6121.
6. Wang, B.; Lee, Y.-M.; Seo, M. S.; Nam, W. Mononuclear Nonheme Iron(III)-Iodosylarene and High-Valent Iron-Oxo Complexes in Olefin Epoxidation Reactions. *Angew. Chem., Int. Ed.* **2015**, *54*, 11740-11744.
7. Shaabani, A.; Mohammadian, R.; Farhid, H.; Karimi Alavijeh, M.; Amini, M. M. Iron-decorated, guanidine functionalized metal-organic framework as a non-heme iron-based enzyme mimic system for catalytic oxidation of organic substrates. *Catal. Lett.* **2019**, *149*, 1237-1249.
8. Perandones, B. F.; del Rio Nieto, E.; Godard, C.; Castillon, S.; De Frutos, P.; Claver, C. Fe-Catalyzed olefin epoxidation with tridentate non-heme ligands and hydrogen peroxide as the oxidant. *ChemCatChem* **2013**, *5*, 1092-1095.
9. Cusso, O.; Garcia-Bosch, I.; Ribas, X.; Lloret-Fillol, J.; Costas, M. Asymmetric Epoxidation with H_2O_2 by Manipulating the Electronic Properties of Non-heme Iron Catalysts. *J. Am. Chem. Soc*. **2013**, *135*, 14871-14878.
10. Iron(III) aroylhydrazone complexes: Structure, electrochemical studies and catalytic activity in oxidation of olefins. *J. Mol. Catal. A – Chem.* **2009**, *304*, 139-146.
11. Moelands, M. A. H.; Schamhart, D. J.; Folkertsma, E.; Lutz, M.; Spek, A. L.; Klein Gebbink, R. J. M. Facial triad modeling using ferrous pyridinyl prolinate complexes: synthesis and catalytic applications. *Dalton Trans.* **2014**, *43*, 6769-6785.

6 ^{1}H NMR and ^{13}C NMR spectra of new ligands

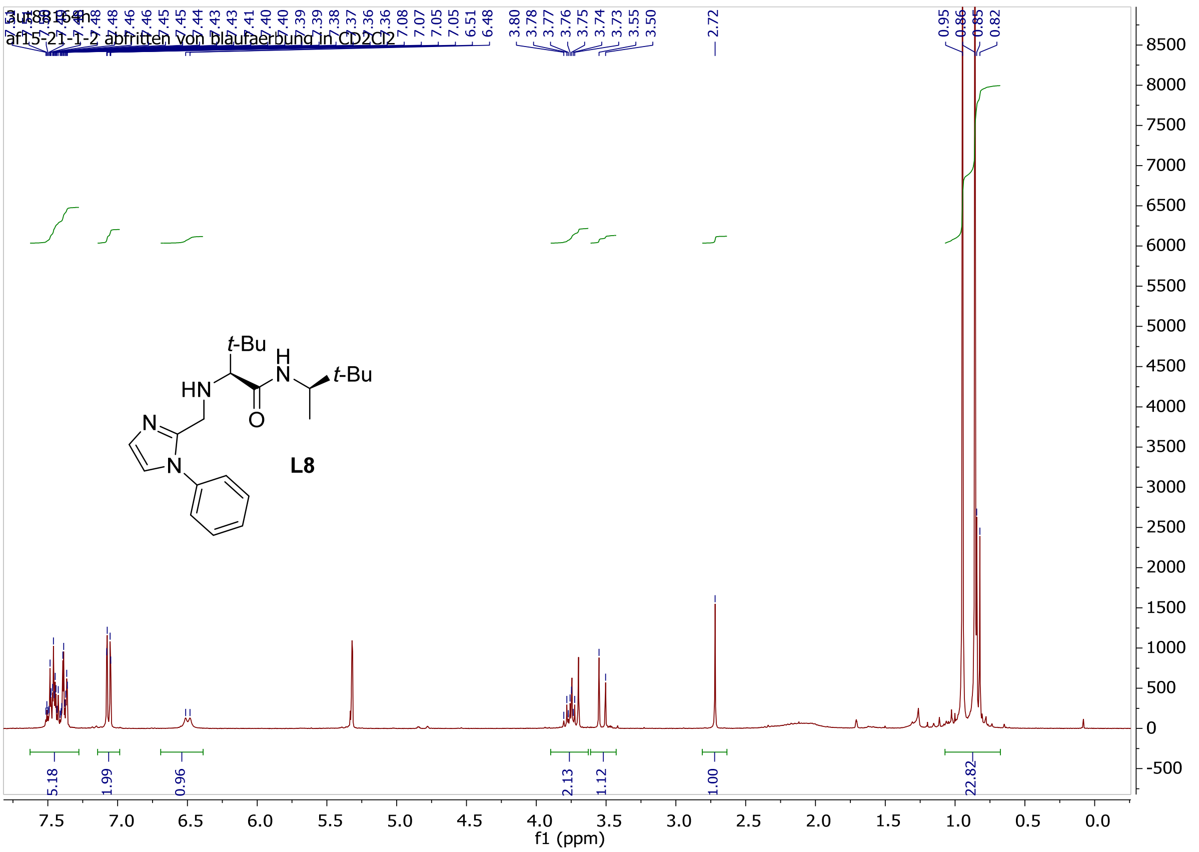

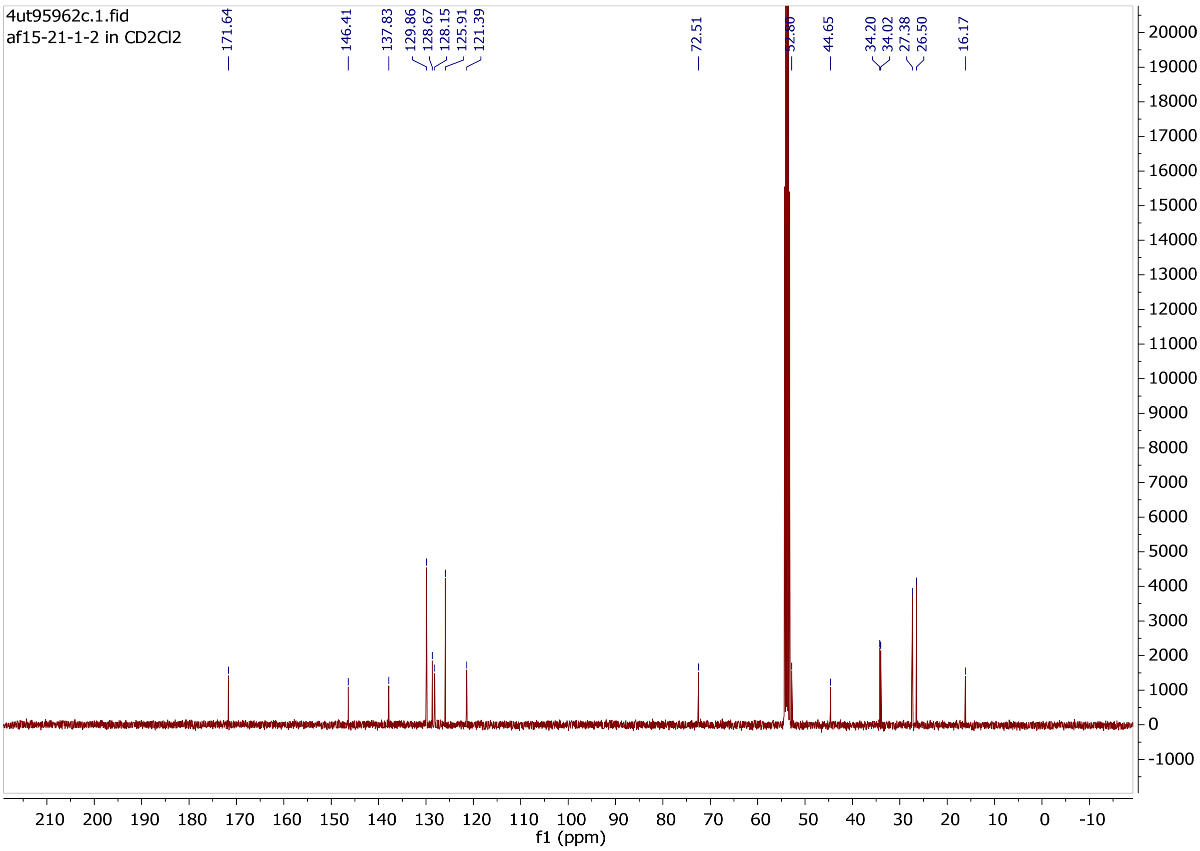

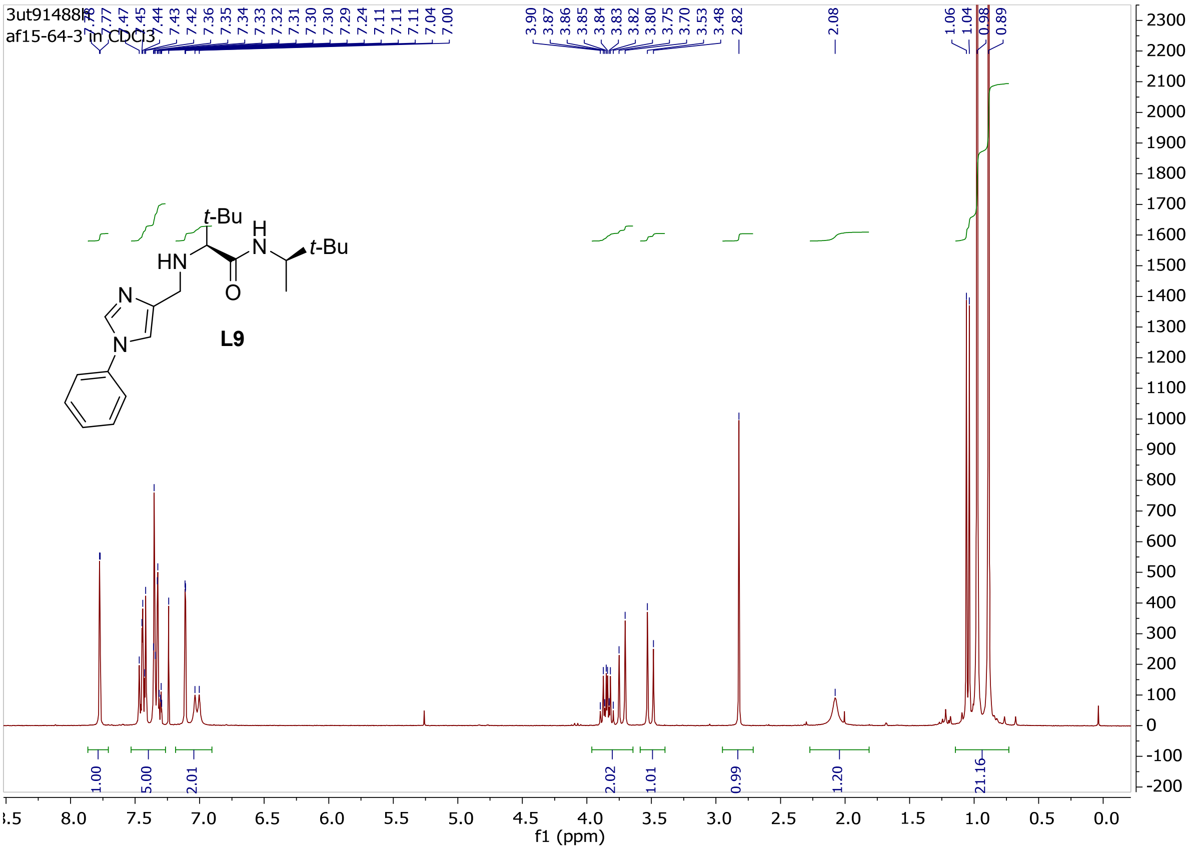
3ut91488H
af15-64-3 in CDCl3
t-Bu
HN
N
N
H
N
t-Bu
O
L9
f1 (ppm)

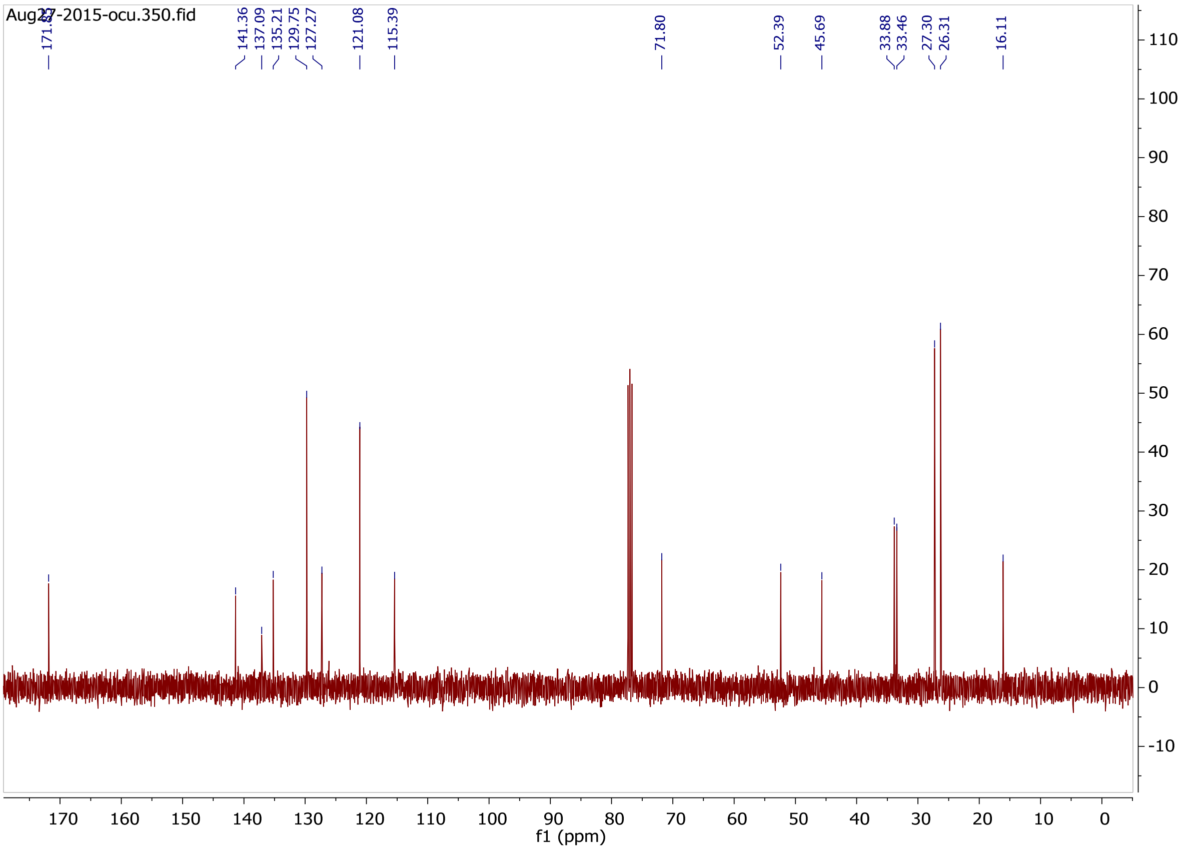
Aug27-2015-ocu.350.fid
f1 (ppm)

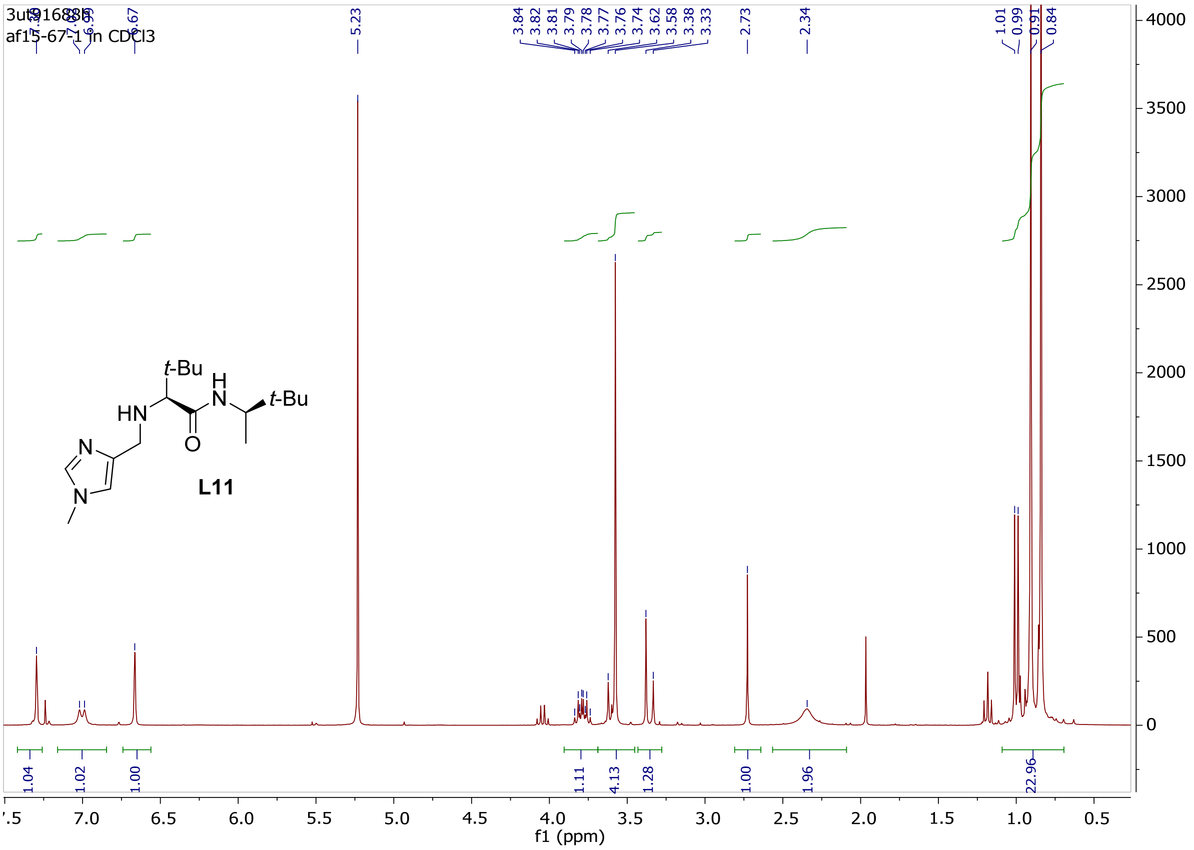
3ut916886
af15-67-1 in CDCl3
t-Bu
HN
H
N
t-Bu
O
N
N
L11
f1 (ppm)

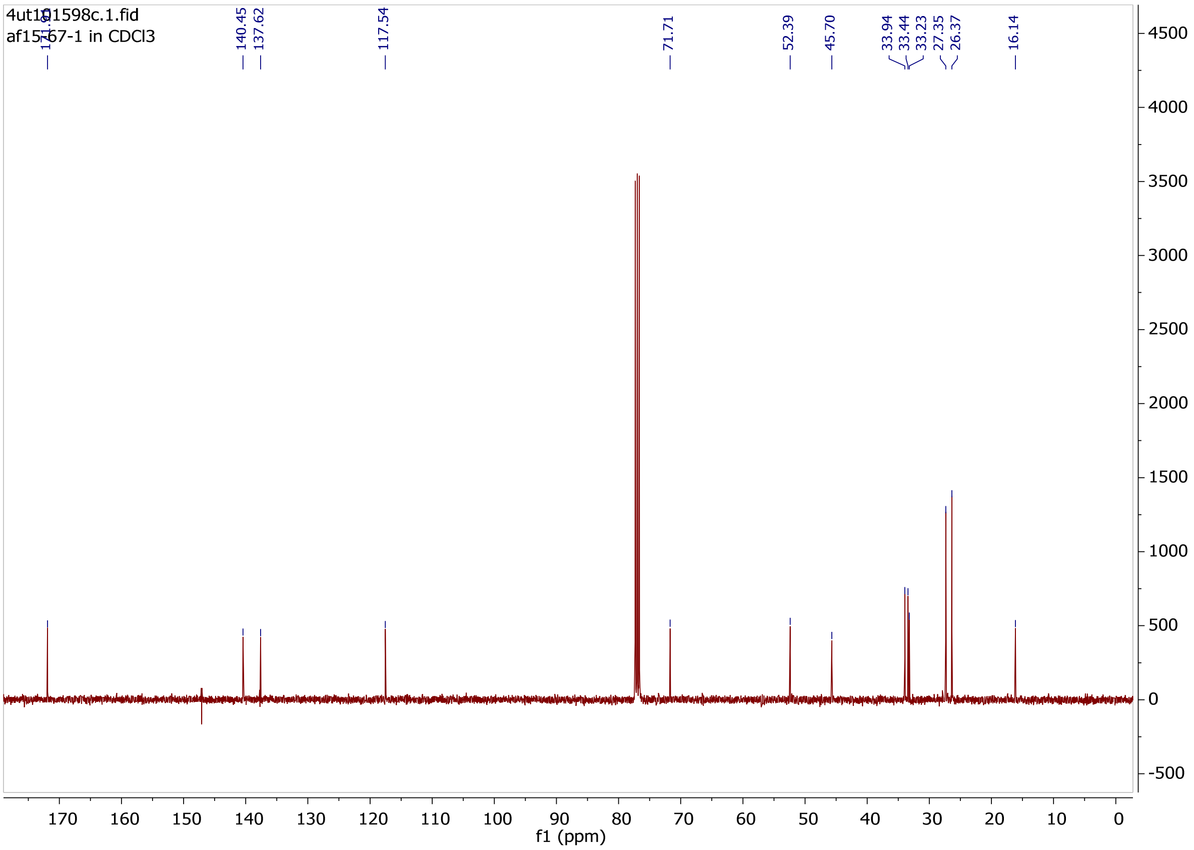
4ut101598c.1.fid
af15-67-1 in CDCl3
140.45
137.62
117.54
71.71
52.39
45.70
33.94
33.44
33.23
27.35
26.37
16.14
f1 (ppm)

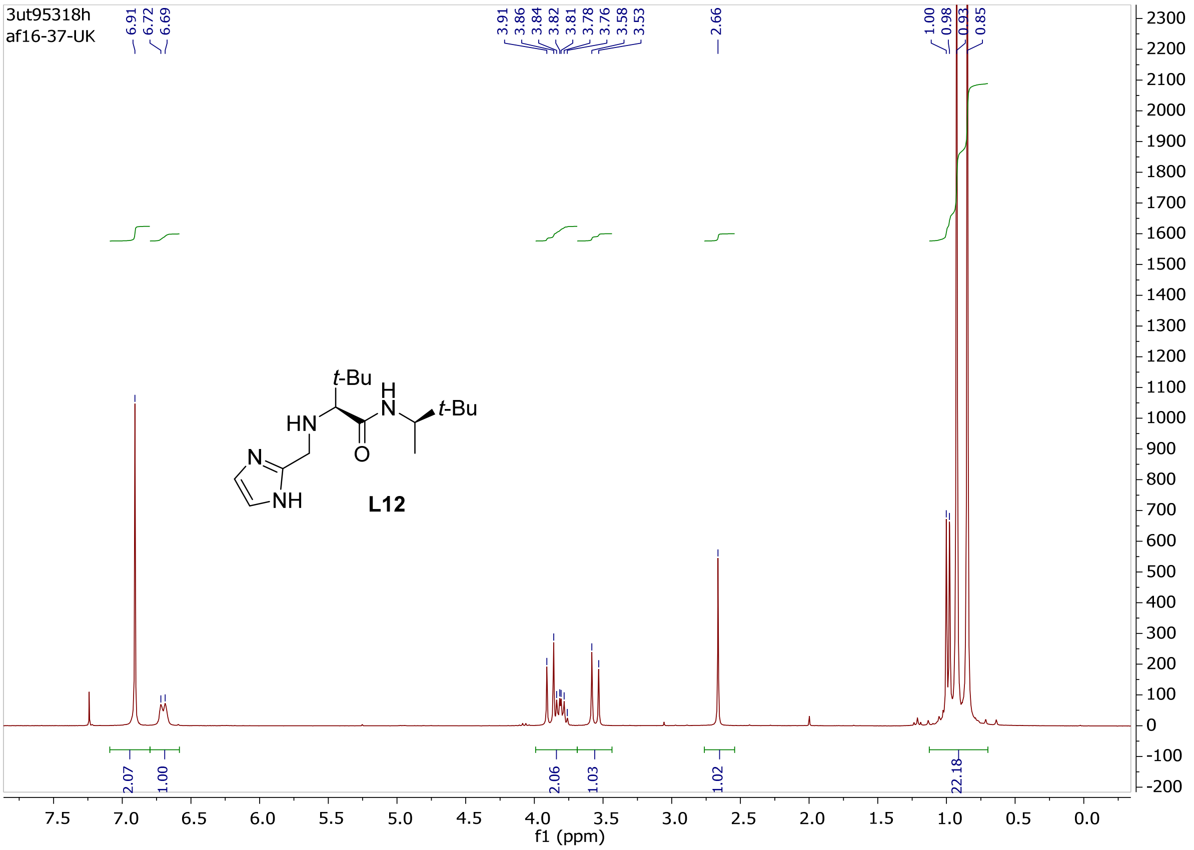
3ut95318h
af16-37-UK
6.91
6.72
6.69
3.91
3.86
3.84
3.82
3.81
3.78
3.76
3.58
3.53
2.66
1.00
0.98
0.93
0.85
t-Bu
HN
H
N
t-Bu
O
N
NH
L12
2.07
1.00
2.06
1.03
1.02
22.18
f1 (ppm)

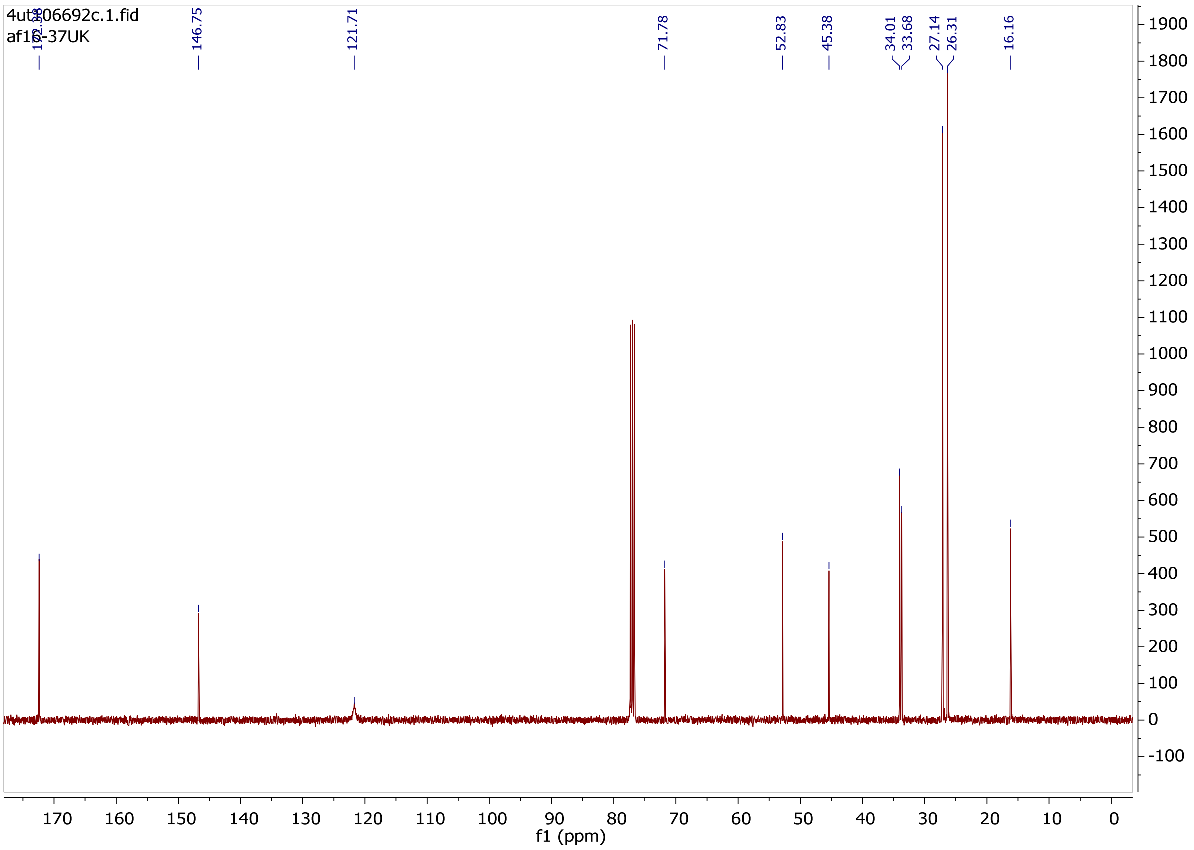
146.75
121.71
71.78
52.83
45.38
34.01
33.68
27.14
26.31
16.16
f1 (ppm)

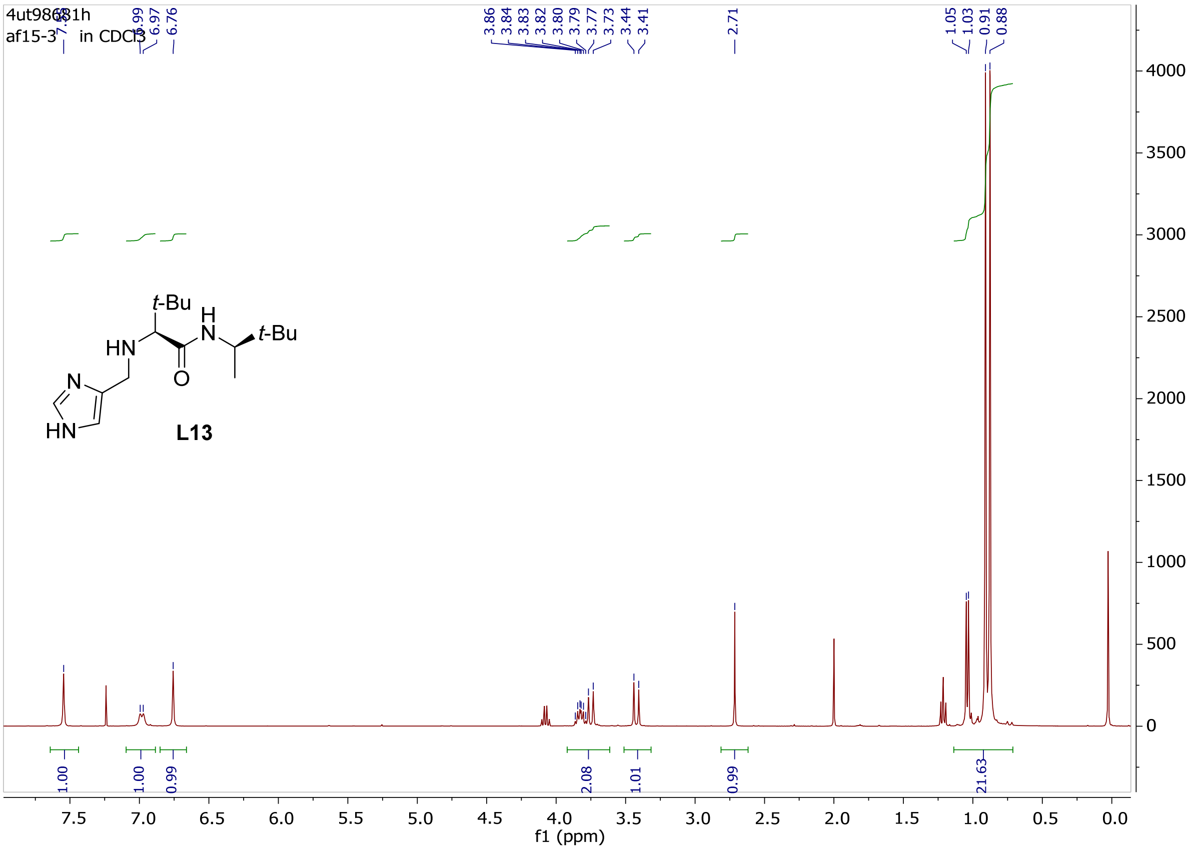
4ut98681h
af15-3 in CDCl3
t-Bu
HN
H
N
t-Bu
O
N
HN
L13
f1 (ppm)

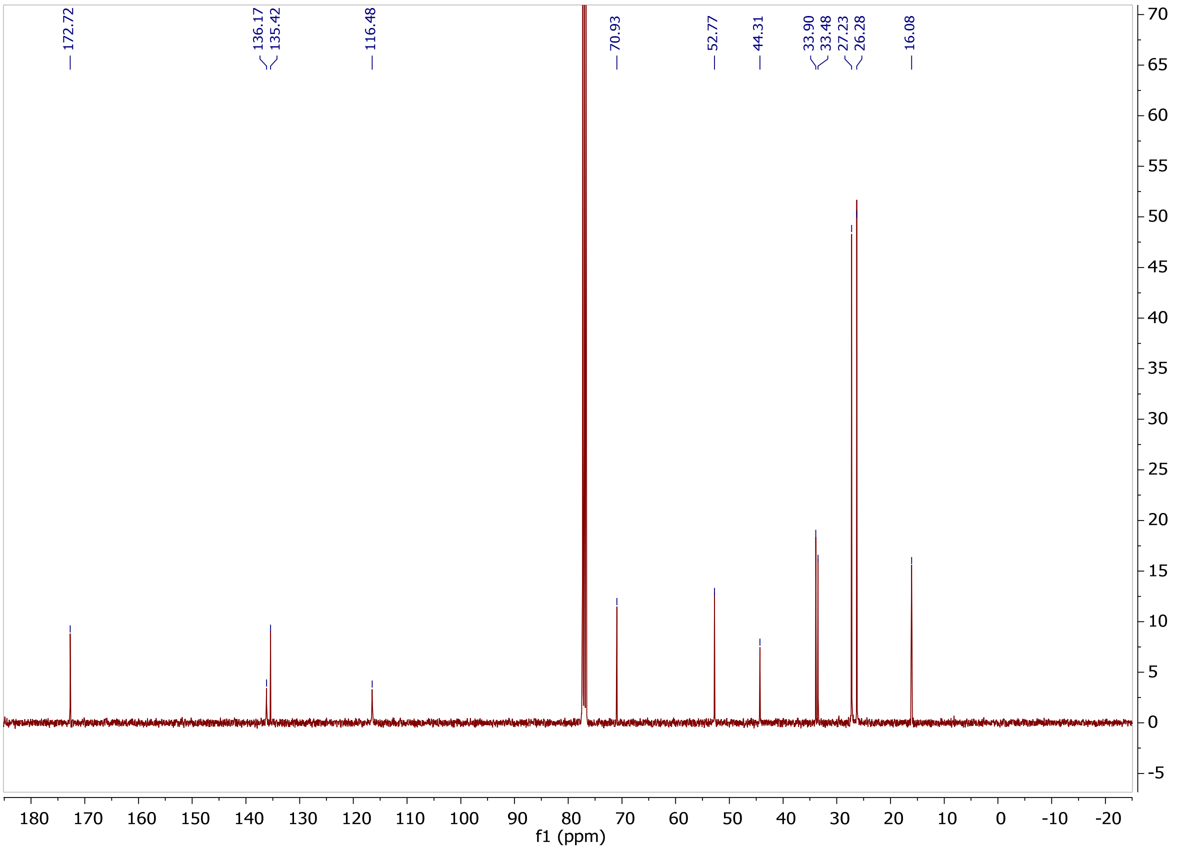
172.72
136.17
135.42
116.48
70.93
52.77
44.31
33.90
33.48
27.23
26.28
16.08
f1 (ppm)

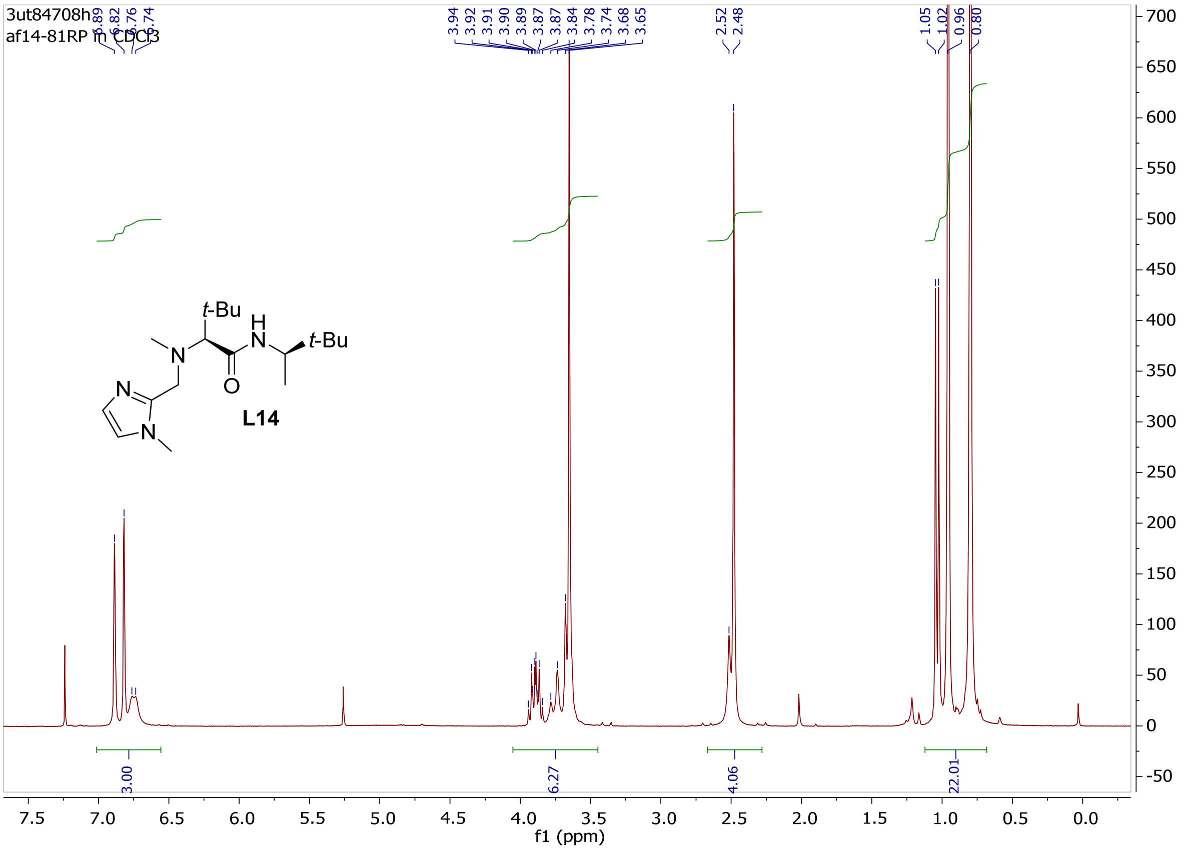
3ut84708h
af14-81RP in CDCl3
6.89 6.82 6.76 6.74
3.94 3.92 3.91 3.90 3.89 3.87 3.87 3.84 3.78 3.74 3.68 3.65
2.52 2.48
1.05 1.02 0.96 0.80
t-Bu
H
N
t-Bu
N
O
N
N
L14
3.00
6.27
4.06
22.01
f1 (ppm)

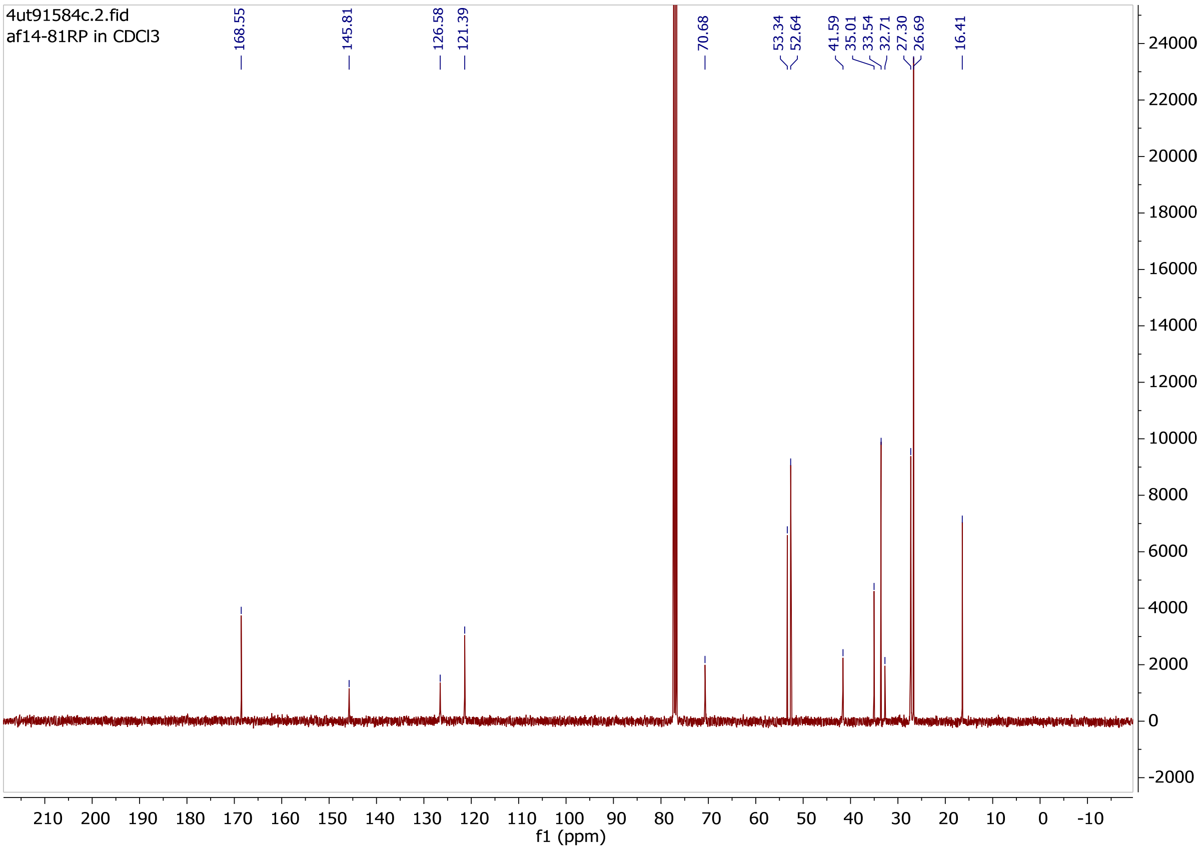
4ut91584c.2.fid
af14-81RP in CDCl3
168.55
145.81
126.58
121.39
70.68
53.34 52.64
41.59 35.01 33.54 32.71 27.30 26.69
16.41
f1 (ppm)

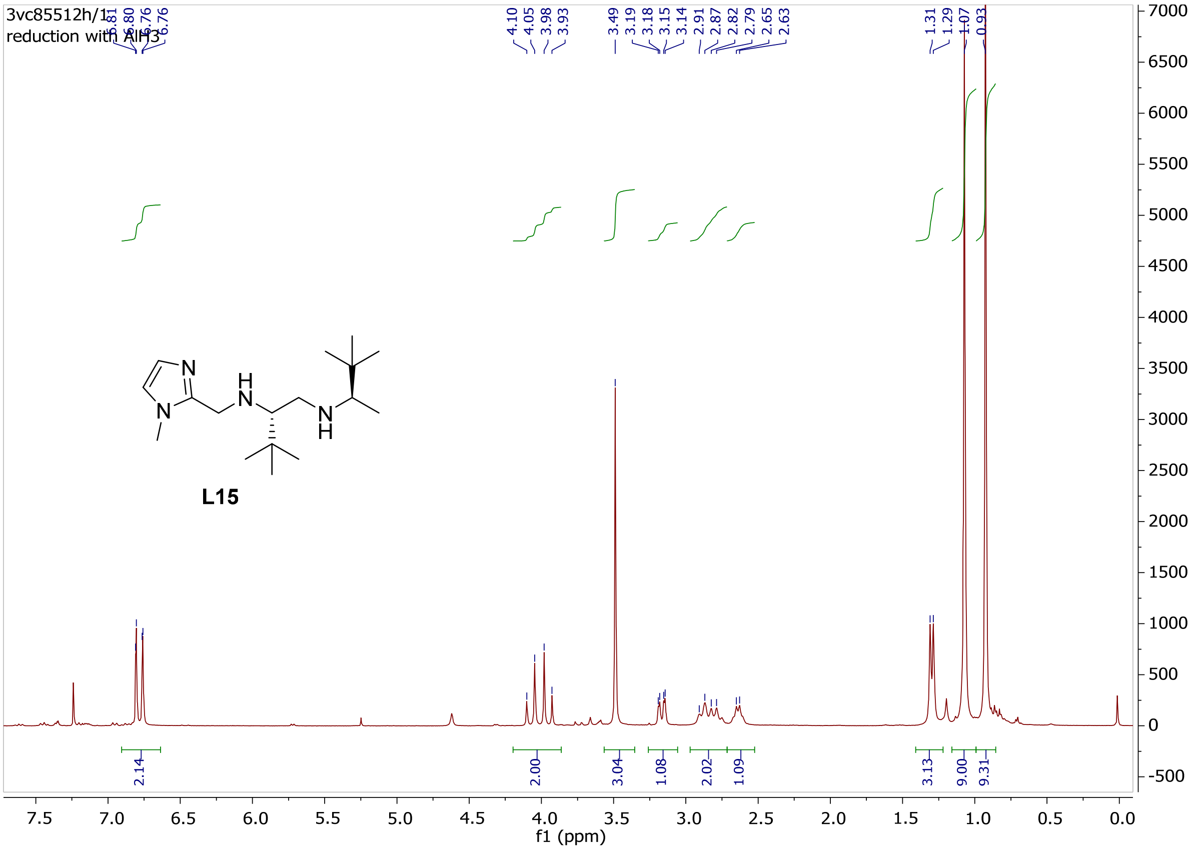
3vc85512h/1
reduction with AlH3
6.80
6.76
6.76
4.10
4.05
3.98
3.93
3.49
3.19
3.18
3.15
3.14
2.91
2.87
2.82
2.79
2.65
2.63
1.31
1.29
1.07
0.93
L15
2.14
2.00
3.04
1.08
2.02
1.09
3.13
9.00
9.31
f1 (ppm)

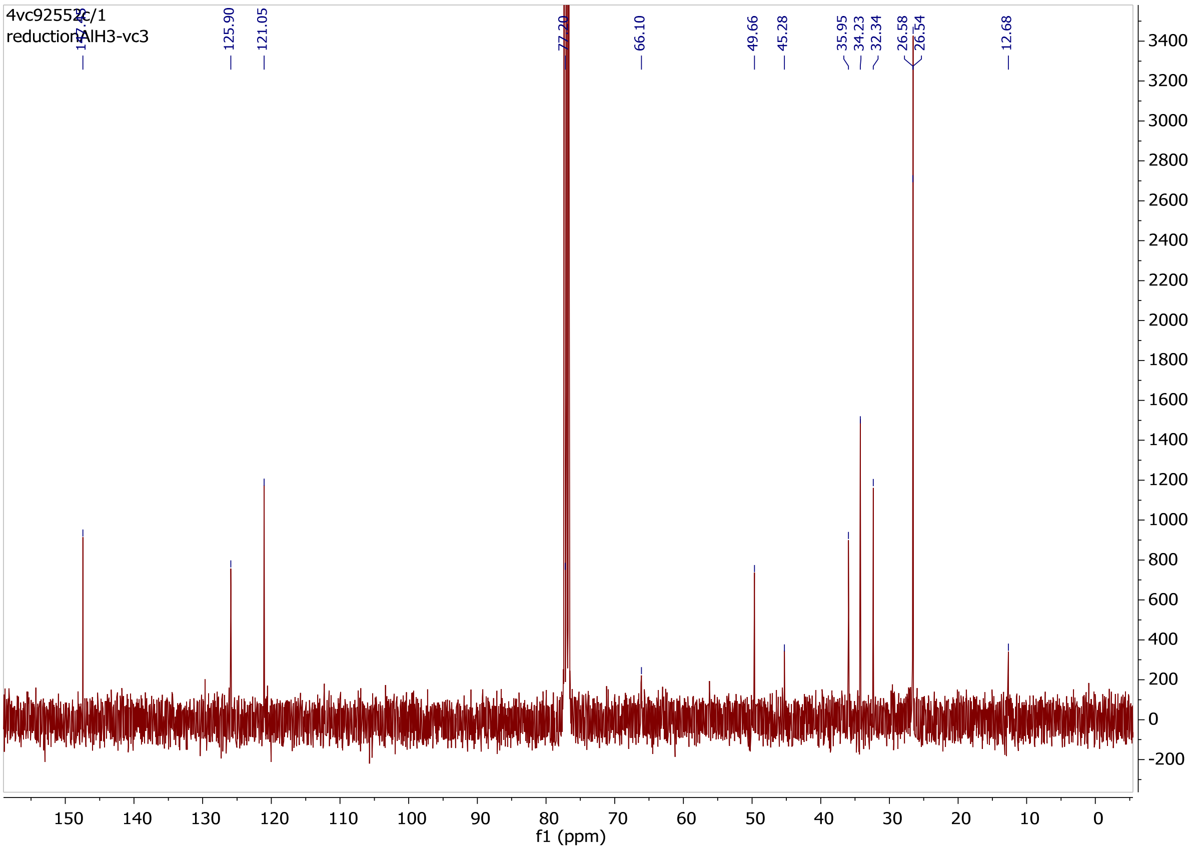
4vc92552c/1
reduction AlH3-vc3
125.90
121.05
66.10
49.66
45.28
35.95
34.23
32.34
26.58
26.54
12.68
f1 (ppm)

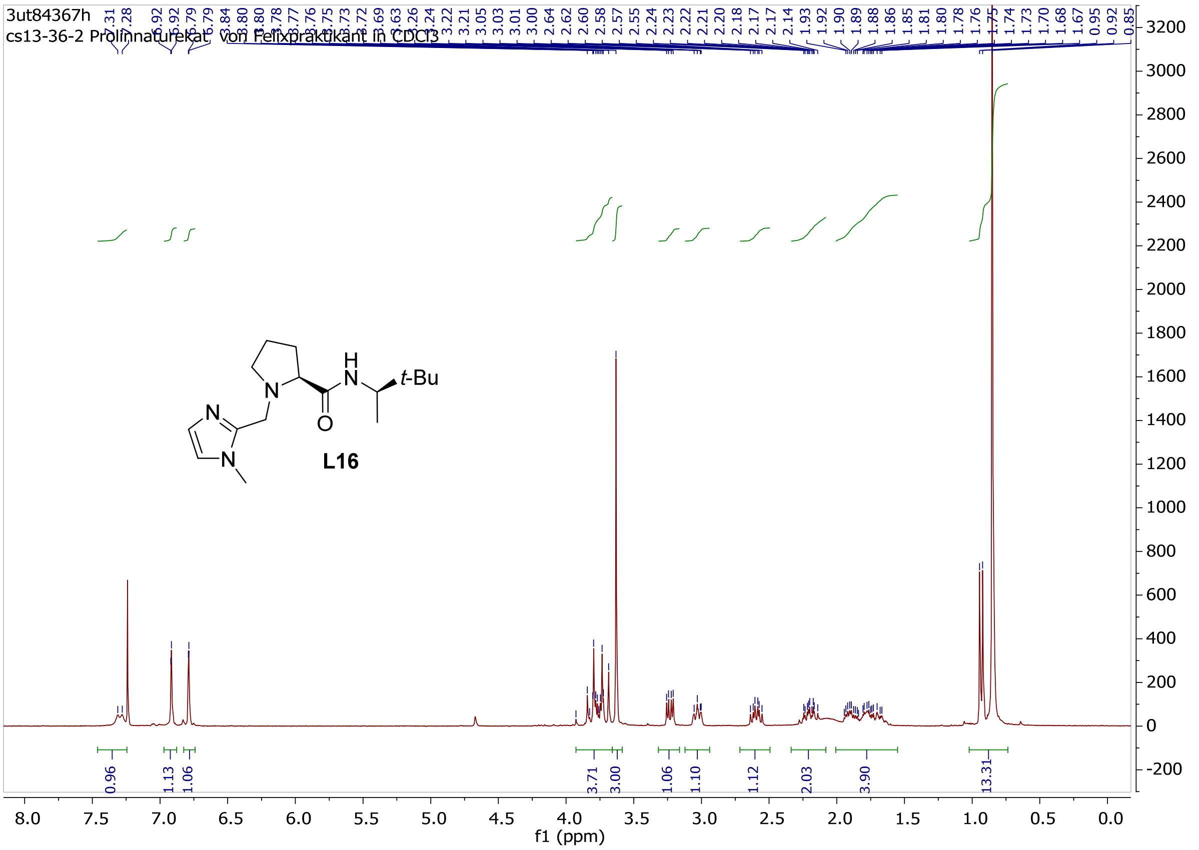
3ut84367h
cs13-36-2 Prolinnaturekat. von Felixpraktikant in CDCl3
7.31 7.28 6.92 6.92 6.79 6.79 3.84 3.80 3.80 3.78 3.77 3.76 3.75 3.73 3.72 3.69 3.63 3.26 3.24 3.22 3.21 3.05 3.03 3.01 3.00 2.64 2.62 2.60 2.58 2.57 2.55 2.24 2.23 2.22 2.21 2.20 2.18 2.17 2.17 2.14 1.93 1.92 1.90 1.89 1.88 1.86 1.85 1.81 1.80 1.78 1.76 1.75 1.74 1.73 1.70 1.68 1.67 0.95 0.92 0.85
0.96 1.13 1.06 3.71 3.00 1.06 1.10 1.12 2.03 3.90 13.31
H
N
t-Bu
N
O
N
N
L16
f1 (ppm)

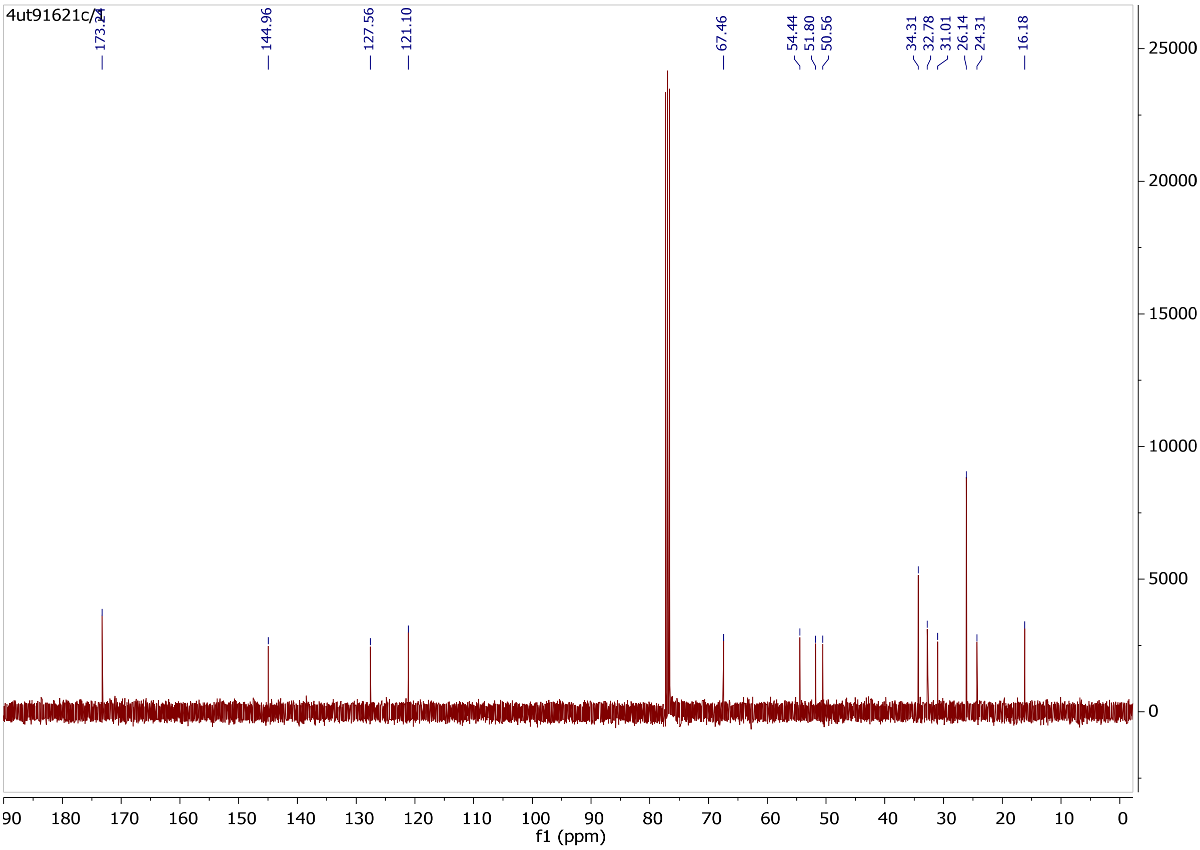
4ut91621c/1
144.96 127.56 121.10 67.46 54.44 51.80 50.56 34.31 32.78 31.01 26.14 24.31 16.18
f1 (ppm)

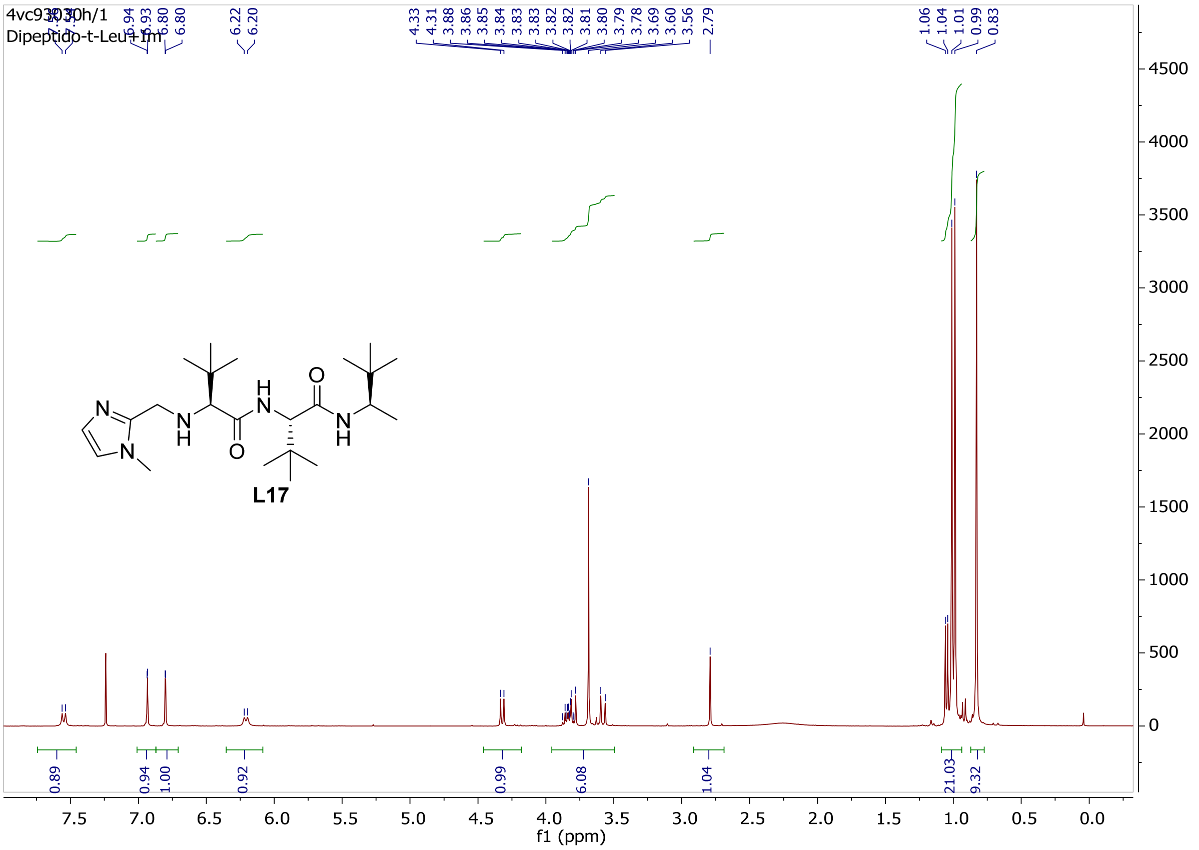
4vc93030h/1
Dipeptido-t-Leu+Im
6.94
6.93
6.80
6.80
6.22
6.20
4.33
4.31
3.88
3.86
3.85
3.84
3.83
3.83
3.82
3.82
3.81
3.80
3.79
3.78
3.69
3.60
3.56
2.79
1.06
1.04
1.01
0.99
0.83
L17
0.89
0.94
1.00
0.92
0.99
6.08
1.04
21.03
9.32
f1 (ppm)

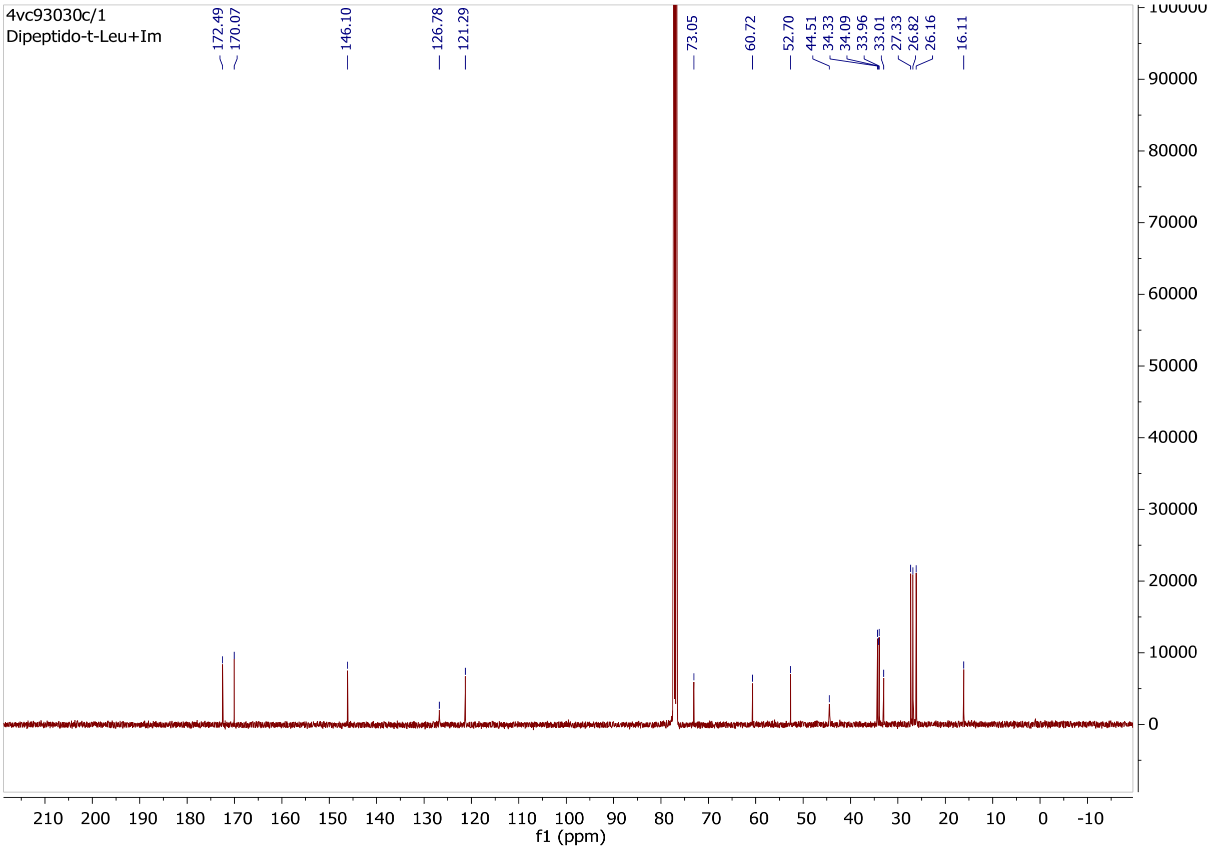
4vc93030c/1
Dipeptido-t-Leu+Im
172.49
170.07
146.10
126.78
121.29
73.05
60.72
52.70
44.51
34.33
34.09
33.96
33.01
27.33
26.82
26.16
16.11
f1 (ppm)

7 HPLC chromatograms

2-Naphthalen-2-yl-oxirane

Chiral HPLC (Daicel Chiralpak AS): *n*-hexane/*i*-PrOH 99:1, 0.5 ml/min flow rate, λ = 254 nm;

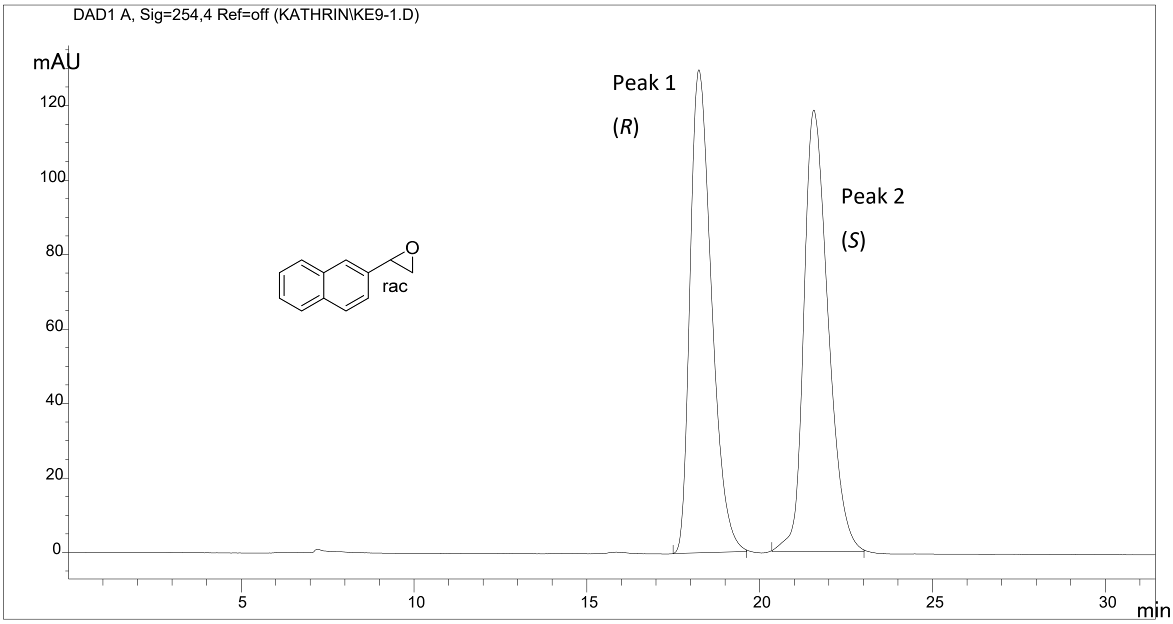

Peak	t_R	Area	Area (%)
1	18.236	5621.8	49.24
2	21.563	5796	50.76

See Entry 1, Table 2

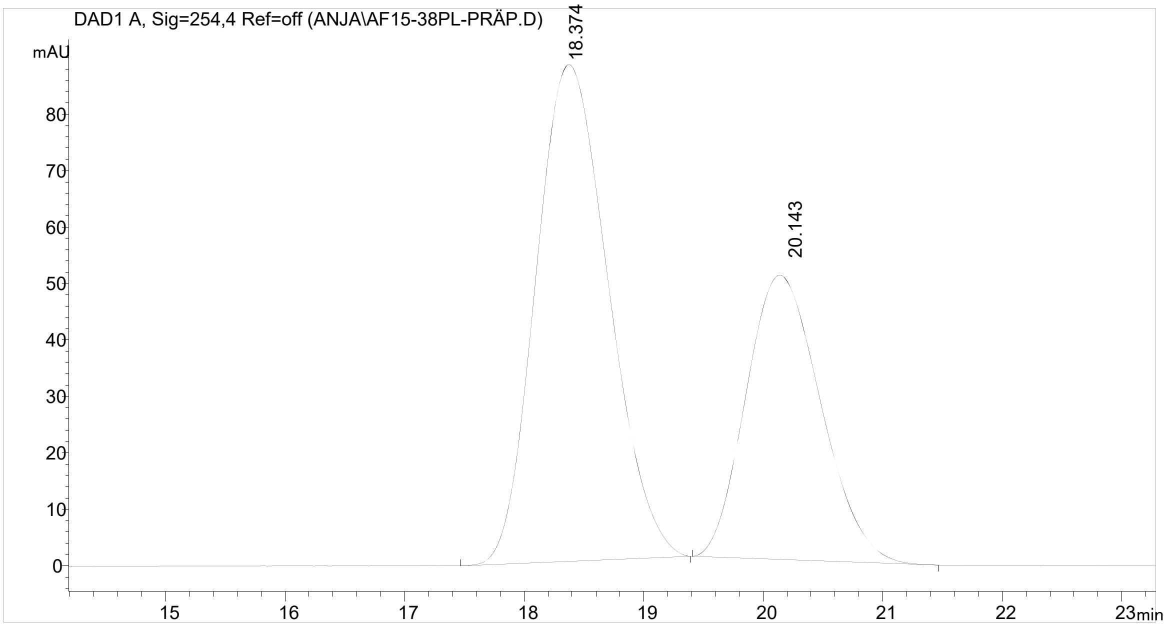

Peak	t_R	Area	Area (%)
1	18.374	3715.3	62.867
2	20.143	2194.5	37.133

2-Phenyloxirane

Chiral HPLC (Macherey-Nagel OD): *n*-hexane/*i*-PrOH 99:1, 1.0 ml/min flow rate, λ = 210 nm;

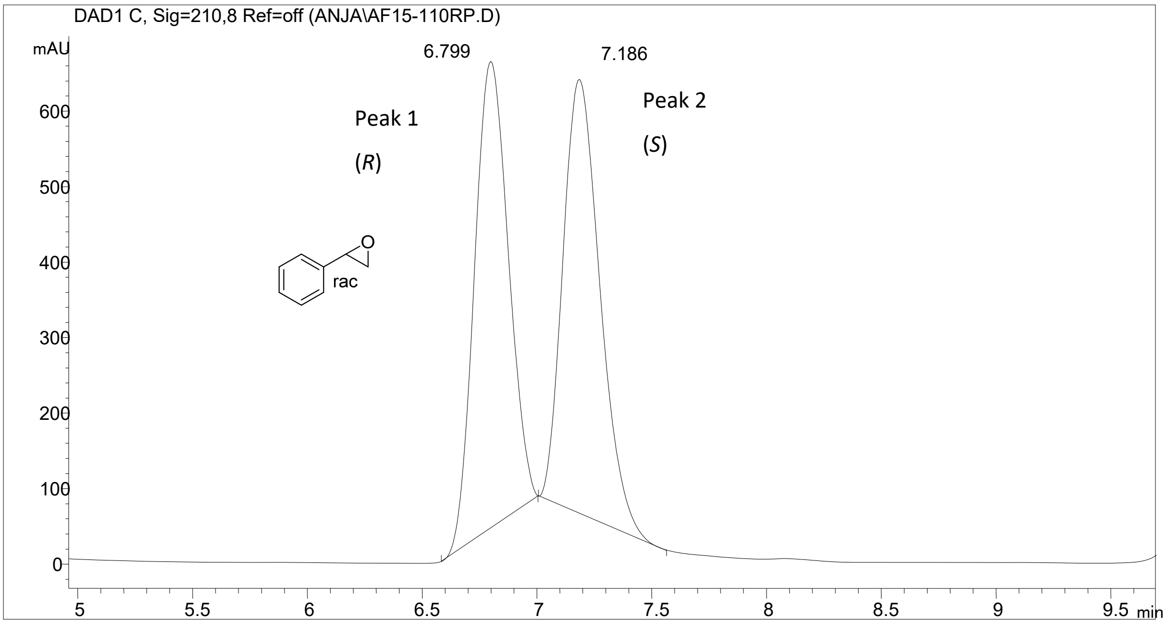

Peak	t_R	Area	Area (%)
1	6.799	6357.5	49.627
2	7.186	6453	50.373

See Entry 2, Table 2

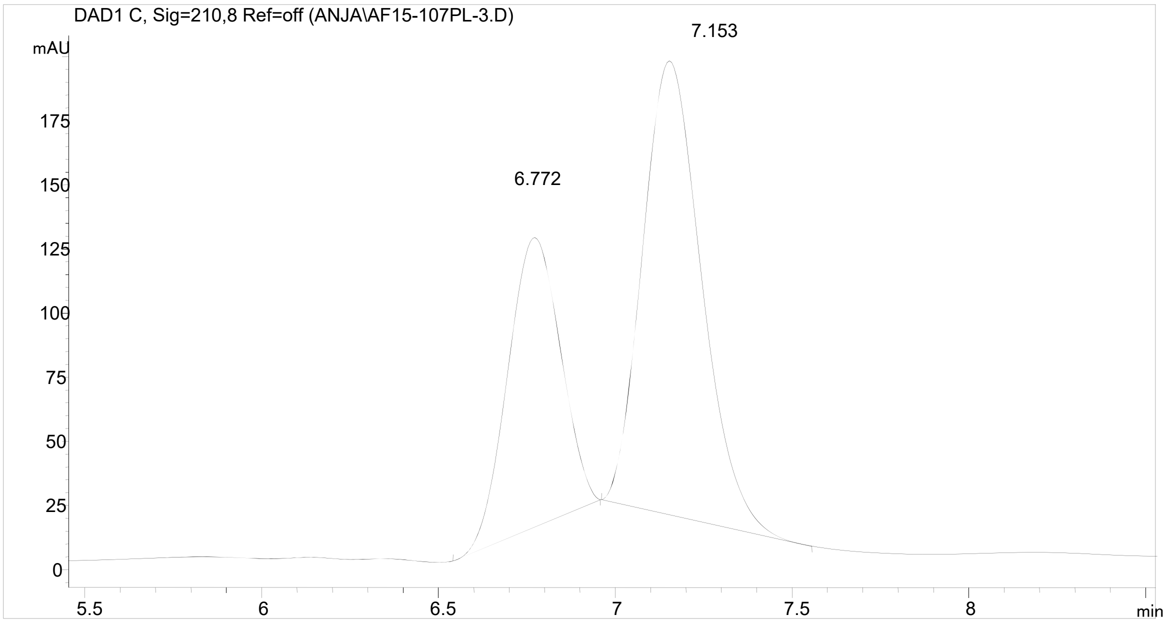

Peak	t_R	Area	Area (%)
1	6.772	1174.8	36.338
2	7.153	2058.1	63.662

2-(4-Nitro-phenyl)-oxirane

Chiral HPLC (Daicel Chiralpak IC): *n*-hexane/*i*-PrOH 95:5, 1.0 ml/min flow rate, λ = 254 nm;

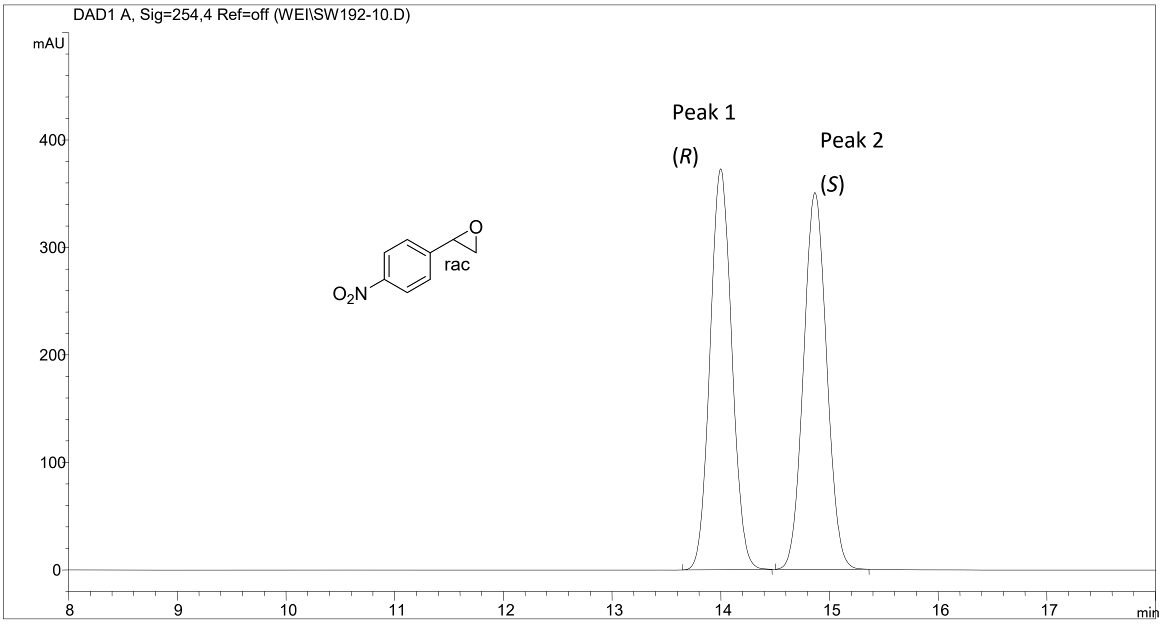

Peak	t_R	Area	Area (%)
1	13.998	360.44	49.94
2	14.865	339.40	50.06

See Entry 3, Table 2 (different retention behaviour due to pressure problems in HPLC system)

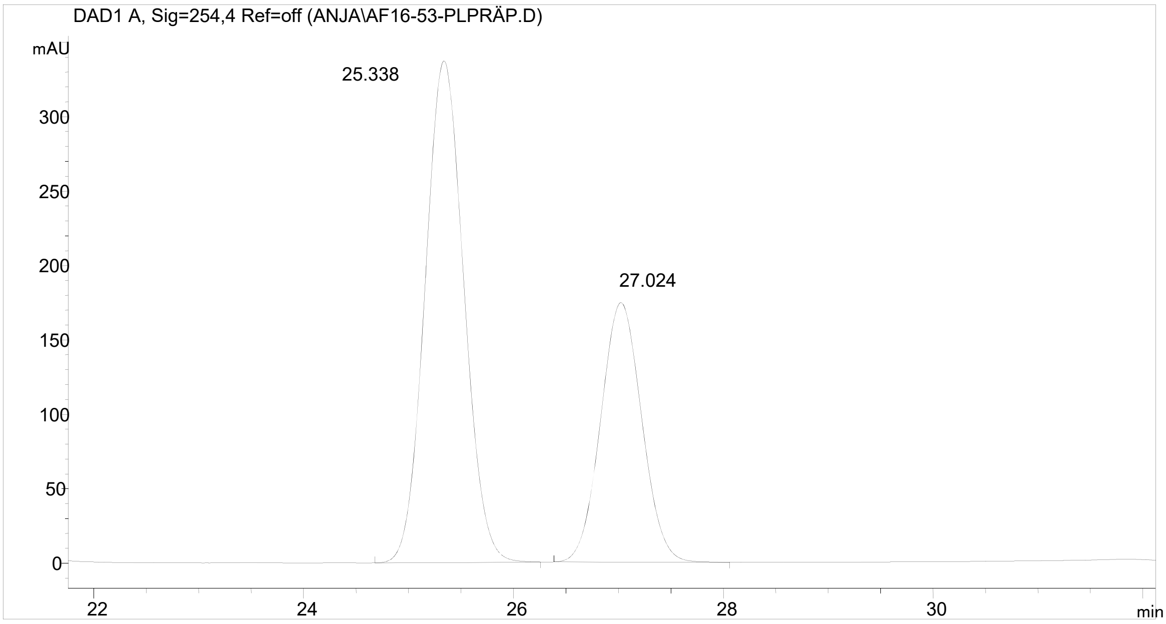

Peak	t_R	Area	Area (%)
1	25.338	8679.2	64.625
2	27.024	4750.9	35.375

2-Methyl-2-phenyloxirane

Chiral HPLC (Macherey-Nagel OD): *n*-hexane/*i*-PrOH 99:1, 1.0 ml/min flow rate, λ = 210 nm;

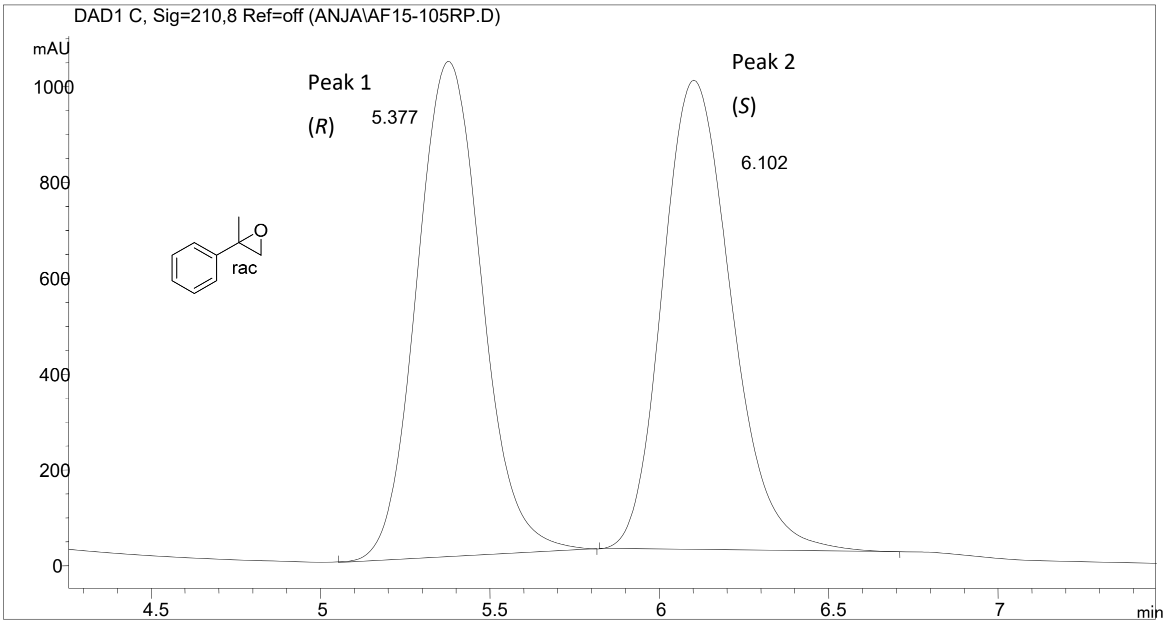

Peak	t_R	Area	Area (%)
1	5.377	13850.2	50.000
2	6.102	13850	50.000

See Entry 6, Table 2

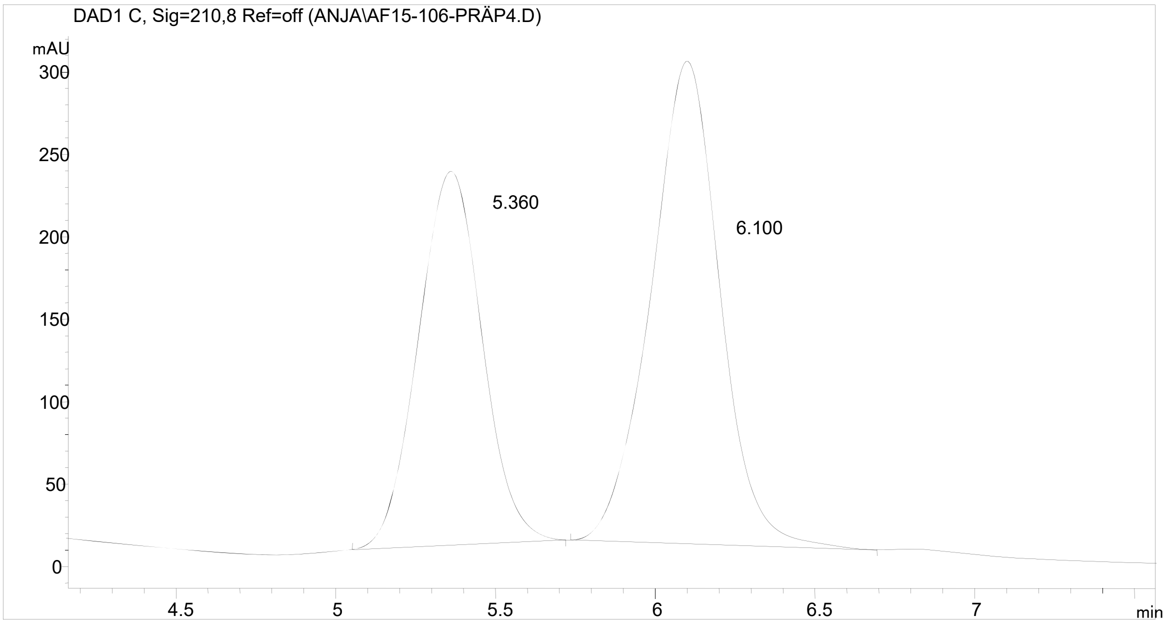

Peak	t_R	Area	Area (%)
1	5.36	3153	41.812
2	6.1	4387.9	58.188

2-Methyl-3-phenyloxirane

Chiral HPLC (Daicel Chiralpak AS): *n*-hexane/*i*-PrOH 99:1, 0.5 ml/min flow rate, λ = 210 nm;

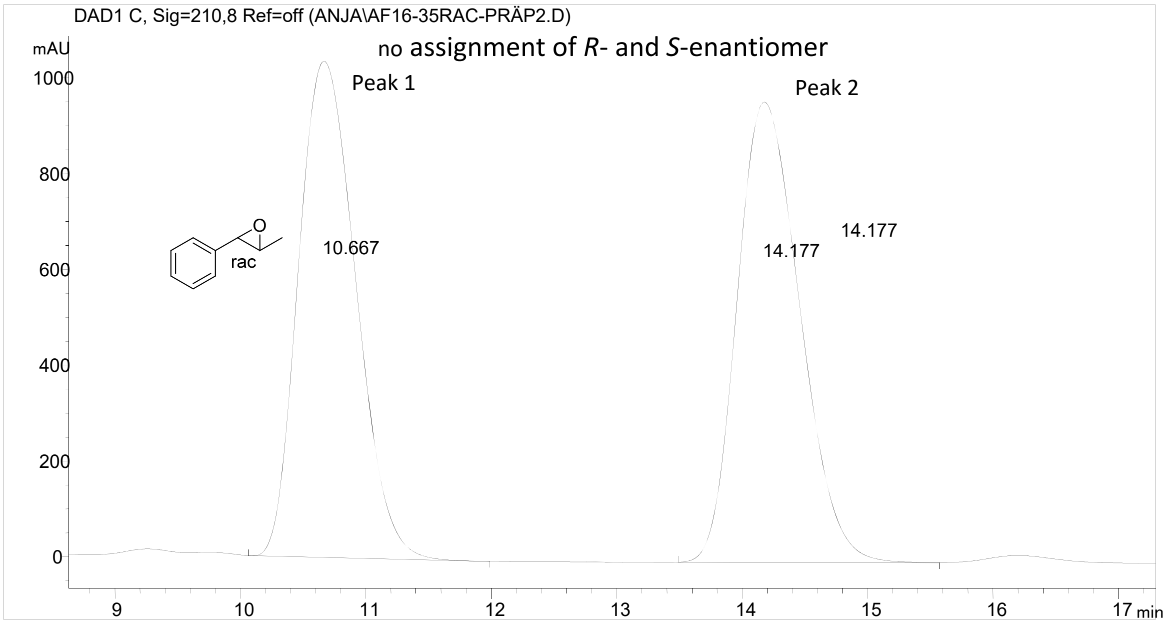

Peak	t_R	Area	Area (%)
1	10.667	32920.4	49.569
2	14.177	33493	50.431

See Entry 8, Table 2

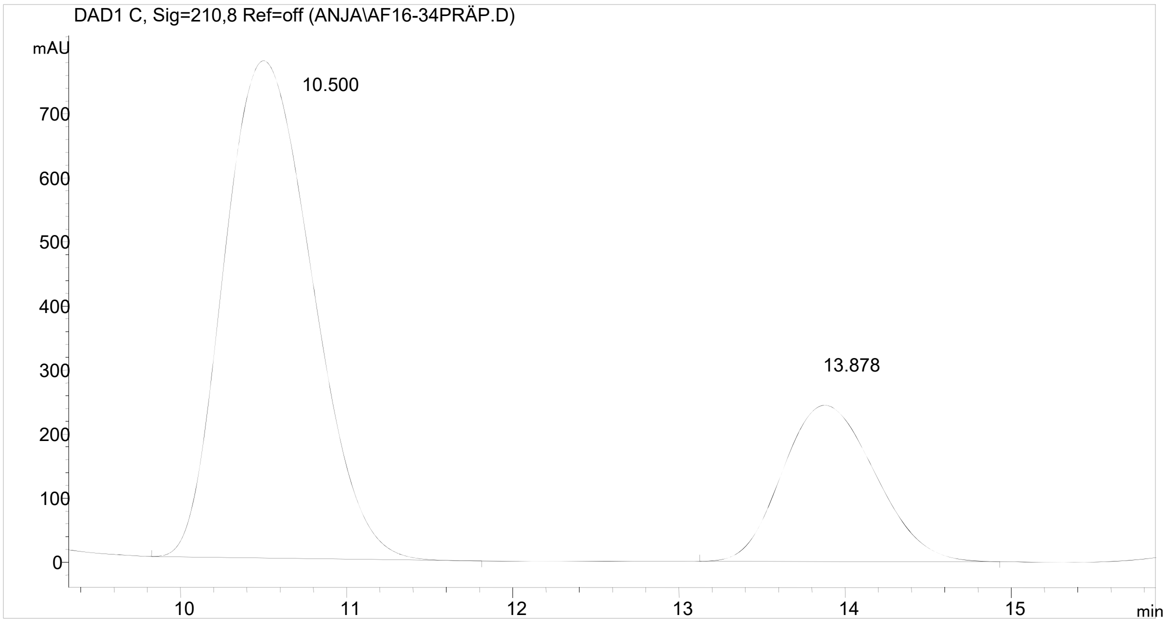

Peak	t_R	Area	Area (%)
1	10.5	28623.3	75.183
2	13.878	9448.3	24.817

2,3-Diphenyloxirane

Chiral HPLC (Daicel Chiralpak IB): *n*-hexane/*i*-PrOH 92:8, 1.0 ml/min flow rate, λ = 210 nm;

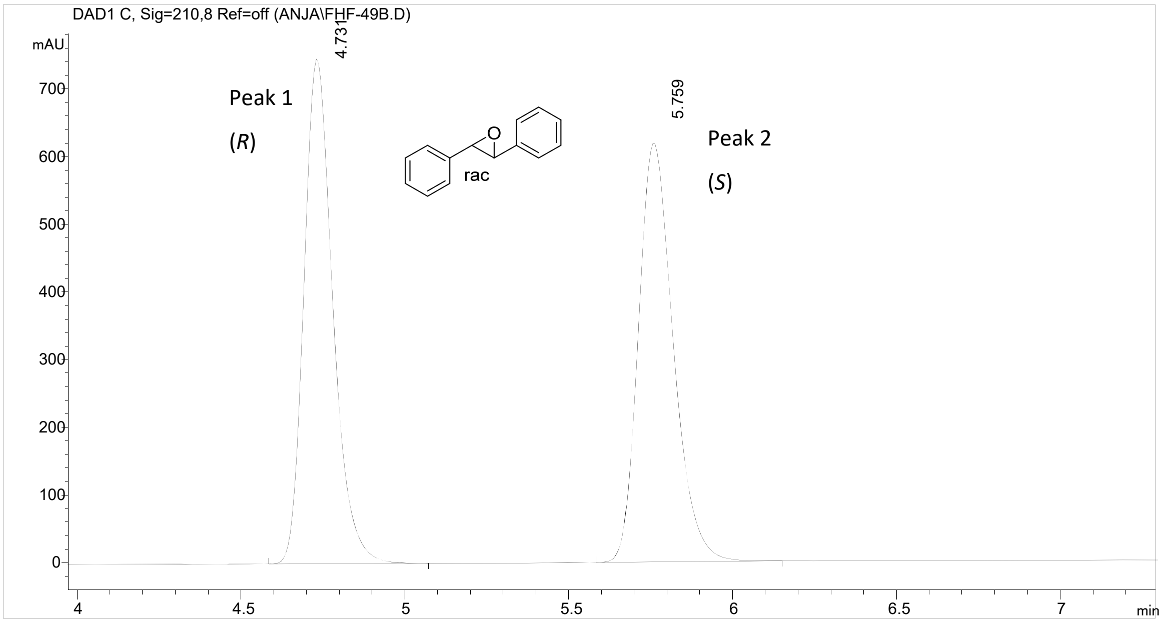

Peak	t_R	Area	Area (%)
1	4.731	4576.1	49.973
2	5.759	4581.1	50.027

See Entry 9, Table 2

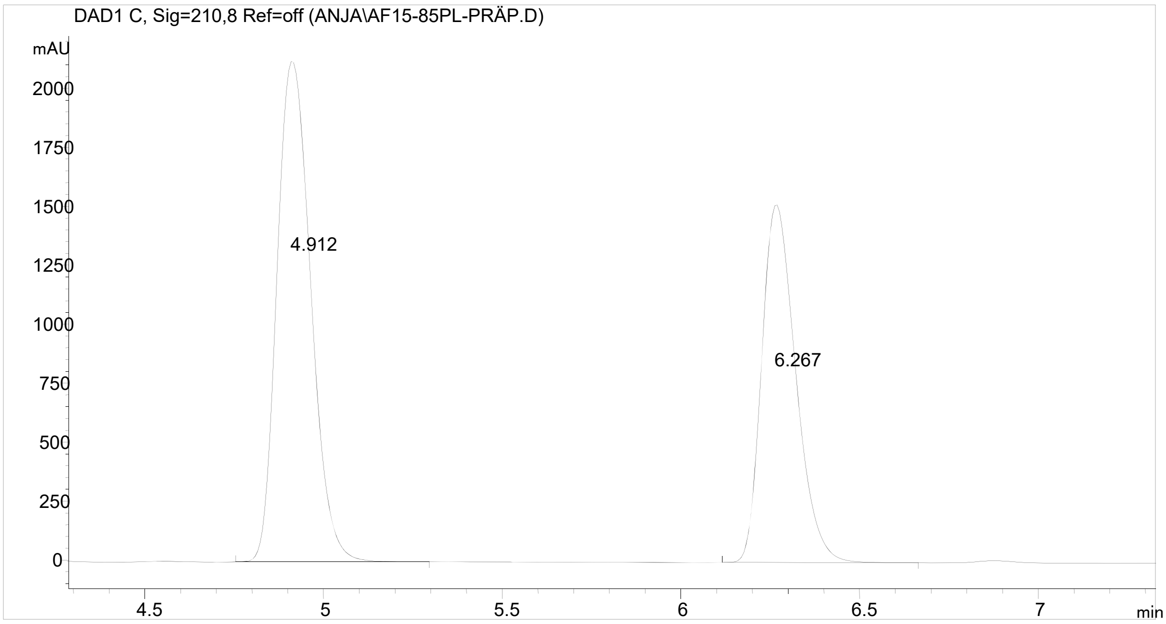

Peak	t_R	Area	Area (%)
1	4.912	13954.8	58.083
2	6.267	10070.9	41.917

Phenyl(3-phenyloxiran-2-yl)methanone

Chiral HPLC (Macherey-Nagel OD): *n*-hexane/*i*-PrOH 90:10, 1.0 ml/min flow rate, λ = 245 nm;

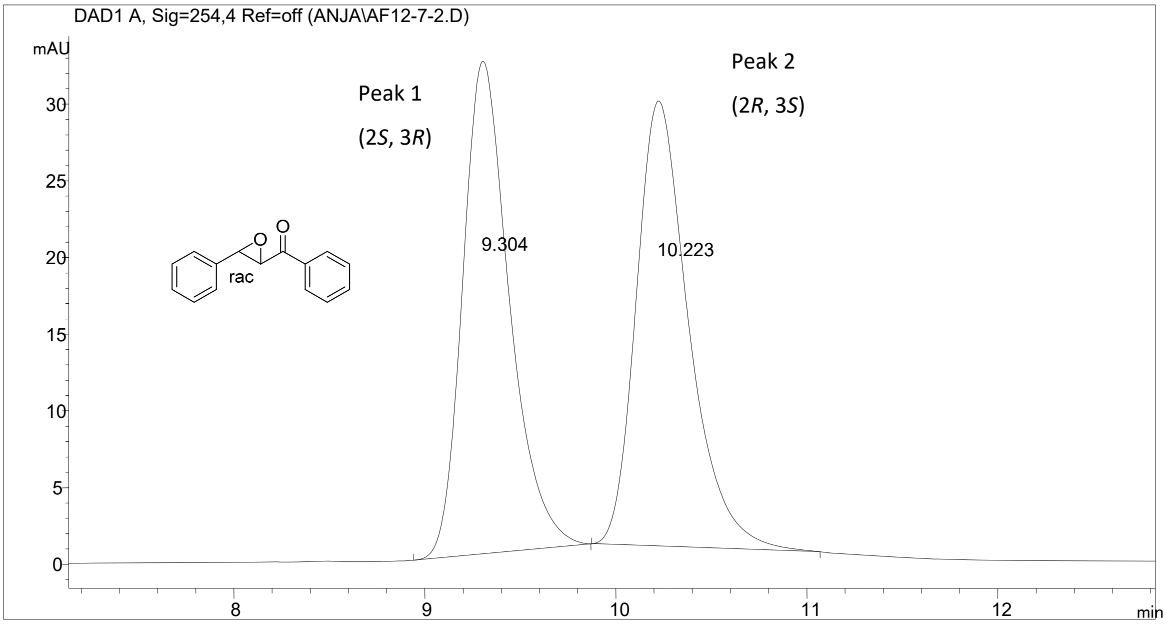

Peak	t_R	Area	Area (%)
1	9.304	547.6	49.702
2	10.223	554.1	50.298

See Entry 10, Table 2

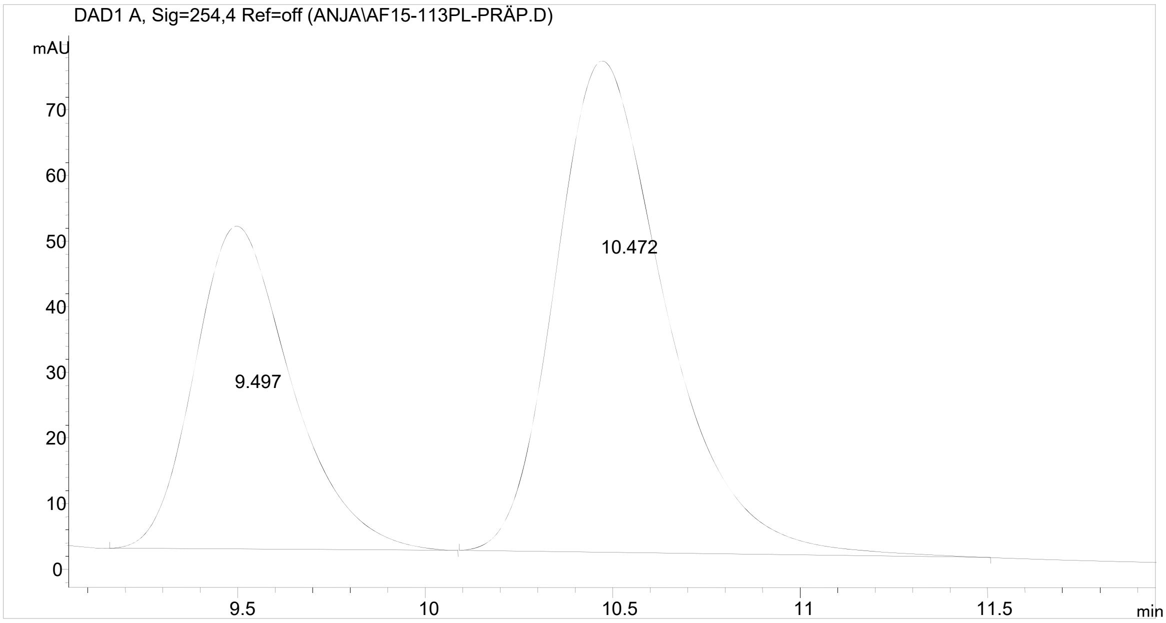

Peak	t_R	Area	Area (%)
1	9.497	884.6	36.474
2	10.472	1540.7	63.526

See Entry 11, Table 2

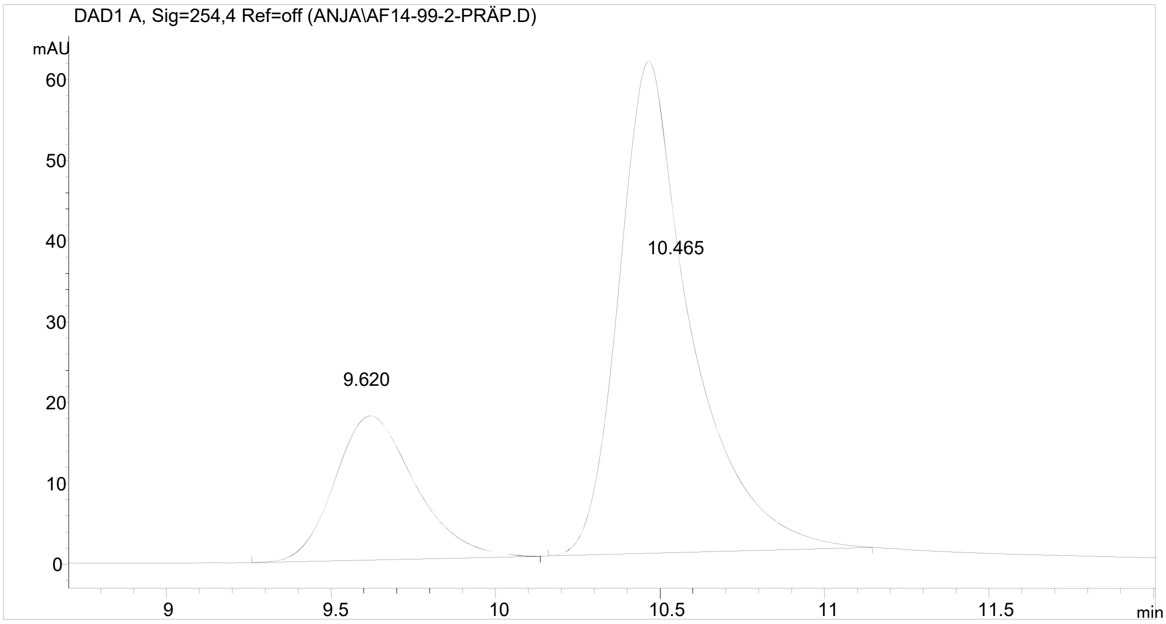

Peak	t_R	Area	Area (%)
1	9.62	304.5	24.589
2	10.465	934	75.411

8 Investigation of Fe(III) complex via UV-Vis spectroscopy

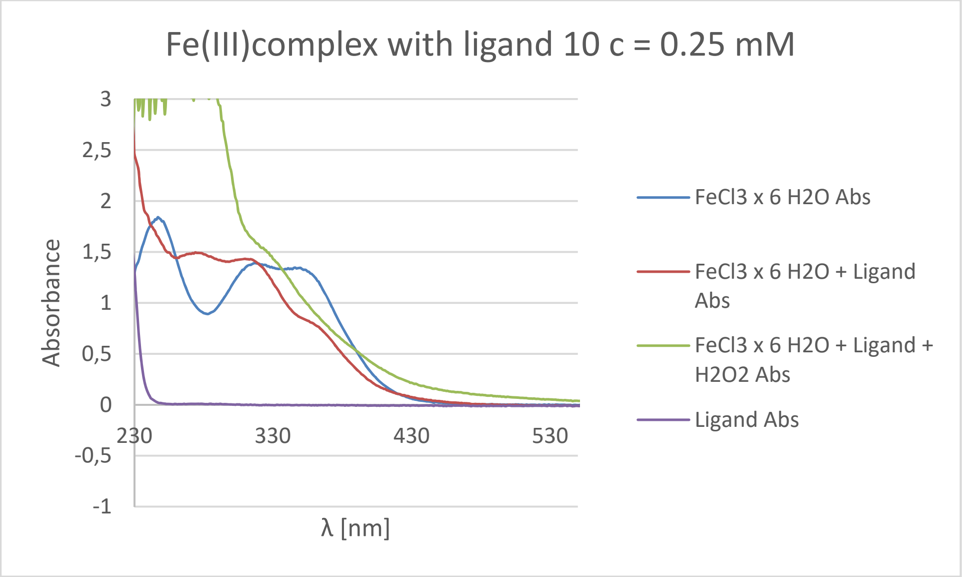

9 Infrared Experiments of Fe(III) complex

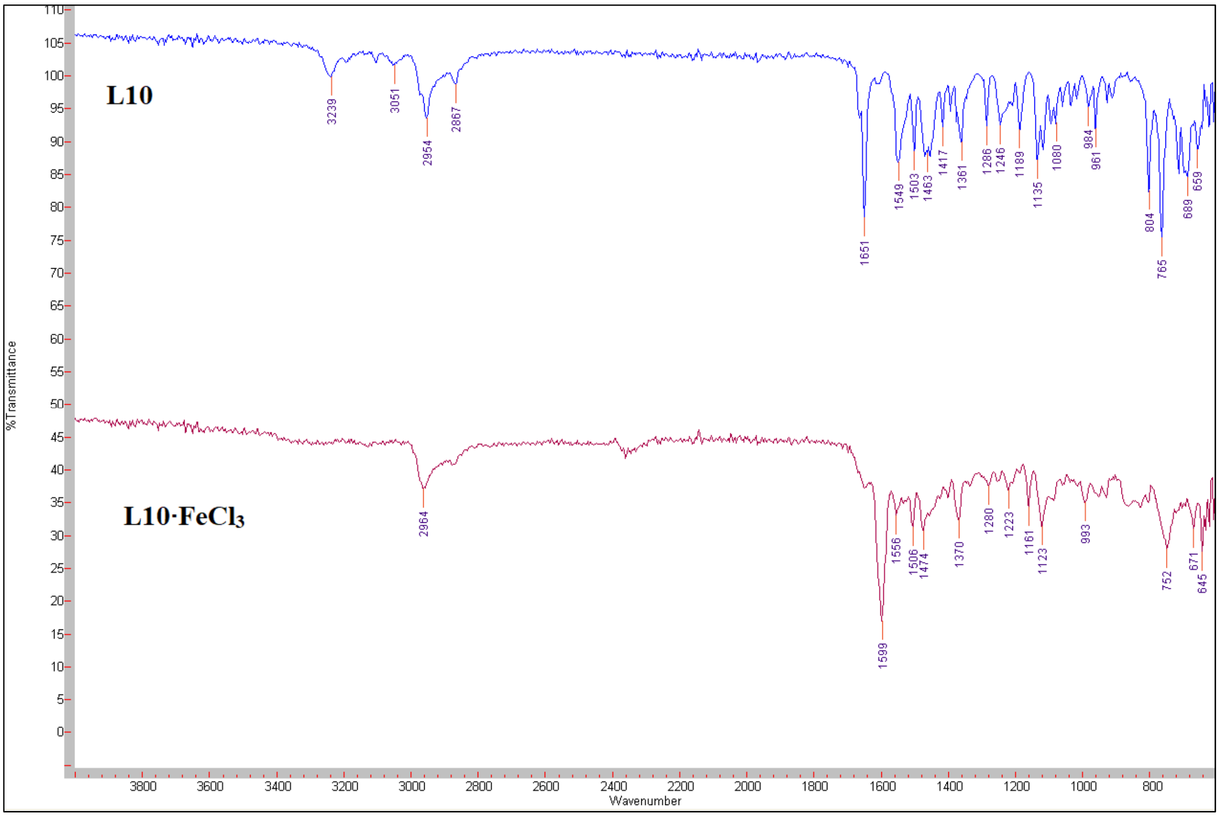

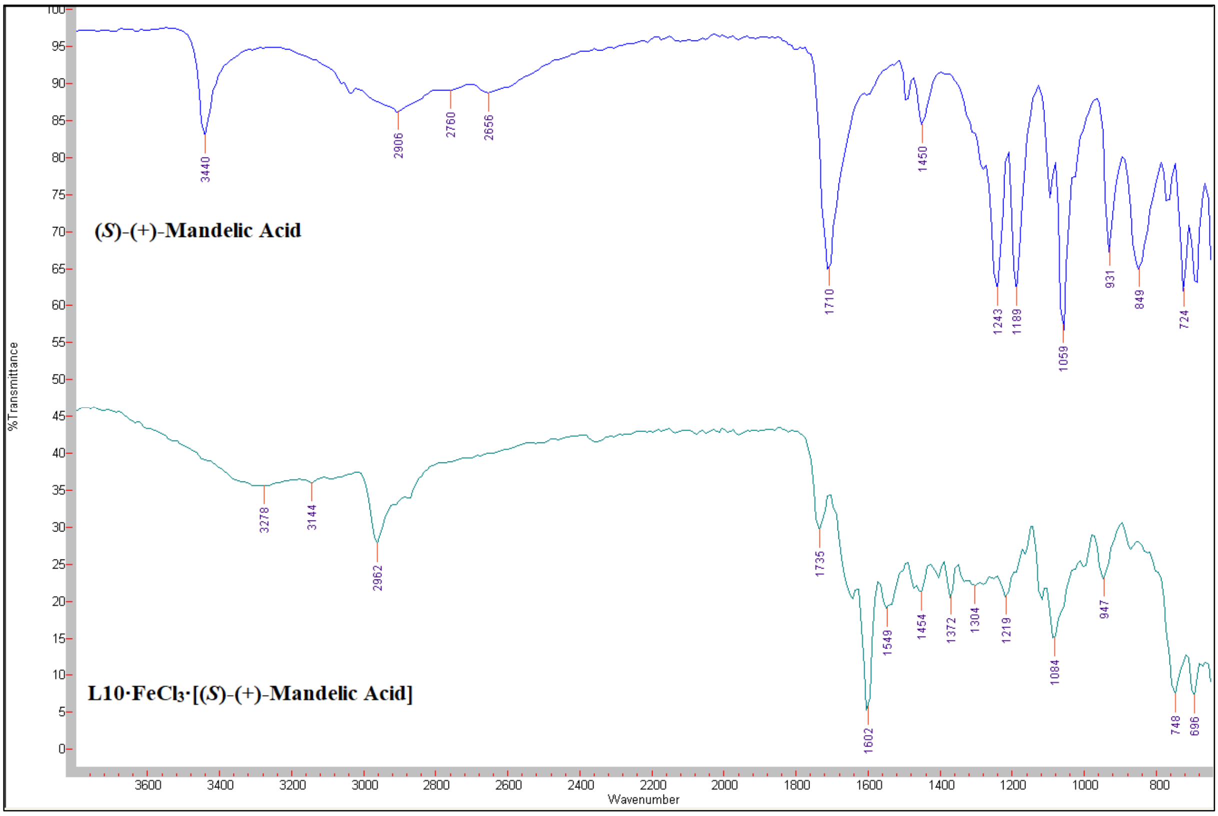

9 NMR Experiments of Fe(III) complex (Jeol ECA 500)

^{1}H, 500 MHz, methanol-d_3

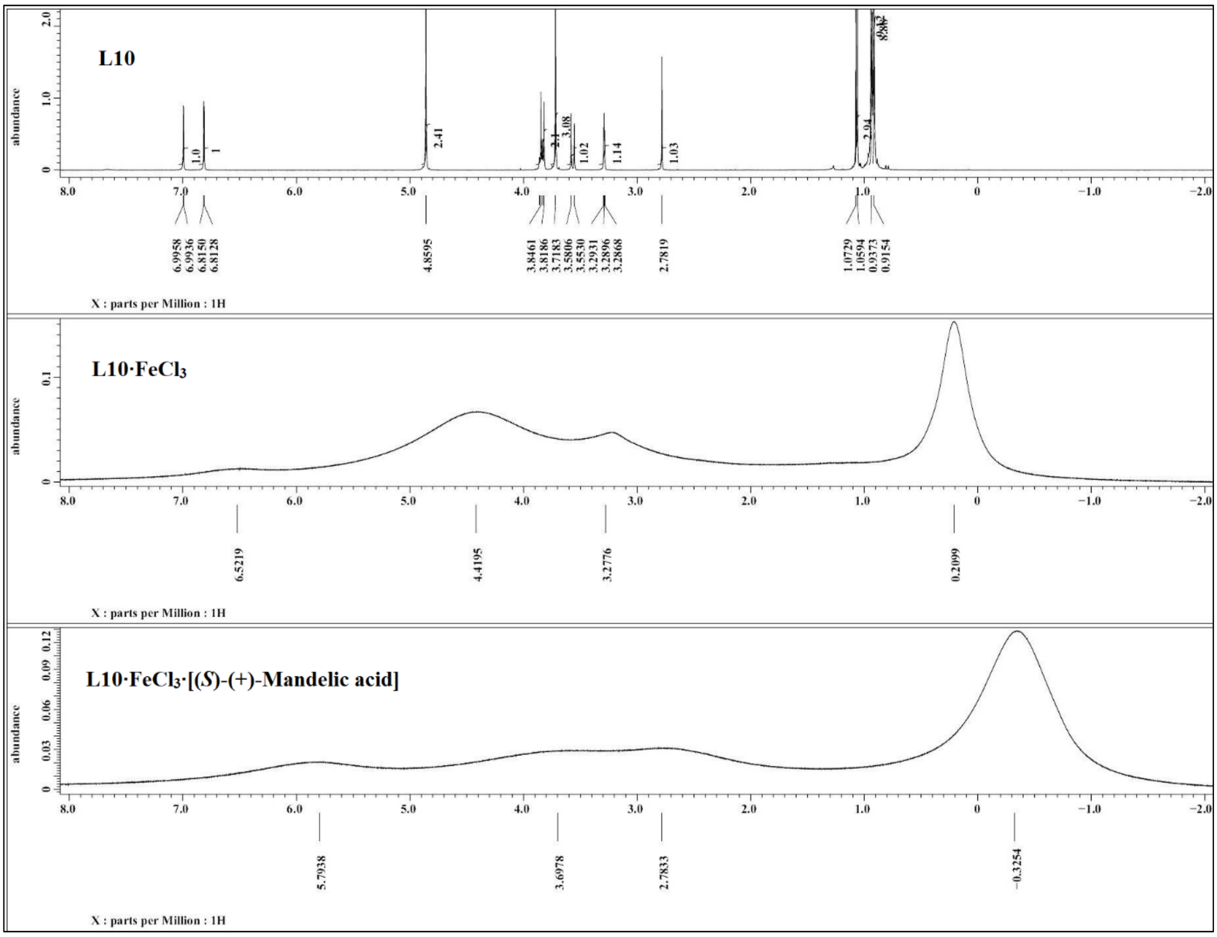

^{13}C, 125.765 MHz, methanol-d_3

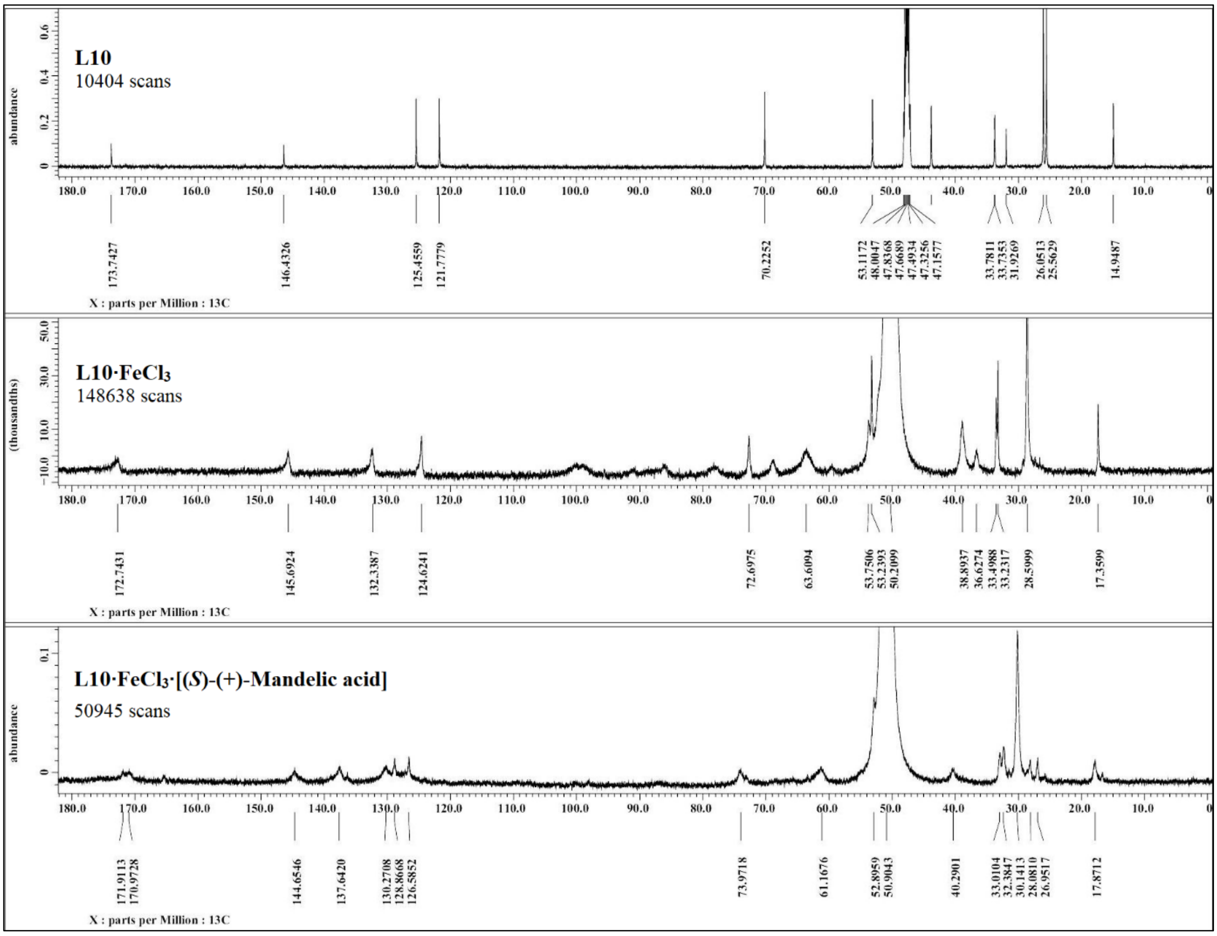

10 MS-ESI Experiment of Fe(III) complex

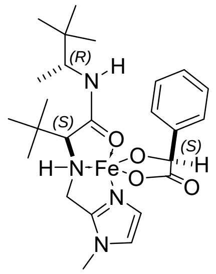

$C_{25}H_{38}FeN_4O_4$
Mol. Wt.: 514.4386

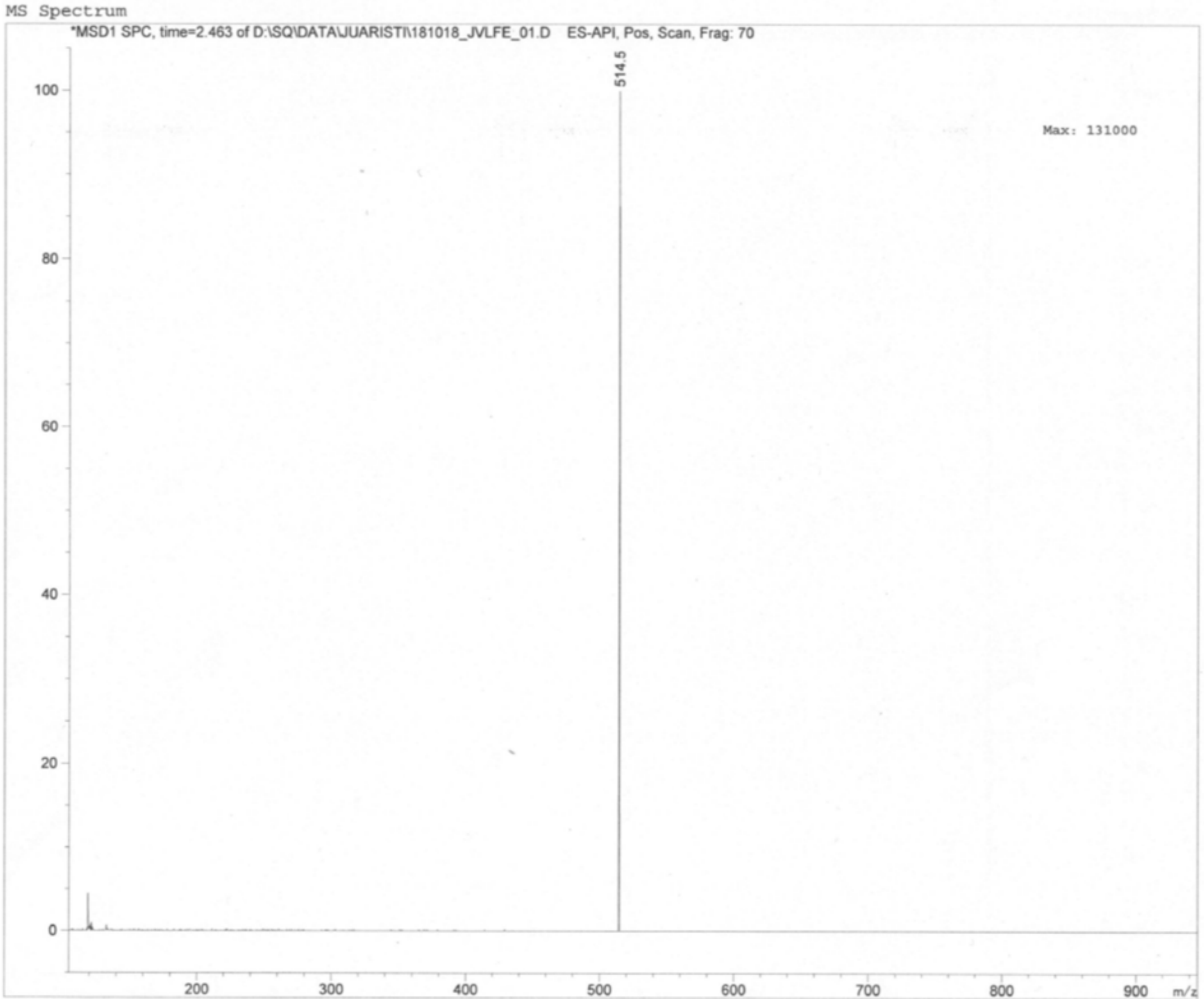

11 X-Ray Crystallographic Data

(*S*,*R*)-**V-(d)**

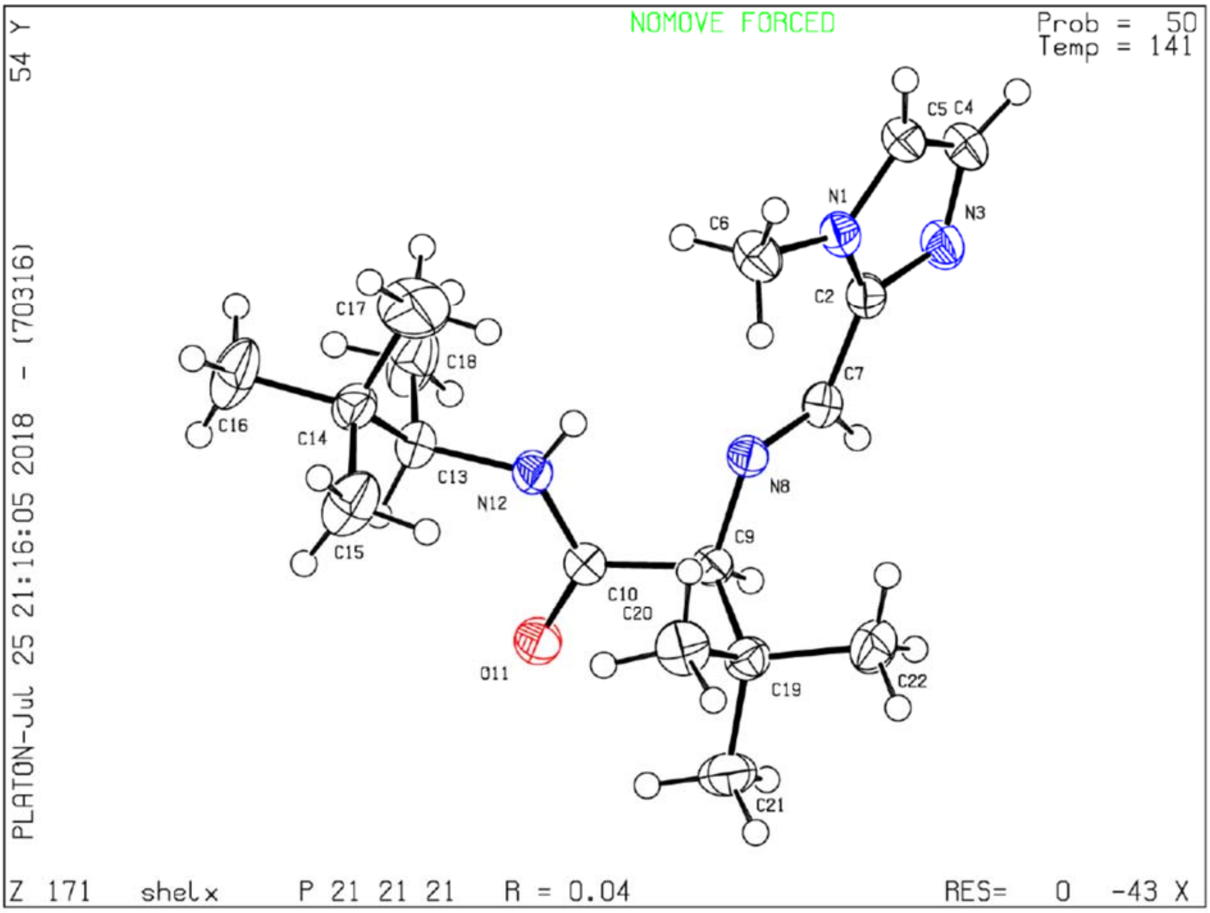

Table A

Experimental details

Crystal data	
Chemical formula	$C_{17}H_{30}N_4O$
M_r	306.45
Crystal system, space group	Orthorhombic, $P2_12_12_1$
Temperature (K)	141
a, *b*, *c* (Å)	6.9534 (3), 14.0923 (5), 18.9394 (7)
V (Å^3)	1855.86 (12)
Z	4
Radiation type	Cu *Kα*
μ (mm^{-1})	0.55
Crystal size (mm)	0.45 × 0.39 × 0.26
Data collection	
Diffractometer	Bruker D8 VENTURE
Absorption correction	Multi-scan<br>*SADABS2016*/2 - Bruker AXS area detector scaling and absorption correction
T_{min}, T_{max}	0.677, 0.754
No. of measured, independent and observed [$I > 2\sigma(I)$] reflections	12734, 3632, 3452
R_{int}	0.035
$(\sin \theta/\lambda)_{max}$ (Å^{-1})	0.618

Table A

Experimental details

Refinement	
$R[F^2 > 2\sigma(F^2)]$, $wR(F^2)$, S	0.038, 0.096, 1.09
No. of reflections	3632
No. of parameters	216
H-atom treatment	H atoms treated by a mixture of independent and constrained refinement
$\Delta\rho_{max}$, $\Delta\rho_{min}$ (e Å^{-3})	0.23, −0.18
Absolute structure	Refined as an inversion twin.
Absolute structure parameter	0.1 (4)

Computer programs: Bruker *APEX3* software, *SAINT* V8.38A integration software, *SORTAV* (Blessing, 1995), *SHELXS2013* (Sheldrick, 2008), *SHELXL2018*/3 (Sheldrick, 2018), *ORTEP-3 for Windows* (Farrugia, 2012), *WinGX* publication routines (Farrugia, 2012).

Table B

Hydrogen-bond geometry (Å, º)

D—H···*A*	*D*—H	H···*A*	*D*···*A*	*D*—H···*A*
C4—H4···O11^{i}	0.95	2.45	3.356 (2)	159
C6—H6*A*···N8	0.98	2.55	3.027 (2)	110
C6—H6*C*···O11^{ii}	0.98	2.54	3.482 (2)	162
N12—H12···N8	0.83 (3)	2.12 (2)	2.597 (2)	117 (2)

Symmetry codes: (i) −*x*+1/2, −*y*+1, *z*+1/2; (ii) −*x*+3/2, −*y*+1, *z*+1/2.

Computing details

Data collection: Bruker *APEX3* software; cell refinement: *SAINT* V8.38A integration software; data reduction: *SORTAV* (Blessing, 1995); program(s) used to solve structure: *SHELXS2013* (Sheldrick, 2008); program(s) used to refine structure: *SHELXL2018*/3 (Sheldrick, 2018); molecular graphics: *ORTEP-3 for Windows* (Farrugia, 2012); software used to prepare material for publication: *WinGX* publication routines (Farrugia, 2012).

(shelx)

Crystal data

$C_{17}H_{30}N_4O$
M_r = 306.45
Orthorhombic, $P2_12_12_1$
Hall symbol: P 2ac 2ab
a = 6.9534 (3) Å
b = 14.0923 (5) Å
c = 18.9394 (7) Å
V = 1855.86 (12) Å^3
Z = 4
$F(000)$ = 672
D_x = 1.097 Mg m^{-3}
Cu $K\alpha$ radiation, λ = 1.54178 Å
Cell parameters from 9103 reflections
θ = 3.9–72.3°
μ = 0.55 mm^{-1}
T = 141 K
Block, colourless
0.45 × 0.39 × 0.26 mm

Data collection

Bruker D8 VENTURE diffractometer
Radiation source: sealed x-ray microsource
Multilayer mirrors monochromator
φ or ω oscillation scans
Absorption correction: multi-scan SADABS2016/2 - Bruker AXS area detector scaling and absorption correcti
T_{min} = 0.677, T_{max} = 0.754
12734 measured reflections
3632 independent reflections
3452 reflections with $I > 2\sigma(I)$
R_{int} = 0.035
θ_{max} = 72.3°, θ_{min} = 3.9°
h = −8→8
k = −17→16
l = −22→23

Refinement

Refinement on F^2
Least-squares matrix: full
$R[F^2 > 2\sigma(F^2)] = 0.038$
$wR(F^2) = 0.096$
$S = 1.09$
3632 reflections
216 parameters
0 restraints
0 constraints
Primary atom site location: dual

Hydrogen site location: mixed
H atoms treated by a mixture of independent and constrained refinement
$w = 1/[\sigma^2(F^2) + (0.0536P)^2 + 0.1847P]$ where $P = (F_o^2 + 2F_c^2)/3$
$(\Delta/\sigma)_{max} < 0.001$
$\Delta\rho_{max} = 0.23$ e Å^{-3}
$\Delta\rho_{min} = -0.18$ e Å^{-3}
Absolute structure: Refined as an inversion twin.
Absolute structure parameter: 0.1 (4)

Special details

Geometry. All e.s.d.'s (except the e.s.d. in the dihedral angle between two l.s. planes) are estimated using the full covariance matrix. The cell e.s.d.'s are taken into account individually in the estimation of e.s.d.'s in distances, angles and torsion angles; correlations between e.s.d.'s in cell parameters are only used when they are defined by crystal symmetry. An approximate (isotropic) treatment of cell e.s.d.'s is used for estimating e.s.d.'s involving l.s. planes.
Refinement. Refined as a 2-component inversion twin.

Fractional atomic coordinates and isotropic or equivalent isotropic displacement parameters (Å^2)

	x	*y*	*z*	U_{iso}*/U_{eq}
C2	0.3729 (3)	0.51502 (13)	0.81217 (10)	0.0274 (4)
C4	0.2396 (3)	0.48785 (15)	0.91227 (11)	0.0371 (4)
H4	0.144996	0.478742	0.947808	0.045*
C5	0.4333 (3)	0.48187 (15)	0.92274 (10)	0.0338 (4)
H5	0.496783	0.46861	0.96604	0.041*
C6	0.7258 (3)	0.49392 (16)	0.84670 (10)	0.0352 (4)
H6A	0.763954	0.544904	0.814422	0.053*
H6B	0.75851	0.432403	0.825763	0.053*
H6C	0.793877	0.501301	0.891665	0.053*
C7	0.3917 (3)	0.53921 (13)	0.73808 (10)	0.0272 (4)
C9	0.5455 (3)	0.56919 (12)	0.63061 (9)	0.0247 (4)
H9	0.408052	0.573869	0.61549	0.03*
C10	0.6423 (2)	0.48977 (13)	0.58749 (9)	0.0248 (3)
C13	0.8078 (3)	0.33434 (13)	0.59699 (10)	0.0300 (4)
H13	0.818462	0.342356	0.544672	0.036*
C14	1.0118 (3)	0.31556 (14)	0.62544 (11)	0.0331 (4)
C15	1.1452 (3)	0.39353 (17)	0.59928 (16)	0.0516 (6)
H15A	1.272017	0.386047	0.621168	0.077*
H15B	1.091777	0.455574	0.611966	0.077*
H15C	1.157823	0.389187	0.547843	0.077*
C16	1.0829 (3)	0.22042 (17)	0.59710 (18)	0.0551 (7)
H16A	1.069924	0.219389	0.545578	0.083*
H16B	1.00607	0.169023	0.61759	0.083*
H16C	1.21828	0.211756	0.609928	0.083*
C17	1.0160 (5)	0.3147 (3)	0.70620 (14)	0.0720 (9)
H17A	1.145193	0.297563	0.722449	0.108*
H17B	0.922929	0.268148	0.723832	0.108*
H17C	0.98247	0.377843	0.72406	0.108*
C18	0.6634 (3)	0.25497 (17)	0.61048 (17)	0.0544 (7)
H18A	0.711664	0.195721	0.589983	0.082*
H18B	0.539995	0.271339	0.588671	0.082*
H18C	0.645806	0.246831	0.66146	0.082*
C19	0.6426 (3)	0.66769 (13)	0.61868 (10)	0.0303 (4)
C20	0.8590 (3)	0.66265 (16)	0.63368 (12)	0.0403 (5)

	x	y	z	U_{iso}*/U_{eq}
H20A	0.880126	0.634832	0.680459	0.06*
H20B	0.913638	0.726724	0.632361	0.06*
H20C	0.921503	0.623174	0.597792	0.06*
C21	0.6082 (4)	0.70108 (16)	0.54278 (12)	0.0447 (5)
H21A	0.672692	0.657792	0.509924	0.067*
H21B	0.660107	0.765247	0.536865	0.067*
H21C	0.469829	0.701575	0.532976	0.067*
C22	0.5494 (3)	0.73969 (15)	0.66897 (12)	0.0417 (5)
H22A	0.576565	0.721556	0.717924	0.062*
H22B	0.41006	0.740513	0.661321	0.062*
H22C	0.602216	0.802981	0.659783	0.062*
N1	0.5181 (2)	0.49860 (11)	0.85900 (8)	0.0280 (3)
N3	0.2014 (2)	0.50889 (13)	0.84311 (9)	0.0339 (4)
N8	0.5499 (2)	0.54364 (11)	0.70524 (8)	0.0257 (3)
N12	0.7308 (2)	0.42302 (12)	0.62562 (8)	0.0286 (3)
O11	0.6345 (2)	0.48868 (10)	0.52280 (7)	0.0374 (3)
H7	0.267 (3)	0.5536 (16)	0.7145 (12)	0.033 (6)*
H12	0.721 (3)	0.4291 (17)	0.6690 (13)	0.035 (6)*

Atomic displacement parameters (Å^2)

	U^{11}	U^{22}	U^{33}	U^{12}	U^{13}	U^{23}
C2	0.0268 (8)	0.0257 (8)	0.0299 (8)	0.0000 (7)	0.0033 (7)	−0.0030 (7)
C4	0.0392 (10)	0.0389 (10)	0.0331 (9)	0.0029 (9)	0.0128 (8)	0.0010 (9)
C5	0.0422 (10)	0.0336 (9)	0.0257 (9)	0.0024 (9)	0.0059 (8)	−0.0005 (8)
C6	0.0261 (9)	0.0481 (12)	0.0315 (9)	0.0003 (8)	−0.0005 (7)	−0.0014 (9)
C7	0.0244 (9)	0.0268 (8)	0.0303 (9)	0.0020 (7)	−0.0002 (7)	−0.0024 (7)
C9	0.0223 (8)	0.0281 (8)	0.0236 (8)	0.0020 (7)	−0.0017 (6)	0.0016 (7)
C10	0.0229 (7)	0.0267 (8)	0.0250 (8)	−0.0016 (7)	−0.0014 (6)	−0.0008 (7)
C13	0.0301 (10)	0.0266 (9)	0.0333 (9)	0.0043 (7)	0.0007 (7)	−0.0022 (8)
C14	0.0295 (9)	0.0306 (9)	0.0393 (10)	0.0049 (8)	0.0009 (8)	0.0036 (8)
C15	0.0342 (11)	0.0348 (11)	0.0858 (18)	−0.0030 (10)	0.0005 (12)	0.0015 (12)
C16	0.0311 (11)	0.0326 (11)	0.102 (2)	0.0059 (9)	0.0098 (12)	−0.0033 (12)
C17	0.0647 (18)	0.105 (3)	0.0463 (14)	0.0288 (18)	−0.0145 (13)	0.0212 (16)
C18	0.0322 (11)	0.0338 (11)	0.097 (2)	0.0000 (9)	0.0089 (12)	−0.0044 (12)
C19	0.0337 (10)	0.0264 (9)	0.0309 (9)	0.0000 (8)	−0.0005 (8)	0.0011 (7)
C20	0.0350 (11)	0.0392 (11)	0.0467 (11)	−0.0094 (9)	0.0005 (9)	−0.0035 (10)
C21	0.0614 (15)	0.0350 (10)	0.0377 (11)	−0.0012 (10)	0.0006 (10)	0.0097 (9)
C22	0.0489 (12)	0.0280 (9)	0.0481 (12)	0.0027 (9)	0.0043 (10)	−0.0035 (9)
N1	0.0289 (7)	0.0285 (8)	0.0267 (7)	0.0000 (6)	0.0027 (6)	−0.0030 (6)
N3	0.0307 (8)	0.0362 (9)	0.0348 (8)	0.0010 (7)	0.0082 (7)	−0.0013 (7)
N8	0.0268 (7)	0.0265 (7)	0.0237 (7)	0.0018 (6)	−0.0009 (6)	−0.0004 (6)
N12	0.0326 (8)	0.0302 (8)	0.0229 (7)	0.0076 (7)	−0.0012 (6)	−0.0013 (6)
O11	0.0494 (8)	0.0394 (8)	0.0235 (6)	0.0102 (7)	−0.0039 (6)	−0.0009 (6)

Geometric parameters

Bond lengths (Å)

C2—N3	1.331 (2)	C14—C17	1.530 (3)
C2—N1	1.364 (2)	C15—H15A	0.98
C2—C7	1.450 (2)	C15—H15B	0.98
C4—C5	1.363 (3)	C15—H15C	0.98
C4—N3	1.369 (3)	C16—H16A	0.98

Bond lengths (Å)			
C4—H4	0.95	C16—H16B	0.98
C5—N1	1.364 (2)	C16—H16C	0.98
C5—H5	0.95	C17—H17A	0.98
C6—N1	1.464 (2)	C17—H17B	0.98
C6—H6A	0.98	C17—H17C	0.98
C6—H6B	0.98	C18—H18A	0.98
C6—H6C	0.98	C18—H18B	0.98
C7—N8	1.265 (2)	C18—H18C	0.98
C7—H7	1.00 (2)	C19—C21	1.531 (3)
C9—N8	1.459 (2)	C19—C20	1.533 (3)
C9—C10	1.540 (2)	C19—C22	1.535 (3)
C9—C19	1.560 (3)	C20—H20A	0.98
C9—H9	1	C20—H20B	0.98
C10—O11	1.227 (2)	C20—H20C	0.98
C10—N12	1.336 (2)	C21—H21A	0.98
C13—N12	1.464 (2)	C21—H21B	0.98
C13—C18	1.525 (3)	C21—H21C	0.98
C13—C14	1.540 (3)	C22—H22A	0.98
C13—H13	1	C22—H22B	0.98
C14—C15	1.521 (3)	C22—H22C	0.98
C14—C16	1.527 (3)	N12—H12	0.83 (3)

Bond Angles (°)			
N3—C2—N1	111.45 (16)	C14—C16—H16C	109.5
N3—C2—C7	121.50 (17)	H16A—C16—H16C	109.5
N1—C2—C7	127.03 (16)	H16B—C16—H16C	109.5
C5—C4—N3	110.12 (17)	C14—C17—H17A	109.5
C5—C4—H4	124.9	C14—C17—H17B	109.5
N3—C4—H4	124.9	H17A—C17—H17B	109.5
C4—C5—N1	106.73 (18)	C14—C17—H17C	109.5
C4—C5—H5	126.6	H17A—C17—H17C	109.5
N1—C5—H5	126.6	H17B—C17—H17C	109.5
N1—C6—H6A	109.5	C13—C18—H18A	109.5
N1—C6—H6B	109.5	C13—C18—H18B	109.5
H6A—C6—H6B	109.5	H18A—C18—H18B	109.5
N1—C6—H6C	109.5	C13—C18—H18C	109.5
H6A—C6—H6C	109.5	H18A—C18—H18C	109.5
H6B—C6—H6C	109.5	H18B—C18—H18C	109.5
N8—C7—C2	124.48 (17)	C21—C19—C20	109.96 (19)
N8—C7—H7	121.9 (13)	C21—C19—C22	108.27 (17)
C2—C7—H7	113.6 (13)	C20—C19—C22	109.26 (18)
N8—C9—C10	108.98 (14)	C21—C19—C9	109.99 (16)
N8—C9—C19	110.54 (14)	C20—C19—C9	110.90 (16)
C10—C9—C19	112.38 (15)	C22—C19—C9	108.40 (16)
N8—C9—H9	108.3	C19—C20—H20A	109.5
C10—C9—H9	108.3	C19—C20—H20B	109.5
C19—C9—H9	108.3	H20A—C20—H20B	109.5
O11—C10—N12	123.46 (17)	C19—C20—H20C	109.5
O11—C10—C9	121.30 (16)	H20A—C20—H20C	109.5
N12—C10—C9	115.23 (15)	H20B—C20—H20C	109.5
N12—C13—C18	108.88 (17)	C19—C21—H21A	109.5

Bond Angles (°)

N12—C13—C14	110.73 (16)	C19—C21—H21B	109.5
C18—C13—C14	114.94 (17)	H21A—C21—H21B	109.5
N12—C13—H13	107.3	C19—C21—H21C	109.5
C18—C13—H13	107.3	H21A—C21—H21C	109.5
C14—C13—H13	107.3	H21B—C21—H21C	109.5
C15—C14—C16	108.80 (18)	C19—C22—H22A	109.5
C15—C14—C17	108.6 (2)	C19—C22—H22B	109.5
C16—C14—C17	109.8 (2)	H22A—C22—H22B	109.5
C15—C14—C13	108.88 (17)	C19—C22—H22C	109.5
C16—C14—C13	109.04 (18)	H22A—C22—H22C	109.5
C17—C14—C13	111.64 (19)	H22B—C22—H22C	109.5
C14—C15—H15A	109.5	C2—N1—C5	106.52 (16)
C14—C15—H15B	109.5	C2—N1—C6	129.38 (15)
H15A—C15—H15B	109.5	C5—N1—C6	124.04 (16)
C14—C15—H15C	109.5	C2—N3—C4	105.17 (17)
H15A—C15—H15C	109.5	C7—N8—C9	118.07 (16)
H15B—C15—H15C	109.5	C10—N12—C13	124.71 (16)
C14—C16—H16A	109.5	C10—N12—H12	115.1 (17)
C14—C16—H16B	109.5	C13—N12—H12	119.1 (17)
H16A—C16—H16B	109.5		

Torsion Angles (°)

N3—C4—C5—N1	−0.5 (2)	C10—C9—C19—C22	−174.58 (15)
N3—C2—C7—N8	−178.00 (19)	N3—C2—N1—C5	−0.4 (2)
N1—C2—C7—N8	3.9 (3)	C7—C2—N1—C5	177.86 (18)
N8—C9—C10—O11	170.40 (17)	N3—C2—N1—C6	176.74 (19)
C19—C9—C10—O11	−66.7 (2)	C7—C2—N1—C6	−5.0 (3)
N8—C9—C10—N12	−8.7 (2)	C4—C5—N1—C2	0.5 (2)
C19—C9—C10—N12	114.21 (18)	C4—C5—N1—C6	−176.78 (19)
N12—C13—C14—C15	−64.0 (2)	N1—C2—N3—C4	0.1 (2)
C18—C13—C14—C15	172.1 (2)	C7—C2—N3—C4	−178.29 (18)
N12—C13—C14—C16	177.39 (18)	C5—C4—N3—C2	0.3 (2)
C18—C13—C14—C16	53.5 (3)	C2—C7—N8—C9	−179.87 (16)
N12—C13—C14—C17	55.9 (3)	C10—C9—N8—C7	−122.76 (17)
C18—C13—C14—C17	−68.0 (3)	C19—C9—N8—C7	113.26 (18)
N8—C9—C19—C21	−170.77 (17)	O11—C10—N12—C13	−8.3 (3)
C10—C9—C19—C21	67.2 (2)	C9—C10—N12—C13	170.72 (16)
N8—C9—C19—C20	67.4 (2)	C18—C13—N12—C10	−98.8 (2)
C10—C9—C19—C20	−54.6 (2)	C14—C13—N12—C10	133.88 (19)
N8—C9—C19—C22	−52.6 (2)		

L10

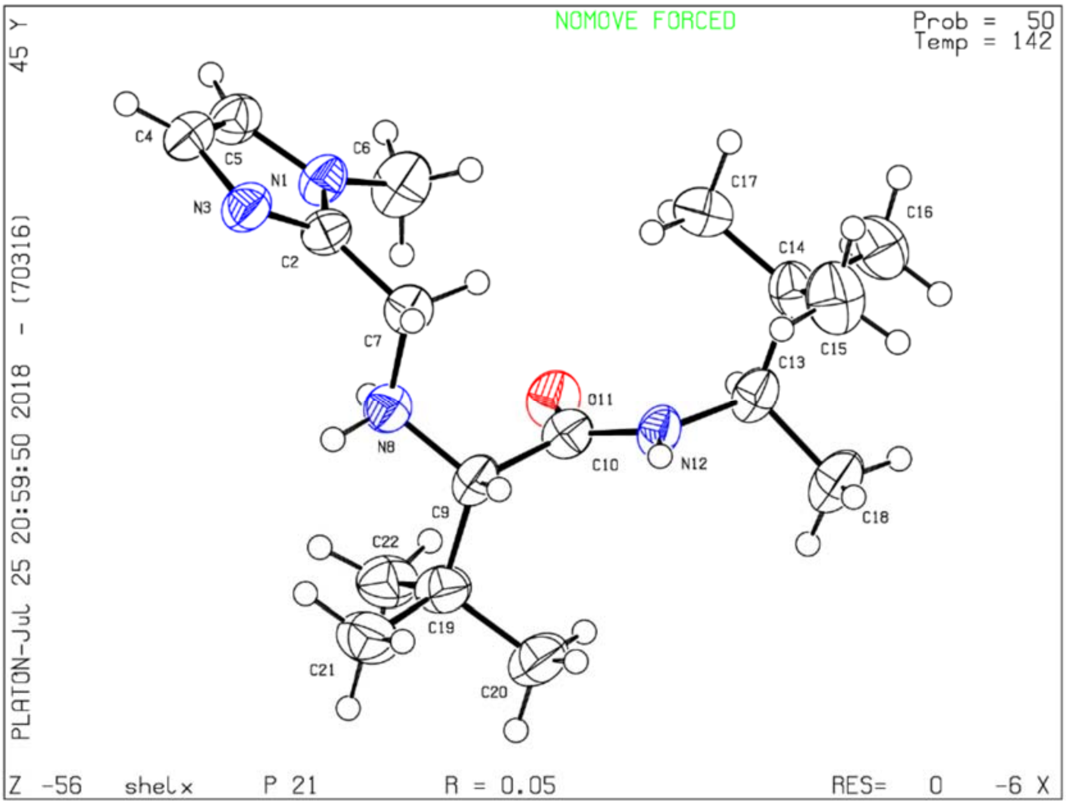

Table A

Experimental details

Crystal data	
Chemical formula	$C_{17}H_{33}N_4O$
M_r	309.47
Crystal system, space group	Monoclinic, $P2_1$
Temperature (K)	142
a, b, c (Å)	8.0138 (5), 13.0776 (8), 9.4290 (6)
β (°)	108.996 (4)
V (Å^3)	934.36 (10)
Z	2
Radiation type	Cu $K\alpha$
μ (mm^{-1})	0.55
Crystal size (mm)	0.32 × 0.17 × 0.11
Data collection	
Diffractometer	Bruker D8 VENTURE
Absorption correction	Multi-scan<br>*SADABS2016*/2 - Bruker AXS area detector scaling and absorption correction
T_{min}, T_{max}	0.575, 0.752
No. of measured, independent and observed [$I > 2\sigma(I)$] reflections	12600, 2823, 2188
R_{int}	0.085
θ_{max} (°)	61.4
$(\sin \theta/\lambda)_{max}$ (Å^{-1})	0.570
Refinement	
$R[F^2 > 2\sigma(F^2)]$, $wR(F^2)$, S	0.053, 0.136, 1.05

Table A

Experimental details

No. of reflections	2823
No. of parameters	212
No. of restraints	1
H-atom treatment	H atoms treated by a mixture of independent and constrained refinement
$\Delta\rho_{max}$, $\Delta\rho_{min}$ (e Å^{-3})	0.14, −0.36
Absolute structure	Flack x determined using 788 quotients [(I+)-(I-)]/[(I+)+(I-)] (Parsons, Flack and Wagner, Acta Cryst. B69 (2013) 249-259).
Absolute structure parameter	0.2 (3)

Computer programs: Bruker *APEX3* software, *SAINT* V8.38A integration software, *SORTAV* (Blessing, 1995), *SHELXS2013* (Sheldrick, 2008), *SHELXL2018*/3 (Sheldrick, 2018), *ORTEP* for Windows (Farrugia, 2012), *WinGX* publication routines (Farrugia, 2012).

Table B

Hydrogen-bond geometry (Å, º)

D—H···*A*	*D*—H	H···*A*	*D*···*A*	*D*—H···*A*
C5—H5···O11^{i}	0.95	2.54	3.461 (6)	162
N12—H12···N3^{ii}	1.00 (6)	2.12 (6)	3.112 (6)	173 (5)

Symmetry codes: (i) $-x$, y+1/2, $-z$; (ii) $-x$+1, y−1/2, $-z$+1.

Computing details

Data collection: Bruker *APEX3* software; cell refinement: *SAINT* V8.38A integration software; data reduction: *SORTAV* (Blessing, 1995); program(s) used to solve structure: *SHELXS2013* (Sheldrick, 2008); program(s) used to refine structure: *SHELXL2018*/3 (Sheldrick, 2018); molecular graphics: *ORTEP* for Windows (Farrugia, 2012); software used to prepare material for publication: *WinGX* publication routines (Farrugia, 2012).

(shelx)

Crystal data

$C_{17}H_{33}N_4O$
M_r = 309.47
Monoclinic, $P2_1$
Hall symbol: P2yb
a = 8.0138 (5) Å
b = 13.0776 (8) Å
c = 9.4290 (6) Å
β = 108.996 (4)°
V = 934.36 (10) Å^3
Z = 2

F(000) = 342
D_x = 1.1 Mg m^{-3}
Cu $K\alpha$ radiation, λ = 1.54178 Å
Cell parameters from 4408 reflections
θ = 5.0–61.0°
μ = 0.55 mm^{-1}
T = 142 K
Nedle, yellow
0.32 × 0.17 × 0.11 mm

Data collection

Bruker D8 VENTURE diffractometer
Radiation source: sealed x-ray microsource
Graphite monochromator
φ or ω oscillation scans
Absorption correction: multi-scan
SADABS2016/2 - Bruker AXS area detector scaling and absorption correction
T_{min} = 0.575, T_{max} = 0.752

12600 measured reflections
2823 independent reflections
2188 reflections with $I > 2\sigma(I)$
R_{int} = 0.085
θ_{max} = 61.4°, θ_{min} = 5.0°
h = −9→9
k = −14→14
l = −10→10

Refinement

Refinement on F^2
Least-squares matrix: full
$R[F^2 > 2\sigma(F^2)] = 0.053$
$wR(F^2) = 0.136$
$S = 1.05$
2823 reflections
212 parameters
1 restraint
0 constraints
Primary atom site location: dual
Secondary atom site location: difference Fourier map
Hydrogen site location: mixed

H atoms treated by a mixture of independent and constrained refinement
$w = 1/[\sigma^2(F_o^{\,2}) + (0.0604P)^2 + 0.2044P]$
where $P = (F_o^2 + 2F_c^2)/3$
$(\Delta/\sigma)_{max} < 0.001$
$\Delta\rho_{max} = 0.14$ e Å^{-3}
$\Delta\rho_{min} = -0.36$ e Å^{-3}
Extinction correction: *SHELXL2018*/3 (Sheldrick 2018), $Fc^*=kFc[1+0.001xFc^2\lambda^3/\sin(2\theta)]^{-1/4}$
Extinction coefficient: 0.015 (2)
Absolute structure: Flack x determined using 788 quotients [(I+)-(I-)]/[(I+)+(I-)]
(Parsons, Flack and Wagner, Acta Cryst. B69 (2013) 249-259).
Absolute structure parameter: 0.2 (3)

Special details

Geometry. All e.s.d.'s (except the e.s.d. in the dihedral angle between two l.s. planes) are estimated using the full covariance matrix. The cell e.s.d.'s are taken into account individually in the estimation of e.s.d.'s in distances, angles and torsion angles; correlations between e.s.d.'s in cell parameters are only used when they are defined by crystal symmetry. An approximate (isotropic) treatment of cell e.s.d.'s is used for estimating e.s.d.'s involving l.s. planes.

Fractional atomic coordinates and isotropic or equivalent isotropic displacement parameters (Å^2)

	x	*y*	*z*	U_{iso}*/U_{eq}
C2	0.3348 (6)	0.6980 (4)	0.3103 (5)	0.0382 (11)
C4	0.3312 (7)	0.8611 (4)	0.2862 (6)	0.0462 (13)
H4	0.35655	0.931443	0.307661	0.055*
C5	0.2177 (7)	0.8236 (4)	0.1544 (6)	0.0455 (13)
H5	0.150157	0.861998	0.069571	0.055*
C6	0.1311 (8)	0.6446 (4)	0.0569 (6)	0.0610 (16)
H6A	0.071904	0.679762	−0.038017	0.092*
H6B	0.043321	0.608073	0.089694	0.092*
H6C	0.216946	0.595744	0.04262	0.092*
C7	0.3643 (6)	0.5916 (4)	0.3689 (5)	0.0402 (12)
H7A	0.375645	0.545345	0.289401	0.048*
H7B	0.475741	0.588434	0.454325	0.048*
C9	0.2174 (7)	0.4479 (3)	0.4451 (5)	0.0399 (12)
H9	0.33545	0.428946	0.517268	0.048*
C10	0.1924 (7)	0.3884 (4)	0.2987 (5)	0.0411 (12)
C13	0.2989 (7)	0.2483 (3)	0.1769 (6)	0.0441 (13)
H13	0.182689	0.263213	0.099002	0.053*
C14	0.4412 (6)	0.2703 (4)	0.1042 (5)	0.0466 (12)
C15	0.6244 (8)	0.2388 (5)	0.2048 (7)	0.0696 (18)
H15A	0.712189	0.259567	0.158441	0.104*
H15B	0.628447	0.164366	0.217997	0.104*
H15C	0.650458	0.272049	0.30275	0.104*
C16	0.3938 (8)	0.2135 (5)	−0.0458 (6)	0.0645 (17)
H16A	0.274134	0.232295	−0.108318	0.097*
H16B	0.399434	0.139552	−0.027595	0.097*
H16C	0.477638	0.232147	−0.097413	0.097*
C17	0.4435 (8)	0.3847 (4)	0.0725 (6)	0.0594 (16)
H17A	0.489048	0.422025	0.167537	0.089*
H17B	0.323367	0.407958	0.01827	0.089*
H17C	0.519617	0.397686	0.011484	0.089*
C18	0.2916 (9)	0.1377 (4)	0.2241 (6)	0.0618 (16)

	x	y	z	U_{iso}*/U_{eq}
H18A	0.277223	0.092684	0.137794	0.093*
H18B	0.191415	0.128578	0.260932	0.093*
H18C	0.401311	0.120407	0.303971	0.093*
C19	0.0762 (7)	0.4195 (4)	0.5182 (5)	0.0473 (13)
C20	0.0639 (8)	0.3039 (4)	0.5296 (7)	0.0593 (15)
H20A	−0.015704	0.286892	0.586023	0.089*
H20B	0.181366	0.275884	0.581448	0.089*
H20C	0.018026	0.274557	0.428684	0.089*
C21	0.1366 (9)	0.4653 (5)	0.6773 (6)	0.0627 (17)
H21A	0.141411	0.540003	0.670733	0.094*
H21B	0.254068	0.439047	0.733583	0.094*
H21C	0.052905	0.44613	0.728686	0.094*
C22	−0.1051 (7)	0.4627 (5)	0.4306 (6)	0.0557 (15)
H22A	−0.193409	0.435466	0.472006	0.084*
H22B	−0.135983	0.442913	0.32489	0.084*
H22C	−0.102243	0.537427	0.438568	0.084*
N1	0.2217 (5)	0.7193 (3)	0.1702 (5)	0.0412 (10)
N3	0.4031 (5)	0.7818 (3)	0.3831 (4)	0.0442 (10)
N8	0.2189 (5)	0.5574 (3)	0.4178 (5)	0.0420 (11)
H8A	0.22442	0.591095	0.503666	0.05*
H8B	0.115367	0.57492	0.346681	0.05*
N12	0.3115 (6)	0.3156 (3)	0.3053 (5)	0.0415 (10)
O11	0.0704 (4)	0.4108 (3)	0.1833 (4)	0.0496 (10)
H12	0.410 (8)	0.308 (5)	0.402 (7)	0.080 (19)*

Atomic displacement parameters (Å^2)

	U^{11}	U^{22}	U^{33}	U^{12}	U^{13}	U^{23}
C2	0.037 (3)	0.038 (3)	0.037 (3)	−0.002 (2)	0.009 (2)	0.003 (2)
C4	0.054 (3)	0.036 (3)	0.050 (3)	0.000 (2)	0.018 (3)	0.006 (2)
C5	0.047 (3)	0.043 (3)	0.044 (3)	0.007 (2)	0.011 (2)	0.011 (2)
C6	0.070 (4)	0.053 (4)	0.043 (3)	−0.002 (3)	−0.004 (3)	−0.002 (3)
C7	0.040 (3)	0.040 (3)	0.038 (3)	0.000 (2)	0.009 (2)	0.000 (2)
C9	0.048 (3)	0.030 (3)	0.036 (3)	0.000 (2)	0.005 (2)	0.001 (2)
C10	0.042 (3)	0.039 (3)	0.039 (3)	−0.003 (2)	0.008 (2)	0.001 (2)
C13	0.052 (3)	0.032 (3)	0.040 (3)	0.000 (2)	0.004 (2)	−0.001 (2)
C14	0.048 (3)	0.048 (3)	0.040 (3)	0.005 (3)	0.010 (2)	−0.005 (2)
C15	0.064 (4)	0.073 (5)	0.064 (4)	0.012 (3)	0.010 (3)	−0.008 (3)
C16	0.073 (4)	0.073 (5)	0.046 (3)	−0.001 (3)	0.018 (3)	−0.012 (3)
C17	0.068 (4)	0.061 (4)	0.059 (3)	−0.004 (3)	0.035 (3)	0.003 (3)
C18	0.090 (4)	0.036 (3)	0.048 (3)	−0.002 (3)	0.007 (3)	0.001 (3)
C19	0.052 (3)	0.047 (3)	0.043 (3)	−0.010 (3)	0.016 (2)	0.001 (2)
C20	0.073 (4)	0.043 (4)	0.061 (4)	−0.010 (3)	0.021 (3)	0.006 (3)
C21	0.077 (4)	0.068 (4)	0.050 (3)	−0.016 (3)	0.030 (3)	−0.007 (3)
C22	0.047 (3)	0.061 (4)	0.061 (4)	−0.004 (3)	0.021 (3)	0.006 (3)
N1	0.044 (2)	0.034 (3)	0.041 (2)	0.0030 (18)	0.0081 (19)	0.0042 (18)
N3	0.052 (2)	0.035 (2)	0.045 (2)	−0.005 (2)	0.0149 (19)	0.000 (2)
N8	0.046 (3)	0.037 (3)	0.043 (2)	−0.0019 (18)	0.015 (2)	−0.0006 (18)
N12	0.052 (3)	0.030 (2)	0.037 (2)	0.001 (2)	0.009 (2)	−0.0033 (17)
O11	0.051 (2)	0.048 (2)	0.041 (2)	0.0042 (17)	0.0036 (17)	0.0014 (17)

Geometric parameters

Bond lengths (Å)

C2—N3	1.314 (6)	C15—H15A	0.98
C2—N1	1.366 (6)	C15—H15B	0.98

Bond lengths (Å)

C2—C7	1.488 (7)	C15—H15C	0.98
C4—C5	1.369 (7)	C16—H16A	0.98
C4—N3	1.378 (6)	C16—H16B	0.98
C4—H4	0.95	C16—H16C	0.98
C5—N1	1.372 (6)	C17—H17A	0.98
C5—H5	0.95	C17—H17B	0.98
C6—N1	1.455 (6)	C17—H17C	0.98
C6—H6A	0.98	C18—H18A	0.98
C6—H6B	0.98	C18—H18B	0.98
C6—H6C	0.98	C18—H18C	0.98
C7—N8	1.456 (6)	C19—C20	1.520 (8)
C7—H7A	0.99	C19—C22	1.527 (7)
C7—H7B	0.99	C19—C21	1.540 (7)
C9—N8	1.456 (6)	C20—H20A	0.98
C9—C10	1.539 (7)	C20—H20B	0.98
C9—C19	1.550 (7)	C20—H20C	0.98
C9—H9	1	C21—H21A	0.98
C10—O11	1.238 (5)	C21—H21B	0.98
C10—N12	1.335 (6)	C21—H21C	0.98
C13—N12	1.473 (6)	C22—H22A	0.98
C13—C18	1.521 (7)	C22—H22B	0.98
C13—C14	1.537 (7)	C22—H22C	0.98
C13—H13	1	N8—H8A	0.91
C14—C15	1.522 (7)	N8—H8B	0.91
C14—C17	1.528 (8)	N12—H12	1.00 (6)
C14—C16	1.532 (7)		

Bond Angles (°)

N3—C2—N1	111.6 (4)	H16A—C16—H16C	109.5
N3—C2—C7	126.7 (4)	H16B—C16—H16C	109.5
N1—C2—C7	121.7 (4)	C14—C17—H17A	109.5
C5—C4—N3	110.1 (5)	C14—C17—H17B	109.5
C5—C4—H4	124.9	H17A—C17—H17B	109.5
N3—C4—H4	124.9	C14—C17—H17C	109.5
C4—C5—N1	105.8 (5)	H17A—C17—H17C	109.5
C4—C5—H5	127.1	H17B—C17—H17C	109.5
N1—C5—H5	127.1	C13—C18—H18A	109.5
N1—C6—H6A	109.5	C13—C18—H18B	109.5
N1—C6—H6B	109.5	H18A—C18—H18B	109.5
H6A—C6—H6B	109.5	C13—C18—H18C	109.5
N1—C6—H6C	109.5	H18A—C18—H18C	109.5
H6A—C6—H6C	109.5	H18B—C18—H18C	109.5
H6B—C6—H6C	109.5	C20—C19—C22	109.5 (5)
N8—C7—C2	111.0 (4)	C20—C19—C21	109.0 (5)
N8—C7—H7A	109.4	C22—C19—C21	109.0 (5)
C2—C7—H7A	109.4	C20—C19—C9	110.1 (4)
N8—C7—H7B	109.4	C22—C19—C9	112.0 (4)
C2—C7—H7B	109.4	C21—C19—C9	107.2 (4)
H7A—C7—H7B	108	C19—C20—H20A	109.5
N8—C9—C10	110.2 (4)	C19—C20—H20B	109.5
N8—C9—C19	111.3 (4)	H20A—C20—H20B	109.5
C10—C9—C19	112.1 (4)	C19—C20—H20C	109.5
N8—C9—H9	107.7	H20A—C20—H20C	109.5
C10—C9—H9	107.7	H20B—C20—H20C	109.5
C19—C9—H9	107.7	C19—C21—H21A	109.5
O11—C10—N12	123.8 (5)	C19—C21—H21B	109.5

Bond Angles (°)

O11—C10—C9	120.3 (5)	H21A—C21—H21B	109.5
N12—C10—C9	115.8 (4)	C19—C21—H21C	109.5
N12—C13—C18	109.1 (4)	H21A—C21—H21C	109.5
N12—C13—C14	113.1 (4)	H21B—C21—H21C	109.5
C18—C13—C14	114.4 (5)	C19—C22—H22A	109.5
N12—C13—H13	106.6	C19—C22—H22B	109.5
C18—C13—H13	106.6	H22A—C22—H22B	109.5
C14—C13—H13	106.6	C19—C22—H22C	109.5
C15—C14—C17	108.5 (5)	H22A—C22—H22C	109.5
C15—C14—C16	110.0 (5)	H22B—C22—H22C	109.5
C17—C14—C16	108.1 (4)	C2—N1—C5	107.0 (4)
C15—C14—C13	112.1 (5)	C2—N1—C6	126.1 (4)
C17—C14—C13	109.2 (4)	C5—N1—C6	126.8 (4)
C16—C14—C13	108.9 (4)	C2—N3—C4	105.6 (4)
C14—C15—H15A	109.5	C9—N8—C7	114.3 (4)
C14—C15—H15B	109.5	C9—N8—H8A	108.7
H15A—C15—H15B	109.5	C7—N8—H8A	108.7
C14—C15—H15C	109.5	C9—N8—H8B	108.7
H15A—C15—H15C	109.5	C7—N8—H8B	108.7
H15B—C15—H15C	109.5	H8A—N8—H8B	107.6
C14—C16—H16A	109.5	C10—N12—C13	122.1 (4)
C14—C16—H16B	109.5	C10—N12—H12	117 (4)
H16A—C16—H16B	109.5	C13—N12—H12	120 (4)
C14—C16—H16C	109.5		

Torsion Angles (°)

N3—C4—C5—N1	−0.7 (6)	C10—C9—C19—C21	168.3 (4)
N3—C2—C7—N8	99.0 (5)	N3—C2—N1—C5	−0.5 (6)
N1—C2—C7—N8	−77.9 (5)	C7—C2—N1—C5	176.8 (5)
N8—C9—C10—O11	−50.8 (6)	N3—C2—N1—C6	176.3 (5)
C19—C9—C10—O11	73.7 (6)	C7—C2—N1—C6	−6.3 (7)
N8—C9—C10—N12	127.4 (5)	C4—C5—N1—C2	0.7 (6)
C19—C9—C10—N12	−108.1 (5)	C4—C5—N1—C6	−176.1 (5)
N12—C13—C14—C15	70.0 (6)	N1—C2—N3—C4	0.1 (5)
C18—C13—C14—C15	−55.8 (6)	C7—C2—N3—C4	−177.1 (5)
N12—C13—C14—C17	−50.2 (5)	C5—C4—N3—C2	0.4 (6)
C18—C13—C14—C17	−176.0 (4)	C10—C9—N8—C7	−63.2 (5)
N12—C13—C14—C16	−168.0 (4)	C19—C9—N8—C7	171.8 (4)
C18—C13—C14—C16	66.2 (6)	C2—C7—N8—C9	167.0 (4)
N8—C9—C19—C20	173.8 (4)	O11—C10—N12—C13	−5.5 (8)
C10—C9—C19—C20	49.9 (6)	C9—C10—N12—C13	176.3 (4)
N8—C9—C19—C22	51.7 (5)	C18—C13—N12—C10	−121.9 (5)
C10—C9—C19—C22	−72.2 (5)	C14—C13—N12—C10	109.5 (5)
N8—C9—C19—C21	−67.8 (5)		
